# A kalihinol analog disrupts apicoplast function and vesicular trafficking in *P. falciparum* malaria

**DOI:** 10.1126/science.adm7966

**Published:** 2024-09-27

**Authors:** Z. Chahine, S. Abel, T. Hollin, G. L. Barnes, J. H. Chung, M. E. Daub, I. Renard, J. Y. Choi, P. Vydyam, A. Pal, M. Alba-Argomaniz, C. A. S. Banks, J. Kirkwood, A. Saraf, I. Camino, P. Castaneda, M. C. Cuevas, J. De Mercado-Arnanz, E. Fernandez-Alvaro, A. Garcia-Perez, N. Ibarz, S. Viera-Morilla, J. Prudhomme, C. J. Joyner, A. K. Bei, L. Florens, C. Ben Mamoun, C. D. Vanderwal, K. G. Le Roch

**Affiliations:** 1 Department of Molecular, Cell and Systems Biology, University of California, Riverside, CA, USA; 2 Department of Chemistry, University of California, Irvine, CA, USA; 3 Department of Internal Medicine, Section of Infectious Diseases, Yale School of Medicine, New Haven, CT, USA; 4 Department of Infectious Diseases, College of Veterinary Medicine, University of Georgia, Athens, GA, USA; 5 Center for Tropical and Emerging Global Diseases, University of Georgia, Athens, GA, USA; 6 Center for Vaccines and Immunology, University of Georgia, Athens, GA, USA; 7 Stowers Institute for Medical Research, Kansas City, MO, USA; 8 Metabolomics Core Facility, University of California, Riverside, CA, USA; 9 GSK, Tres Cantos (Madrid), Spain; 10 Department of Epidemiology of Microbial Diseases, Yale School of Public Health, New Haven, CT, USA; 11 Department of Pharmaceutical Sciences, University of California, Irvine, CA, USA

## Abstract

We report the discovery of MED6-189, an analog of the kalihinol family of isocyanoterpene natural products that is effective against drug-sensitive and drug-resistant *Plasmodium falciparum* strains, blocking both asexual replication and sexual differentiation. In vivo studies using a humanized mouse model of malaria confirm strong efficacy of the compound in animals with no apparent hemolytic activity or toxicity. Complementary chemical, molecular, and genomics analyses revealed that MED6-189 targets the parasite apicoplast and acts by inhibiting lipid biogenesis and cellular trafficking. Genetic analyses revealed that a mutation in *PfSec13*, which encodes a component of the parasite secretory machinery, reduced susceptibility to the drug. Its high potency, excellent therapeutic profile, and distinctive mode of action make MED6-189 an excellent addition to the antimalarial drug pipeline.

## Abstract (summary)

**INTRODUCTION:**

Given widespread resistance to commonly used antimalarials, artemisinin-based combination therapies (ACTs) remain one of the most applicable first-line treatments for drug-resistant malaria. However, recent reports have revealed growing global resistance to ACT partner drugs and clinical treatment failures. Given the finite number of antimalarials and the limited protection offered by current vaccines, there is an urgent need for therapeutics to suppress the spread of the disease. Ideally, candidate molecules should block pathways not previously targeted by approved antimalarials or those in clinical development.

The isocyanoterpene (ICT) family of sponge-derived natural products has shown potent antibacterial, antifungal, and antimalarial activity. Among the ICTs, the kalihinol subfamily, including kalihinols A and B, has shown activity against both drug-sensitive and drug-resistant *Plasmodium falciparum* isolates. However, large-scale biological production of the kalihinol derivatives was complex, and access to efficient chemical biology tools to uncover the mechanism of action of these compounds was limited.

**RATIONALE:**

We chemically synthesized simplified kalihinol analogs in an earlier study. Among these compounds, MED6-189 emerged as a highly potent drug, with median inhibitory concentration values less than 50 nM, against several *P. falciparum* drug-sensitive and drug-resistant strains. Through a sequence of 10 chemical steps, synthesis of MED6-189 was scaled up to support the in-depth discovery of its mechanism of action as well as toxicology and in vivo efficacy studies against human and non-human primate malaria parasites.

**RESULTS:**

MED6-189 inhibited both drug-sensitive and drug-resistant *P. falciparum* strains. Phenotypic assays showed that this compound blocked parasite sexual differentiation, had a promising tolerability profile against human cells, and cleared *P. falciparum* infection in a humanized mouse model. The compound also exhibited potent activity against *P. knowlesi* and *P. cynomolgi*. Microscopy imaging and drug combination studies showed that MED6-189 targeted the apicoplast, a nonphotosynthetic plastid found in most Apicomplexa parasites that is crucial for the synthesis of isoprenoids. Using a multi-omics approach and biotinylated MED6-189 probes that retained antimalarial activity, we gained an understanding of the drug’s mechanism of action and potential resistance pathways. Our findings showed that MED6-189 disrupts apicoplast biology, slows growth, and kills the parasite. Transcriptomic, metabolomic, and proteomic experiments indicated that the compound interferes not only with lipid metabolism but also with vesicular trafficking. Finally, we used prolonged in vitro resistance selection pressure followed by genetic analysis to identify mutations in *Pf Sec13* (PF3D7_1230700), a gene coding for a key component of the protein secretory machinery. Using CRISPR-Cas9 genome editing, we confirmed that mutation of *Pf Sec13* is associated with reduced susceptibility to the drug.

**CONCLUSION:**

Our studies provide a comprehensive investigation into the activity and mode of action of MED6-189, a compound targeting both the asexual and sexual stages of *P. falciparum*. The complex mode of action of MED6-189, along with the challenges faced by the parasite to develop drug resistance, highlights its potential as an antimalarial candidate. Our research indicates that MED6-189, and by proxy other kalihinols and ICTs, is a promising antimalarial drug candidate, particularly in combination with fast-acting therapies. Furthermore, the ability of MED6-189 to inhibit the growth of *P. falciparum*, as well as its efficacy against *P. knowlesi* and *P. cynomolgi*, underscores its potential as a pan-antimalarial lead drug.

## Graphical Abstract

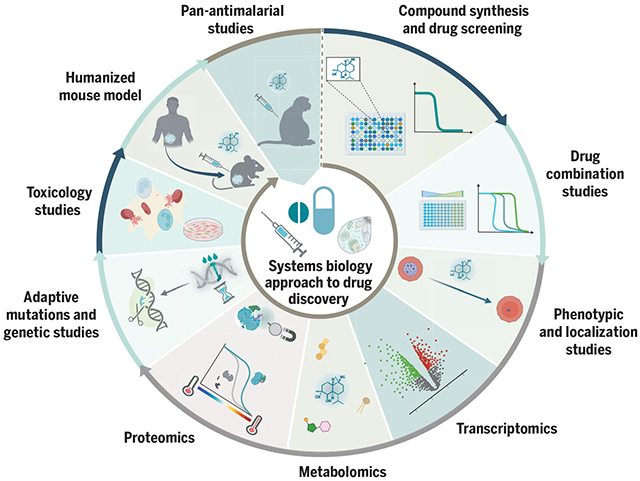

**A systems biology approach for antimalarial drug discovery.** A synthetic analog of a sponge-derived natural product showing antimalarial activity was optimized in a series of phenotypic assays and tested for synergistic effects with known antimalarial drugs. Transcriptomic, metabolomic, proteomic, and genetic analyses identified the parasite target organelle—the apicoplast—a site of parasite isoprenoid synthesis. Pharmacokinetic and toxicity studies across cell types and *Plasmodium* species show feasibility for further development as a pan-*Plasmodium* drug. [Figure created with BioRender.com]

In 2022, an estimated 247 million clinical cases and 619,000 global deaths were attributed to malaria worldwide, most of which were caused by *Plasmodium falciparum* (1). Because of widespread resistance to commonly used antimalarials, artemisinin-based combination therapies (ACTs) represent the last resort in the antimalarial armamentarium for management of drug-resistant malaria (2, 3). Unfortunately, there have been reports of partial resistance to ACTs, particularly in the Greater Mekong region of Southeast Asia and, most recently, emerging cases in parts of Africa (4–6). World Health Organization reports describe resistance to ACT partner drugs as well as clinical treatment failure documented in Southeast Asia. There are few antimalarials with efficacy comparable to that of ACTs, and current vaccines have only limited protective efficacy (7, 8). There is therefore an urgent need to identify novel therapeutics to combat the ever-growing threat of drug resistance and to suppress the spread of the disease. Ideally, such molecules should target pathways not previously targeted by approved antimalarials or those in clinical development.

The isocyanoterpene (ICT) family of sponge-derived natural products encompasses a large number of biosynthetically related isonitrile-, isothiocyanate-, isocyanate-, and formamide-containing diterpenoids, many of which have been shown to have potent antibacterial, antifungal, and antimalarial activity (9–11). The presence of the isonitrile functional group is critical for potent antimalarial activity. Among the ICTs, the kalihinol subfamily contains some of the most active compounds, with kalihinols A (9, 10, 12) and B (Fig. 1A, left) (12–15) exhibiting potent activity against drug-sensitive and drug-resistant *P. falciparum* isolates (14, 15). Our labs previously completed the first synthesis and testing of kalihinol B. We demonstrated that its activity was similar to that of kalihinol A and learned that the oxygen heterocycle motif was likely not critical for activity. We therefore drastically truncated this group, which ultimately led to the discovery of the analog MED6-189 (Fig. 1A, right), whose simplified structure allows for easier synthesis.

Efforts to understand the mechanism of action of compounds in the ICT family go back more than two decades (16, 17). Wright and co-workers showed that some ICTs can bind free heme and inhibit the formation of its crystalline form, hematin (17), suggesting that inhibition of heme detoxification could be an important cause of ICT antimalarial activity (17). This study, however, focused on a specific subgroup of ICTs, namely the tetracyclic cycloamphilectanes and tricyclic amphilectanes, whereas the structurally distinct kalihinols, which inspired the development of MED6-189, were not investigated. More recently, another study showed that compounds in these same two structural classes inhibit both intraerythrocytic replication and liver-stage development of malaria parasites (18), indicating that heme detoxification might not be the primary mechanism by which the larger group of ICTs exert their antiplasmodial activity.

We show that MED6-189, one of our most accessible synthetic analogs of kalihinols A and B, inhibits drug-sensitive and drug-resistant *P. falciparum* strains in vitro, blocks parasite sexual differentiation, and eliminates infection in a humanized mouse model of *P. falciparum* malaria. Closely related chemical probe molecules that retain antimalarial activity were incorporated into a broad systems biology strategy that integrates various multi-omics platforms, leading to an unbiased understanding of both the drug mechanism of action and possible routes of resistance. Our findings demonstrate that MED6-189 (and, by extension, kalihinols and presumably other ICTs) disrupts the apicoplast, an organelle essential for the synthesis of parasite fatty acids and isoprenoids leading to delayed death in the pathogen. Additional metabolomic and proteomic analyses show that the compound also interferes with lipid metabolism and trafficking during the parasite asexual stage. Moreover, we found that mutations in the *PfSec13* gene (PF3D7_1230700), an important component of the protein secretory machinery, is associated with altered susceptibility to the drug. The compound was also found to be potent against other zoonotic *Plasmodium* parasites, *P. knowlesi* and *P. cynomolgi*. Overall, our results show that MED6-189 has an efficient and sophisticated mode of action to which it has proven challenging for the pathogen to develop resistance. These combined characteristics make MED6-189 an optimal candidate for use in combination with fast-acting compounds or as a preventative, with reduced susceptibility for emergence of drug resistance.

## Results

### Generation of synthetic kalihinol analogs

**Fig. 1.**
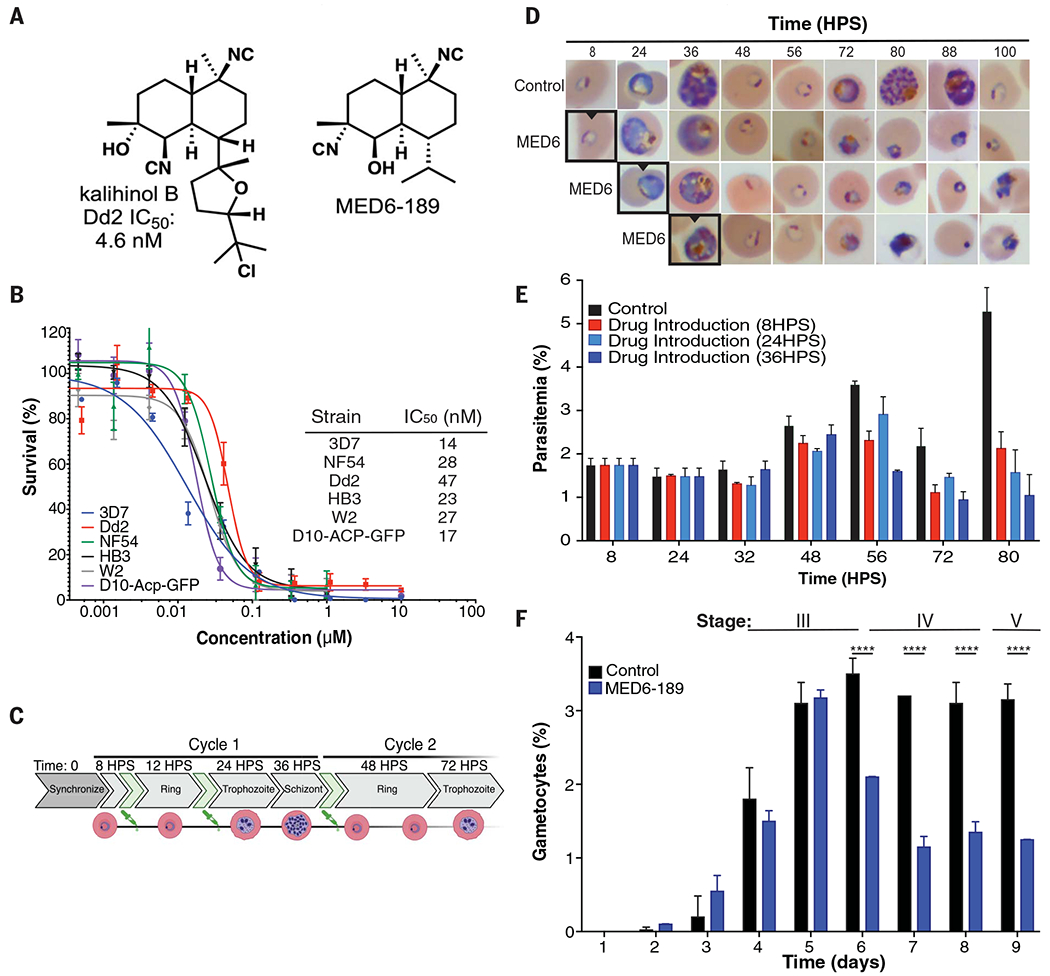
Effect of MED6-189 on *P. falciparum* intraerythrocytic development. (**A**) Chemical structures of the natural product kalihinol B (left) and its analog MED6-189 (right) (14, 15). (**B**) SYBR Green–based dose response assays were conducted on early-ring stage parasites (6 hours after invasion). The parasites were exposed to serial dilutions of MED6-189 for 72 hours, after which parasite growth was assessed. 3D7 WT (blue), NF54 (green), drug-resistant strains Dd2 (red), HB3 (black), W2 (gray), and D10-ACP-GFP (purple) lines (Sigmoidal, 4PL, X is concentration, *n* ≥ 3, nonlinear regression, CI: 95%). (**C**) Schematic diagram of the development of *P. falciparum* after two consecutive erythrocytic cycles. The time points at which MED6-189 was introduced are depicted in green. HPS, hours post synchronization. (**D**) Giemsa-stained images of synchronized 3D7 parasites that were incubated with either DMSO or MED6-189 (at its IC_80_ concentration). The images depict various developmental stages of the parasite’s intraerythrocytic life cycle. Bordered images represent time points when drug was first introduced (*n* = 3 biological replicates, *P* < 0.05). (**E**) Percentage parasitemia after exposure of 3D7 parasites to either DMSO (control) or MED6-189 at various stages of the parasite life cycle within erythrocytes (*P* < 0.05, *n* = 3 biological replicates, two-way ANOVA, Tukey *t* test). (**F**) Inhibition of *P. falciparum* gametocyte development after MED6-189 treatment (300 nM) (blue) during early gametocytogenesis compared with the control (black) (*P* < 0.05, *n* = 3 biological replicates, two-way ANOVA).

As large-scale biological production of ICT natural products is not sustainable, we chemically synthesized kalihinol B (Fig. 1A, left), a tetrahydrofuran isomer of the known potent ICT kalihinol A (a tetrahydropyran). We also demonstrated its activity against *P. falciparum* Dd2 isolate with median inhibitory concentration (IC_50_) values of 4.6 nM (14). Because this compound was challenging to produce on a sufficiently large scale for in-depth mechanism of action studies, several less complex synthetic analogs of kalihinols A and B were generated, and their antiparasitic activity was evaluated in vitro (see supporting information S1 and fig. S1) (14). Among these compounds, MED6-189 emerged as a highly potent, more-accessible drug with IC_50_ values less than 50 nM against several *P. falciparum* strains (Fig. 1A, right) (14). Through a robust sequence of 10 chemical steps, the synthesis of MED6-189 was scaled to support the in-depth studies detailed in this work (see supporting information S1 and fig. S1) (19, 20).

### *MED6-189 affects* P. falciparum *intraerythrocytic life cycle*

To assess the effectiveness of MED6-189 (Fig. 1A, right) on the intraerythrocytic development of *P. falciparum*, we performed growth inhibition assays on several isolates with established sensitivity or resistance to various classes of antimalarials (Fig. 1B). MED6-189 showed excellent activity against both drug-sensitive strains (3D7 and NF54), with IC_50_ values of 14 ± 2 and 28 ± 5 nM, respectively, and pyrimethamine- and chloroquine-resistant strains (HB3, Dd2, and W2) with IC_50_ values of 23 ± 2, 47 ± 7, and 27 ± 5 nM, respectively [GraphPad Prism program: Sigmoidal, 4PL, X is concentration, *n* ≥ 3 biological replicates, non-linear regression, confidence interval (CI): 95%] (Fig. 1B and table S1A). To identify the specific stage of the *P. falciparum* asexual intraerythrocytic cycle (IEC) that was predominantly inhibited by the compound, we carried out phenotypic analyses on synchronized cultures that were incubated with either MED6-189 or a control vehicle [dimethyl sulfoxide (DMSO)] at different time intervals after synchronization (Fig. 1C). Whereas no notable impact of the compound on parasite development could be observed during the first IEC, significant changes including cell cycle arrest and cellular stress were evident during the trophozoite stage of subsequent IEC (Fig. 1D). The treated parasites were unable to fully undergo schizogony or reinvasion [*P* < 0.05, *n* = 3 biological replicates, two-way analysis of variance (ANOVA), Tukey *t* test] (Fig. 1E). This phenotype is reminiscent of that of compounds that target the apicoplast, such as fosmidomycin (21–23). To assess whether MED6-189 could inhibit the parasite’s ability to undergo sexual differentiation, a critical step in malaria transmission, we examined *P. falciparum* gametocyte development in the absence or presence of the compound. Incubation with 300 nM of MED6-189 inhibited early gametocyte development (stage III: days 4 to 6) (Fig. 1F) by ~62% compared with controls (*P* < 0.05, *n* = 3, two-way ANOVA). Furthermore, when exposed to high doses of MED6-189 (1 M) during the early stages of gametocyte development, parasite growth was abolished, and after 48 hours of drug treatment, no quantifiable gametocytes were observed (fig. S2A). In contrast, the compound had little to no effect on mature-stage gametocytes (stage V) (Fig. 1F, fig. S2A, and table S1B). These results suggest a delayed inhibitory activity of MED6-189 on parasite development.

### MED6-189 targets apicoplast metabolism

**Fig. 2.**
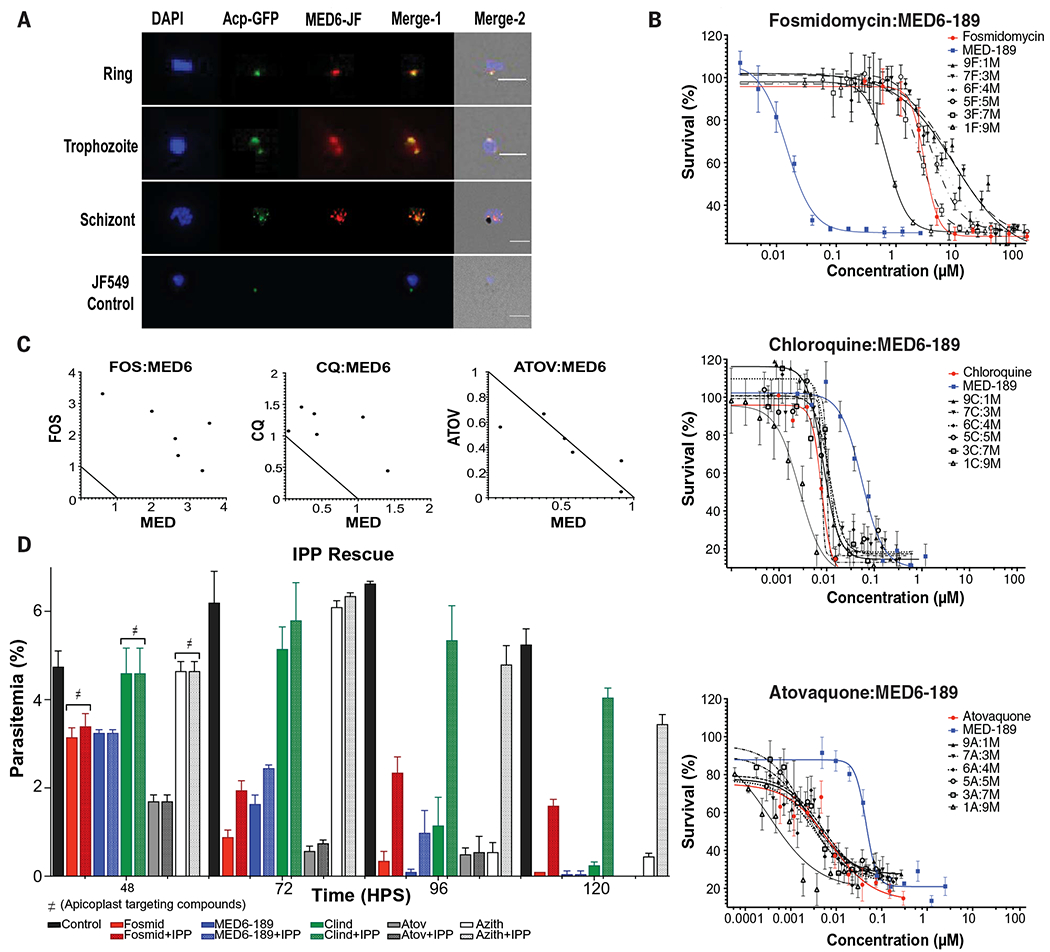
MED6-189 localization and activity in combination with other anti-malarials. (**A**) Cellular localization of MED6-131 (red) in D10-ACP-GFP *P. falciparum* transgenic parasites. Nuclei are stained with 4’,6-diamidino-2-phenylindole (DAPI; blue). Overlap between ACP-GFP (green) and MED6-131 can be seen during the trophozoite and schizont stages of the cell cycle. (**B**) Dose-dependent interactions between MED6-189 (blue) and various antimalarials with known mechanisms of action (red). Logarithmic growth of parasites (*y* axis) is plotted as a function of drug concentrations for MED6-189 (“M”), fosmidomycin (“F”), chloroquine (“C”), or atovaquone (“A”) (*x* axis). The regression line represents a nonlinear regression (variable slope with four parameters), with significant differences considered if *P* < 0.05. Activity correlations between each compound and MED6-189 were analyzed using Pearson correlation (*r*) using GraphPad Prism 9 (GraphPad Software, Inc.), *n* = 3 (see table S2). (**C**) Normalized isobolograms demonstrating drug interaction (synergism, indifference, or antagonism) according to the Loewe additivity model between apicoplast inhibitors and MED6-189. Isobologram curves are expected to be parallel to the diagonal for additive drug pairs, concave for synergistic drug pairs, and convex for antagonistic drug pairs. (**D**) Rescue of 3D7 parasites exposed to DMSO (black), fosmidomycin (red), and MED6-189 (blue), along with several other known antimalarials supplemented with IPP 48 hours after synchronization (dotted). The analysis was performed using a two-way ANOVA, *n* = 3, with *P* < 0.05 significance.

To gain deeper insights into the cellular metabolic target of MED6-189, we synthesized a fluorescently labeled kalihinol analog, MED6-131, featuring a JF549 fluorophore attachment (see MED6-131 structure in fig. S2B) and assessed its activity and cellular localization. Cell growth assays confirmed its activity against the *P. falciparum* D10-ACP-GFP strain (24), exhibiting an IC_50_ of 17 ± 0.9 nM. Fluorescence microscopy analyses were conducted using D10-ACP-GFP transgenic parasites, which express a fusion of the acyl carrier protein (ACP) with green fluorescent protein (GFP) in the parasite apicoplast (25). These analyses revealed the colocalization of MED6-131 with ACP-GFP during the trophozoite and schizont stages of the parasite life cycle (Fig. 2A). These findings affirm localization of the compound within the apicoplast.

To explore whether MED6-189 targets the apicoplast nonmevalonate (MEP/DOXP) pathway, which is essential for parasite survival (23), we initiated drug-drug interaction studies. Our data showed that MED6-189 and fosmidomycin, a known inhibitor of the MEP/DOXP pathway, exhibited antagonistic effects, as indicated by an estimated fractional inhibitory concentration (FIC) index of 2.4 ± 0.36 (Fig. 2, B and C). Interactions of MED6-189 with drugs unrelated to apicoplast functions, namely chloroquine (inhibits heme polymerization in parasite food vacuole) and atovaquone (a mitochondrial electron transport inhibitor), were additive, with FIC scores of 1.71 ± 0.4 for chloroquine and 0.91 ± 0.2 for atovaquone (Fig. 2, Band C, and table S2, A and B) (26, 27).

The killing rate (*k*) of MED6-189– and fosmidomycin-treated parasites was comparable at 48 and 72 hours after treatment. Notably, the *k* value for MED6-189 was significantly higher during the third IEC (table S2C), consistent with the morphological alterations observed after treatment with this compound.

To verify targeting of the apicoplast by MED6-189, we investigated the effect of isopentenyl pyrophosphate (IPP) supplementation on MED6-189 antimalarial activity. IPP is a product of the MEP/DOXP pathway, and its supplementation reduces susceptibility to MEP inhibitors. Synchronized parasites were treated with either vehicle control (DMSO), MED6-189, fosmidomycin, or other known apicoplast-targeting compounds, such as clindamycin and azithromycin, that target protein synthesis in the apicoplast, along with atovaquone, a drug not known to affect the apicoplast (28–30). Parasites were drugged at 80% inhibitory concentrations (IC_80_) for one IEC before IPP (200 μM) supplementation. In contrast to nonsupplemented samples, parasites supplemented with IPP survived the second IEC and initiated a third cycle in the presence of MED6-189 or fosmidomycin (Fig. 2C). Our results show that MED6-189 inhibition can only be rescued by IPP for one additional cycle. Together, these findings indicate that, in addition to inhibition of the apicoplast, MED6-189 likely hinders one or more additional targets within the parasite.

### Multi-omics approaches to unveil the mechanism of action of MED6-189

**Fig. 3.**
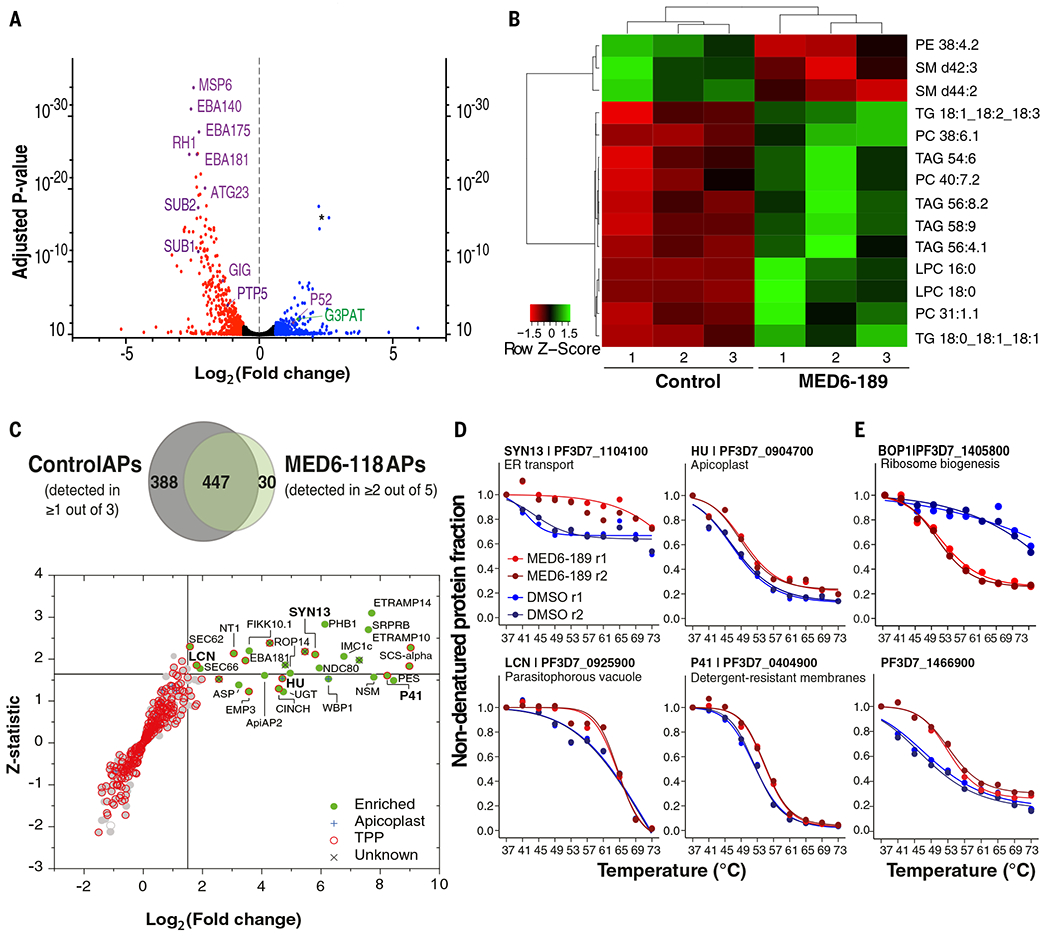
Omics-based profiling of MED6-189–treated parasites. (**A**) Volcano plot representing transcriptomic changes induced by MED6-189 treatment. A total of 5712 transcripts were identified with an adjusted *P*-value cutoff of 0.05. Transcripts associated with invasion and stress responses are highlighted in purple, and those related to apicoplast function are highlighted in green (the asterisk marks noncoding RNAs of unknown function). (**B**) Heatmap depicting the regulation of lipid metabolism in response to MED6-189. Metabolites significantly up-regulated in response to MED6-189 treatment are shown in green, and those down-regulated are shown in red. We used a log_2_ transformation of the data for the calculation of *q* values (Benjamini-Hochberg adjusted *P* values) and *P* values using Welch’s *t* test or ANOVA. (**C**) Protein pulldown assays using biotinylated kalihinol analog, MED6-118. The significance plot displays all proteins detected in at least two of the five independent MED6-118–based affinity purifications (APs). Scatterplots with gray dots depict QPROT-derived log_2_(FC) and *z*-statistic values between MED6-118 APs and negative controls (table S4). Significantly enriched proteins with a log_2_(FC) ≥ 1.5 and a *z* score ≥ 1.645 or those not detected in controls are highlighted in green. Proteins for which thermal profiles are available are shown in red (table S5 and supporting information S2). Proteins localized to the apicoplast are indicated with a blue cross, while proteins of unknown function are marked with a gray “X”. A Venn diagram shows the protein overlap between the MED6-118 APs and controls. (**D**) TPP melting-curve analysis of *P. falciparum* lysates treated with MED6-189. The thermal profiles for four *P. falciparum* proteins significantly enriched in the MED6-118–based pull-downs are shown. (**E**) Thermal profiles for two proteins not detected in MED6-118 pull-down but with significant changes in stability in the presence of MED6-189. For both (D) and (E), stabilization is assessed on the relative amount of soluble protein remaining (*y* axis) after thermal challenge (*x* axis). Sample replicates are color coded in shades of red for MED6-189–treated samples and blue for DMSO controls. Differences in melting temperatures (Δ*T*_m_) along with arrow trends are reported when available (table S6).

To elucidate the cellular and metabolic pathways affected by MED6-189, we used a comprehensive multi-omics strategy. First, we analyzed transcriptional changes by conducting RNA sequencing (RNA-seq) on *P. falciparum* (3D7 strain) in the absence or presence of MED6-189 at various time points during the parasite IEC. Whereas no significant change in transcript levels was detected between the control and drug-treated samples during the first IEC (table S3A), significant changes [log_2_ fold change (FC) of >0.5 or <−0.5] were manifest during the late ring and trophozoite stages of the second IEC, consistent with the delayed inhibitory activity of MED6-189 on parasite development (table S3, A and B). Gene Ontology (GO) enrichment analyses identified several classes of transcripts that were significantly down-regulated after exposure to MED6-189, including those known to be involved in parasite invasion and egress, such as *SUB1* and *SUB2* (PF3D7_0507500 and PF3D7_1136900). These findings indicate that the compound induces cell cycle arrest (fig. S3A). Among the up-regulated genes, we identified those involved in the isoprenoid catabolic process and apicoplast function, such as G3PAT, autophagy, and stress responses (Fig. 3A, table S3C, and fig. S3B) (31). Although most changes detected in transcripts in the second IEC were likely induced by the drug in a nonspecific manner (cell cycle arrest and stress induction), our results also indicate a potential compensatory mechanism of the pathways affecting the isoprenoid catabolic process and the apicoplast.

To assess the effect of MED6-189 on cellular metabolism, highly synchronized parasite cultures were maintained in the absence or presence of the compound, and their metabolomic profiles were examined during the second IEC (72 hours after treatment). A total of 1178 metabolites were detected, of which 40 were significantly affected by MED6-189 treatment (Fig. 3B). These included two polar metabolites [thiamine pyrophosphate (ThPP) and *S*-adenosyl methionine (SAM)] and 38 lipids, including components of the Kennedy pathway for the synthesis of phosphatidylcholine and phosphatidylethanolamine (PC and PE, respectively) (32, 33) (Fig. 3B and fig. S4). TPP is essential for the activity of several enzymes including the apicoplast-associated pyruvate dehydrogenase (PDH) and vitamin B1 biosynthesis (34–37), and SAM is a major precursor for the synthesis of PC from the serine-decarboxylase-phosphoethanolamine-methyltransferase pathway (32, 38). This points to MED6-189 affecting apicoplast biogenesis and membrane biosynthesis.

### *MED6-189 binds to* P. falciparum *membrane trafficking proteins*

To identify proteins that interact with MED6-189, a biotinylated derivative with a similarly potent antimalarial activity (IC_50_ = 92 nM ± 2.0), MED6-118 (fig. S2C), was synthesized and used in pull-down assays using *P. falciparum* protein extracts. The purified proteins were analyzed through multidimensional protein identification technology (MudPIT) and quantified by calculating distributed normalized spectral abundance factor value (39–42) (table S4). More than 450 proteins were detected in at least two of the five biological replicates (Fig. 3C). Among them, 30 proteins were either absent in the negative controls or significantly enriched, as determined by log_2_(FC) ≥ 1.5 and *z*-statistic ≥ 1.645, calculated using QPROT (39, 42). These enriched proteins are involved in vesicular trafficking, lipid biogenesis, and signaling (Fig. 3C and table S4). Specifically, three proteins known to be involved in the endoplasmic reticulum (ER) coat protein complex II (COPII) trafficking systems Sec62 (PF3D7_1438100), syntaxin SYN13 (PF3D7_1104100), a member of the Qa-SNARE family, and a conserved protein recently annotated as a putative translocation protein SEC66 (PF3D7_0204200) were found to be significantly enriched in the pull-down experiments from the MED6-118–treated but not the vehicle control samples (43–47).

To further investigate protein interactions with MED6-189, we conducted thermal proteome profiling (TPP) (48) using whole protein extracts purified from either vehicle or MED6-189–treated parasite cultures (48). After the addition of the ligand, the samples were incubated at temperatures ranging between 37° and 73°C, and soluble proteins were analyzed by liquid chromatography–tandem mass spectrometry (LC-MS/MS; table S5), as described previously (49). The data were processed using the mineCETSA R package (49), from which melting curves were generated for >800 *P. falciparum* proteins (table S6 and supporting information S2) (50, 51). Melting curves were observed for 14 of the 30 proteins enriched in the MED6-118 affinity purification (Fig. 3D, fig. S5A, and table S5). Notably, SYN13 exhibited the most significant shift in melting temperature (Fig. 3D), whereas the other three proteins, namely the apicoplast-localized bacterial histone–like protein (HU; PF3D7_0904700), lipocalin (LCN; PF3D7_0925900), and 6-cysteine protein (P41; PF3D7_0404900), displayed slight differences between the untreated and MED6-189–treated samples.

Other proteins of interest with substantial thermal shifts on MED6-189 treatment included UIS2 (PF3D7_1464600), a serine/threonine protein phosphatase localized to the parasitophorous vacuole, and PyKII (PF3D7_1037100), an apicoplast pyruvate kinase (fig. S5B). Although these proteins were detected in the affinity purification dataset, they did not meet the significance cutoff (table S4). Finally, a few proteins were not detected in the MED6-118 pull-downs but exhibited significant stabilization (PF3D7_0724100 and PF3D7_1466900) or destabilization, in the case of BOP1 (PF3D7_1405800), a protein involved in ribosome biogenesis (Fig. 3E and fig. S5C).

Overall, all but one (PF3D7_0721100) of the proteins we highlighted for their slight-to-marked changes in thermal profiles in the presence of MED6-189 (Fig. 3, D and E, and fig. S5, B and C) did not show any difference in their melting curves in the DMSO and pyrimethamine-treated TPP samples reported by Dziekan *et al.* (49), which likely indicates that these changes in stability are specific to MED6-189 (fig. S6 and table S6). Collectively, these two complementary proteomics approaches suggest a possible mechanism of action of MED6-189 involving the association with, and potential disruption of, the membrane trafficking apparatus between the ER-Golgi or ER-apicoplast systems.

### Selection of MED6-189 refractory parasites

**Fig. 4.**
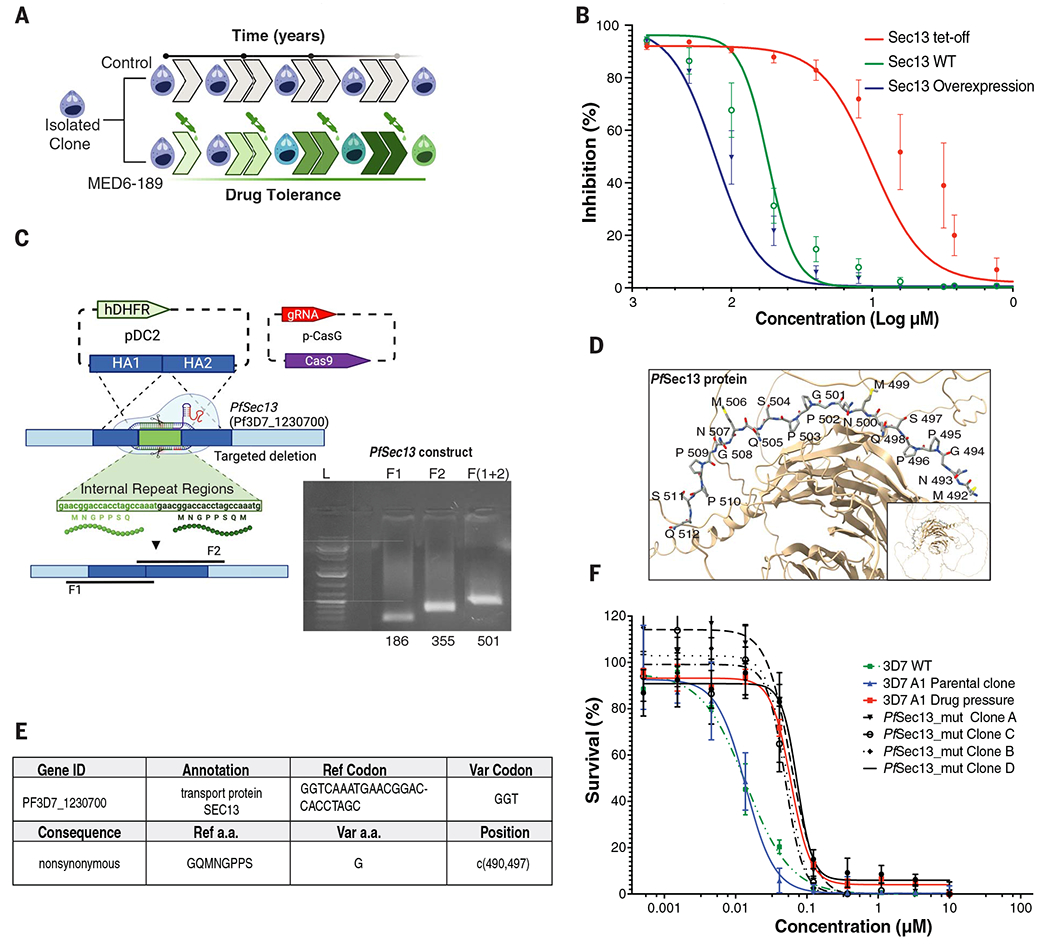
Evidence for a role of *Sec13* in susceptibility to MED6-189. (**A**) Graphical illustration of the methodology used to isolate MED6-189–resistant parasites. (**B**) Comparison of the growth rates of WT *S. cerevisiae* and transgenic clones with either overexpressed or down-regulated Sec13p after treatment with a vehicle (DMSO) or increasing concentrations of MED6-189. (**C**) Schematic representation of the CRISPR-Cas9–based replacement strategy used to introduce a deletion of the targeted repeat regions in the *PfSec13* gene. The insertion was achieved through overlap extension polymerase chain reaction (PCR) of fragments directly upstream and downstream of the target segment and subsequently formed by primer overlap extension PCR to replicate the desired deletion. The insertion was validated through WGS. (**D**) Predicted structure of the SEC13 protein highlighting the seven amino acid tandem repeat regions targeted for deletion (red). Single-letter abbreviations for the amino acid residues are as follows: G, Gly; M, Met; N, Asn; P, Pro; Q, Gln; and S, Ser. (**E**) Results of the sequencing analysis, confirming the successful deletion of the tandem repeat region of *PfSec13* using CRISPR-Cas9 in an isolated clone. a.a., amino acid. (**F**) 3D7 WT and parental lines (green and blue, respectively) and resistant lines (red) maintained in the presence of DMSO or MED6-189 and transgenic *PfSec13*-mut clones (dashed) were subjected to a parasite survival assay. The curves depict parasite survival (*y* axis) in response to serial drug dilution of MED6-189 (*x* axis). Data were analyzed using a Sigmoidal, 4PL (X represents concentration, *n* = 3, nonlinear regression, CI: 95%).

To gain further insights into the cellular machineries affected by MED6-189, we conducted extended in vitro resistance selection experiments (52–54). An initial inoculum of 10^8^ 3D7 parasites was first cloned through serial dilution (55), and isolated clones were split into controls or experimental lines and pulsed with DMSO or MED6-189 at 3× IC_80_ or 5× IC_80_ concentrations for 24 to 48 hours before allowing for parasite recovery. After several failed attempts to select resistance lines, we sought an alternative approach. An inoculum of 10^8^ cloned control and 10^8^ experimental parasites were cultured in the presence of DMSO or MED6-189 at IC_50_ values before gradually escalating the drug dosage (Fig. 4A). After ~36 months of continuous in vitro propagation, parasites exhibiting noticeable drug tolerance emerged. Drug inhibition assays of these selected cloned lines revealed one-half to one-quarter the susceptibility to MED6-189 compared with wild-type (WT) clones (3D7). The IC_50_ value for the drug-sensitive parental line was ~14 ± 1 nM, whereas that for the resistant line was ~60 ± 6 nM (Sigmoidal, 4PL, X is concentration, *n* = 3, nonlinear regression, CI: 95%) (table S1A). Whole-genome sequencing (WGS) identified single-nucleotide polymorphisms (SNPs) or indels (insertions or deletions) in two coding genes, *PfSec13* (PF3D7_1230700) and *Pfendoplasmin* (PF3D7_1222300) (table S7), in the mutant lines but not in the parental lines. However, endoplasmin showed no change in stability compared with DMSO controls in our TPP experiment (fig. S5D). Although *PfSec13* appeared to be the most promising target, owing to its established role in protein trafficking (56, 57), the protein was detected in our TPP assay in presence of MED6-189 but not in our DMSO replicates, likely owing to its low abundance. To assess whether we could use the DMSO melting curves previously established for SEC13 by Dziekan *et al.* (49), we calculated the difference in melting temperatures (Δ*T*_m_) between our DMSO dataset and Dziekan’s for 364 proteins for which *T*_m_ values were confidently calculated by mineCETSA (table S6). Considering the density distribution of the Δ*T*_m_ values between our dataset and Dziekan’s (fig. S5E), a Δ*T*_m_ greater than +15°C between these two independently acquired DMSO datasets. Therefore, Dziekan’s DMSO dataset can be considered a valid proxy and supports that the presence of MED6-189 stabilizes SEC13 beyond its previously observed melting temperature, as no denaturation of SEC13 was observed in our MED6-189 dataset over a 36°C range of temperatures.

The role of the *PfSec13* gene in susceptibility to MED6-189 was confirmed in *Saccharomyces cerevisiae*. As the yeast *sec13* gene is essential for viability, we assessed the effect of MED6-189 after overexpression of Sec13p using a multicopy plasmid or its repression using a tetracycline-regulated (Tet-off) promoter. Sec13p overexpression significantly increased resistance to the drug by 2.4-fold, whereas its repression was accompanied with an ~5.5-fold increase in susceptibility to MED6-189 (Fig. 4B). Alteration of the expression levels of the endoplasmin in yeast did not result in discernible changes in susceptibility to MED6-189 (data not shown).

To validate the role of SEC13 in drug resistance in *P. falciparum*, we used the CRISPR-Cas9 gene editing tool. The seven amino acid deletions detected in the *PfSec13* gene in the resistant clones formed a tandem repeat sequence, which could potentially enhance protein structural integrity or be involved in protein-protein interactions (58). These tandem repeat sequences were targeted for deletion in the 3D7 WT strain [Pf3D7_12_v3: 1261090- 1261110 (+)] (Fig. 4, C and D) using CRISPR-Cas9 (table S8). The targeted deletion was verified through Sanger sequencing and WGS (Fig. 4E and table S7). Four *PfSec13-mut* transgenic clones were tested for their sensitivity to MED6-189. All *PfSec13-mut* clones exhibited tolerance levels to MED6-189 similar to those of the MED6-189 resistant lines obtained after drug selection (IC_50_ range: 50 to 73 nM, SEM ± 9.0 to 10 nM) compared with the WT strain (Fig. 4F and table S1A). Collectively, these genetic and pharmacological studies in both *P. falciparum* and *S. cerevisiae* underscore the crucial role of SEC13 in susceptibility to MED6-189.

### MED6-189 exhibits a favorable safety and tolerability profile

The potency of MED6-189 against *P. falciparum* in vitro coupled with a comprehensive understanding of its mechanism of action prompted us to explore its efficacy in an animal model of *P. falciparum* malaria. The in vitro safety profile of MED6-189 was first determined by assessing possible effects on the growth and metabolic activity of five human cell lines (HeLa, THP1, HEK293, HepG2, and hTERT) at concentrations ranging from 48 nM to 100 μM. No inhibitory activity could be found, resulting in an estimated in vitro therapeutic index of >500, surpassing that of several approved antimalarial drugs (table S8). MED6-189 was further evaluated in 15 in vitro assays using the Enhanced Cross Screen Panel (eXP), which provides pharmacological information and toxicology alerts for the target compound. Of these, hPXR (human pregnane X receptor) IC_50_ 7.4 μM and BSEP (bile salt export pump) inhibition IC_50_ 50 μM were highlighted as factors to be considered in future drug optimization studies aimed at identifying ideal partner drugs and mitigating potential hepatotoxicity. Given the susceptibility of isonitrile compounds to hydrolysis to formamides in acidic aqueous solution, we synthesized the formamide derivatives GB209-2 and GB209-3 by acidic hydrolysis of MED6-189 (see supporting information S1). These derivatives exhibited significantly decreased antiplasmodial activities (IC_50_ values of 3.8 and 3.9 μM, respectively), indicating that these potential degradation products are not responsible for the observed antimalarial effects of MED6-189 administration. We also showed that MED6-189 had reasonable stability to aqueous acidic conditions that mimic the gut (see supporting information S1). Furthermore, MED6-189, GB209-2, and GB209-3 showed no hemolytic activity at concentrations up to 10 μM (see supporting information S1 and figs. S7 and S8). To guide in vivo efficacy studies, we investigated the in vivo tolerability profile of MED6-189. Our data showed that animals treated with MED6-189 at doses up to 50 mg/kg did not exhibit any adverse events, and there were no significant changes in hematology, clinical chemistry analyses, or necropsy observations (table S9). Notably, the data also indicated tolerable exposure levels and compound absorption.

### *In vivo MED6-189 efficacy in a humanized mouse model of* P. falciparum *malaria*

**Fig. 5.**
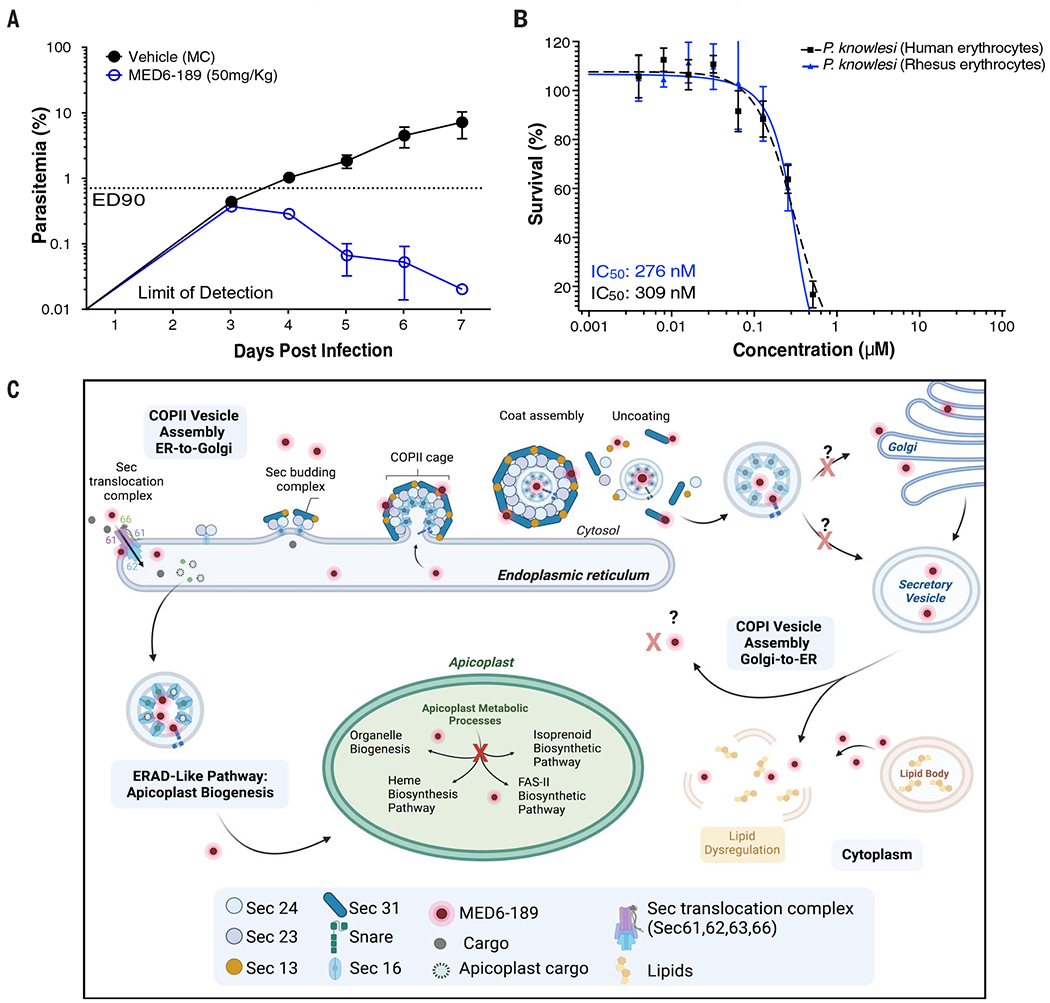
In vivo and broad-spectrum antimalarial efficacy of MED6-189. (**A**) The in vivo efficacy of MED6-189 was evaluated in a humanized mouse model infected with *P. falciparum* (blue) compared with untreated controls (black). (**B**) Dose-dependent response of MED6-189 on *P. knowlesi* YH1 human erythrocyte infecting (black) and rhesus erythrocyte infecting (blue) parasites. The graphs illustrate the logarithmic growth of parasites (*y* axis) in response to varying drug concentrations (*x* axis). Error bars represent standard deviations from two independent experiments conducted in triplicate. The regression line is derived from a nonlinear regression analysis (variable slope with four parameters, least squares fit). (**C**) Proposed mode of action of MED6-189 in *P. falciparum*–infected erythrocytes. The compound is imported into the ER by the Sec translocation complex (SEC61, SEC62, SEC63, SEC66), where it interacts with components of the ER transport machinery. The compound is translocated into the apicoplast, where it directly interacts with proteins involved in crucial apicoplast function, ultimately disrupting this vital organelle. The interactions of MED6-189 with components of the apicoplast function and trafficking systems lead to dysregulation of lipids, resulting in the disruption of key biological processes within the *Plasmodium* parasite.

Owing to the favorable safety and tolerability profile exhibited by MED6-189, we chose a dosage of 50 mg/kg to assess the in vivo efficacy of the compound using a humanized mouse model of *P. falciparum* malaria (59). NOD scid gamma (NSG) mice were intravenously engrafted with human red blood cells (hRBCs) daily until the percentage of hRBCs reached ~50%, at which point the mice were infected with 2 × 10^7^ *P. falciparum*–infected erythrocytes. On the third day after infection, mice were administered either the vehicle alone or MED6-189 at 50 mg/kg orally, once a day for 4 days. Whereas the control group showed a rapid increase in parasite load, with parasitemia reaching ~6% by day 6 after infection, mice treated with MED6-189 showed significant clearance of parasites, achieving >90% reduction in parasitemia inthe peripheral blood by the seventh day after treatment (Fig. 5A and table S10).

### Pan-antimalarial activity of MED6-189

Given the favorable biological activity of MED6-189 against *P. falciparum*, we also assessed its effectiveness against other *Plasmodium* species that infect humans. Owing to the lack of robust in vitro culturing systems for *P. vivax*, we examined the efficacy of MED6-189 against *P. knowlesi* and *P. cynomolgi*, which infect human erythrocytes and are commonly used as model systems for *P. vivax* infection (60). *P. knowlesi*, an Asian Old World monkey parasite with a robust in vitro culture system, is known to be zoonotic for humans. *P. cynomolgi*, a simian parasite capable of infecting humans experimentally, is phylogenetically closely related to *P. vivax* and shares similar biological and genetic properties with *P. vivax* (60). MED6-189 demonstrated significant activity against both parasites, with IC_50_ values of 309 ± 42 and 276 ± 22 nM against *P. knowlesi* in human and rhesus erythrocytes, respectively, and 136 ± 56 nM (*n* = 3) against *P. cynomolgi* (Fig. 5B and figs. S9 and S10, A and B) (Sigmoidal, 4PL, X is concentration, *n* = 3, nonlinear regression, CI: 95%). Despite the increased IC_50_ values against *P. cynomolgi* and *P. knowlesi* compared with those obtained for *P. falciparum*, the values fall well within the low nanomolar range. Differences in drug efficacy is not uncommon among *Plasmodium* species and is most likely caused by the ability of *P. falciparum* to transport and concentrate nutrients and other molecules, including antimalarials, at a high level inside the parasite. Together, the data validate MED6-189 as a potent antimalarial with broad-spectrum activity that targets both *falciparum* and non-*falciparum* human malaria.

## Discussion

Using a multifaceted approach, we have investigated the in vitro and in vivo efficacy, mode of action, resistance mechanisms, and preclinical safety profile of the kalihinol analog MED6-189. Our findings establish MED6-189 as a multitarget compound with limited capacity for the parasite to develop drug resistance, providing a promising new lead in the fight against malaria. Our studies further highlight the pivotal role of the apicoplast in the biological activity of the drug. Using both targeted and untargeted analyses, we have confirmed the apicoplast as a major target of its mode of action. Furthermore, our investigations have unveiled a delayed death mode of operation for MED6-189, reminiscent of other small molecules that target the integrity and biogenesis of the apicoplast.

To localize MED6-189 within the parasites, we used a fluorescent analog, which confirmed the apicoplast as a major site of MED6-189 accumulation. Consistent with this finding, the compound was found to have a slow killing mode of action, like that of several classes of apicoplast-targeting molecules (21, 22, 61). Moreover, in vitro efficacy studies show that the activity of MED6-189 was antagonistic to that of fosmidomycin, a drug previously shown to disrupt the MEP pathway in the apicoplast. Notably, metabolic supplementation with IPP led to a partial rescue of parasite replication. Unlike fosmidomycin, MED6-189–treated parasites did not progress past the third IEC, suggesting that the compound may have multiple mechanisms of action, which may also account for the challenges in obtaining resistant parasites through standard drug-selection methods.

Our metabolite profiling identified significant alterations in lysophosphatidylcholine (LPC), several triglycerides or triacylglycerides (TG or TAGs), and a limited subset of phosphatidylcholine (PC) forms in MED6-189–treated parasites. Notably, LPC exhibited the most pronounced change, with an estimated 3.5-fold increase in its steady-state levels. LPC, a precursor for the biosynthesis of PC, the most abundant phospholipid (50% of phospholipids in *P. falciparum* membranes), plays a key role in the regulation of sexual differentiation (62). Our data also indicated a significant reduction in the biosynthesis of sphingomyelin (37, 63, 64), sterol-esters, and phosphatidylethanolamine (PE) in MED6-189–treated samples, indicating a significant disruption in lipid metabolism and turnover caused by the compound. Our metabolomic analysis identified ThPP as a major altered metabolite in *P. falciparum* after treatment with MED6-189. ThPP is essential for the activity of several enzymes and biosynthetic pathways, including the PDH complex found in the apicoplast (36). The apicoplast PDH converts pyruvate into acetyl–coenzyme A (CoA), the major fatty acid precursor, whereas a second distinct PDH fuels the tricarboxylic acid cycle in the mitochondria (65). Altogether, our data indicate that MED6-189 operates by modulating *P. falciparum* lipid metabolism and apicoplast biogenesis.

Proteomic analyses further supported the link between MED6-189 activity and *P. falciparum* membrane biogenesis and trafficking pathways. Several components of the SEC and SNARE secretory machinery, namely (PF3D7_1230700), SEC62 (PF3D7_1438100), SEC66 (PF3D7_0204200), and syntaxin SYN13 (PF3D7_1104100), were found either to be enriched in the drug pulldown experiments and/or showed differential melting profiles between MED6-189–treated versus vehicle-treated control samples. However, not all targets identified in the pull-down assays were also identified by TPP. With relatively fewer proteins of significance found stabilized in our TPP dataset, one may hypothesize that MED6-189 may have greater impact on the ribosomal RNA, which is not captured in TPP results. Other factors can account for the differences seen between TPP and affinity purification findings, including the level of abundance of specific proteins. For example, the SEC13 protein melting curves were not identified in our DMSO control samples yet were consistently detected in our MED6-189 samples (fig. S5D). The problem with low-abundance target proteins not generating TPP curves has been noted previously by Mateus and co-workers (66). However, the melting curves of SEC13 in the presence of DMSO and pyrimethamine have been previously established by Dziekan *et al.* (49), and we showed that using this DMSO thermal profile (fig. S5D) was a reasonable comparison (fig. S5E). Combining these TPP analyses indicated that MED6-189 did indeed greatly stabilize SEC13 beyond 49°C, its previously observed melting temperature (fig. S5D) (49), indicating a likely direct interaction with the drug.

SEC13 plays a major role in COPII-mediated vesicular transport between the nuclear pore complex, ER, and Golgi membranes, whereas SEC62 and SEC66 (SEC71) are important components of protein translocation machinery in the ER (45, 46, 56, 57, 67). The ER-associated degradation system (ERAD) is a quality-control mechanism that retrotranslocates misfolded secretory proteins across the ER. A similar system, the ERAD-like system, is believed to play a critical role in protein transport into the apicoplast (68, 69). Both the thermal proteomic profiling and pull-down assays identified significant interactions between MED6-189 and the SNARE protein family. Notably, syntaxin SYN13, a Qa-SNARE family protein, was also identified in our TPP assay as *P. falciparum* proteins specifically stabilized by MED6-189. The role of these proteins in COPI and COPII vesicle trafficking has previously been reported (70–72). These proteins play a key role in membrane identification and mediate membrane fusions across various organelles including the ER, mitochondria, apicoplast, and other double-membrane–bound vesicles (70, 71, 73–75). Most recently, SNARE proteins have been shown to play an important role in the biogenesis and maintenance of the apicoplast organelle in *Toxoplasma gondii* (76).

Other evidence for the disruption of apicoplast biogenesis stems from the thermal shift assay that revealed that MED6-189 significantly affected the stabilization of proteins such as 2*C*-methyl-d-erythritol 2,4-cyclodiphosphate enzyme (MECP, PF3D7_0209300), single-stranded DNA binding protein (SSB, PF3D7_0508800), and acetyl-CoA binding proteins (PF3D7_0810000) (fig. S5C, bottom row). All these proteins are integral to the biosynthesis of terpenoids (77), fatty acids, and apicoplast biogenesis systems (68, 69, 77).

Using omics-based strategies, we have gained an understanding of how MED6-189 is trafficked into the apicoplast and disrupts its biogenesis, ultimately leading to parasite cell death consistent with other delayed-action apicoplast-disrupting therapies such as fosmidomycin (Fig. 5C). These studies were further complemented by genetic approaches aimed at selecting parasite clones with reduced susceptibility to MED6-189. Initial attempts to generate resistant variants through the one-step selection approach had failed (78, 79), yet we were ultimately able to achieve heightened drug tolerance in culture by incrementally escalating drug dosage over time through a stepwise selection method (80, 81). Unlike many other antimalarials for which resistant parasites can be selected within a small number of IECs, parasites with enhanced tolerance to MED6-189 required an estimated 36 consecutive months of drug pressure. The IC_50_ for MED6-189 in these resistant parasites was determined to be three-to fourfould that of the parent 3D7 strain. WGS of the resistant clones identified SNP and indel mutations in genes involved in vesicular trafficking, including *PfSec13* and *Pfendoplasmin*. Because *Pfendoplasmin* showed no change in stability compared with DMSO controls in our TPP experiment, we validated *PfSec13* as a potential drug target. The importance of SEC13 in MED6-189 cellular response was therefore further validated using yeast as a model system. This finding led us to apply the CRISPR-Cas9 genetic editing tool in *P. falciparum* to validate the role of SEC13 in MED6-189 tolerance. *PfSec13-mut* lines were subsequently cloned, and survival assays confirmed the role of SEC13 as an adaptive mechanism of resistance to MED6-189 treatment. Our proteomics analyses pointed to a direct interaction between SEC13 and MED6-189 and indicate that both the mode of action and mechanism of resistance of the drug are most likely linked. One of the stronger limitations of our studies is the fact that we were unable to obtain a fully resistant line, limiting our capability to fully understand the mechanism of action and resistance of MED6-189.

Altogether, our studies provide a comprehensive investigation into the activity and mode of action of MED6-189, a highly effective antimalarial compound targeting both the asexual and sexual stages of *P. falciparum*. The array of complex molecular pathways hindered by MED6-189 and the difficulty of selecting parasites that are refractory to the compound render it an exciting candidate for preventing parasite progression with reduced susceptibility for emergence and spread of drug resistance. While our studies show that MED6-189 exhibits polypharmacology by targeting apicoplast biogenesis and fatty acid biosynthesis and trafficking, other possible contributing mechanisms of action, including inhibition of heme detoxification, remain possible. Despite our comprehensive investigation, we have yet to fully understand explicit biochemical pathways affected by MED6-189. As a result, further exploration into the pathogen’s secretory pathways and the roles they play in lipid trafficking are clearly warranted. We establish that MED6-189 and, by extension, other similar kalihinols and ICTs more broadly may serve as promising antimalarial agents, especially when coupled with more fastacting drug therapies. Finally, the ability of MED6-189 to inhibit the growth of *P. falciparum* in vitro and in humanized mice and to inhibit the growth of *P. knowlesi* and *P. cynomolgi* makes it a promising antimalarial lead drug.

## Materials and methods summary

Detailed information on materials and methods is available in the supplementary materials. In brief, the antimalarial activity of individual analogs was evaluated in vitro against *P. falciparum* 3D7 (MRA-102, drug sensitive), W2 (MRA-157, chloroquine resistant) strains (ATCC, Manassas, VA), Dd2 (MRA-156), NF54 (MRA-1000), HB3 (MRA-155), and D10-ACP-GFP (MRA-568) (55, 82, 83). *P. knowlesi* assays were quantitatively measured by flow cytometry using SYBR Green I and Mitrotracker Deep Red, as previously described (84, 85), on a Beckman Coulter CytoFLEX. Nonhuman primate infections were required to generate *P. cynomolgi* M/B strain parasites for in vitro testing (82, 86).

### RNA-seq

Differential expression analysis was done by use of R package DESeq2 with an adjusted *P*-value cutoff of 0.05. Volcano plots were made using R package Enhanced Volcano or GraphPad Prism 9 (GraphPad Software, Inc.).

### LC-MS metabolomics: Lipids

LC-MS metabolomics analysis was performed on a Synapt G2-Si quadrupole time-of-flight mass spectrometer (Waters) coupled to an I-class UPLC system (Waters).

### LC-MS metabolomics: Polar metabolites

Targeted metabolomics of polar, primary metabolites was performed on a TQ-XS triple quadrupole mass spectrometer (Waters) coupled to an I-class UPLC system (Waters).

### Metabolomic data processing and analysis

Untargeted data processing (peak picking, alignment, deconvolution, integration, and spectral matching) was performed in Progenesis Qi software (Nonlinear Dynamics). Data were normalized to total ion count. Similar metabolites features were assigned a cluster ID using RAMClust (41). An extension of the metabolomics standard initiative guidelines was used to assign annotation level confidence (87, 88). Targeted data processing was performed in Skyline software (89).

### P. falciparum PfSec13-mut *strains*

Gene editing of the *P. falciparum Sec13* gene (PF3D7_1230700) was performed using a two-plasmid–based strategy (2, 90, 91). The pCasG-Cas9-sgRNA plasmid vector plasmid contains the site to express the single-guide RNA (sgRNA), along with the yDHODH gene as the positive selection marker. The sgRNA was selected from the database generated by Desai and Ribeiro *et al.* (90, 92, 93).

### TPP mass spectrometry data

TPP mass spectrometry data were analyzed using mineCETSA R-language package as described previously (48, 49).

### Proteomics data processing and analysis

MS/MS spectra were interpreted using ProluCID v.1.3.3 (94). DTASelect v.1.9 (95) and swallow v.0.0.1, an in-house developed software (96), were used to control false discover rates at <1.2%. All datasets were contrasted against their merged dataset, respectively, using Contrast v1.9 (95) and in-house developed sandmartin. An in-house developed software, NSAF7 v.0.0.1, was used to generate spectral count–based label free quantitation results (40). QSPEC/QPROT (39, 42) was used to calculate log_2_(FC) and *z*-statistics (40, 82, 83, 86, 92, 97, 98).

### Yeast strain Sec13p expression analysis

Overexpression of *S. cerevisiae* Sec13p, was generated by an episomal *E. coli*/yeast shuttle vector, transformed into the BY4741 yeast strain. *SEC13* gene repression used the tet-off system.

### Tolerability studies

Tolerability studies used Male CD1 (20-22 g Envigo).

### Toxicological profiles

Blood samples for analysis were carried out in the Abacus5 junior Vet (Practice CVM S.L.L). Clinical chemistry was performed by mean analysis of whole blood in Vetscan (ABAXIS) and F560 (Menarini) analyzer and by mean analysis of plasma.

### In vivo efficacy studies

MED6-189 oral efficacy was tested in female NSG mice engrafted with human erythrocytes and then infected with *P. falciparum*. Peripheral blood from *P. falciparum*–infected mice was stained with TER-119-phycoerythrin (marker of murine erythrocytes) and SYTO-16 (nucleic acid dye) and then analyzed by flow cytometry (FACSCalibur, BD).

### Bioanalysis and pharmacokinetics analysis: LC-MS analysis

An Acquity ultra-performance liquid chromatography (UPLC) system (Waters Corp., Milford, MA, USA) couple to a triple quadrupole mass spectrometer (API 4000, AB Sciex, Foster City, CA, USA) was used for the analysis (99).

### Pharmacokinetic analysis

Blood concentration time data were analyzed by Non-Compartmental PK analysis using Phoenix WinNonlin software (Certara, NY, US) to calculate PK parameters.

### Statistical analyses

Parasitemia and proportion of asexual stages were analyzed using a two-way ANOVA with Tukey test for multiple comparisons. Significant differences were indicated as following: *P < 0.05; **P < 0.01, ***P < 0.001, and ****P < 0.0001. Statistical tests were performed with GraphPad Prism. Figures were generated with GraphPad Prism 9, Biorender, Adobe Illustrator v27.3.1. Putative Sec13 protein was created by Alphafold monomer V2.0 prediction for protein transport protein SEC13 (*Q8I5B3*) with PDB reference *AF-Q8I5B3-F1-model_v4* (*1*).pdb, and protein structure was formed through ChimeraX.

## Supplementary Material

Supp Meterial

Supp Tables

MDAR Reproducibility Checklist

## Data Availability

The MED6-189 purifications and TPP mass spectrometry datasets have been deposited to ProteomeXChange (under accession nos. PXD038457 and PXD038053, respectively) via the MassIVE repository (MSV000090812 and MSV000090667) and may also be accessed through the Stowers Original Data Repository (https://www.stowers.org/research/-publications/libpb-1759) (100, 101). WGS and RNA-seq datasets generated in this study have been deposited in the NCBI BioProject database under submission ID SUB12241156 with BioProject ID PRJNA930408 (102). Metabolomics datasets generated in this study are available in the Panorama database (103). The entire in-house software suite (Kite) used for the MudPIT mass spectrometry analysis is available in Zenodo (96).
