# Supplementary material for: A kalihinol analog disrupts apicoplast function and vesicular trafficking in P. falciparum malaria
Source: Science. Author manuscript; Available in PMC 2025 Sep 27. (PMC11793105; doi:10.1126/science.adm7966)
Supplement: Supp Meterial [file NIHMS2043742-supplement-Supp_Meterial.pdf]

Science
AAAS

Supplementary Materials for

A kalihinol analog disrupts apicoplast function and vesicular trafficking in *P. falciparum* malaria

Z. Chahine *et al*.

Corresponding authors: C. Ben Mamoun, choukri.benmamoun@yale.edu; C. D. Vanderwal, cdv@uci.edu; K. G. Le Roch, karine.leroch@ucr.edu

Science **385**, eadm7966 (2024)
DOI: 10.1126/science.adm7966

The PDF file includes:

Materials and Methods
Figs. S1 to S10
Supporting Information S1 and S2
References

Other Supplementary Material for this manuscript includes the following:

Tables S1 to S10
MDAR Reproducibility Checklist

Materials and Methods

Asexual parasite Culture and maintenance-- *P. falciparum* 3D7 (MRA-102) parasites were propagated in 5% of human O^+ erythrocytes and 10 mL of RPMI-1640 medium containing 0.5% Albumax II (Invitrogen), 2 mM L-glutamine, 50 mg/L hypoxanthine, 25 mM HEPES, 0.225% $NaHCO_3$ and 10 mg/mL gentamycin. They were maintained at 37 °C and gassed with a sterile mixture of 5% O_2, 5% CO_2 and 90% N2. Parasite synchronization was achieved using two 5% D-sorbitol treatments 8 hours apart (52, 82-86).

P. knowlesi strain YH1 (provided courtesy of Manoj Duraisingh) parasites were routinely cultured in 4% rhesus erythrocytes (Yerkes primate research center) and 10 mL of RPMI-1640 medium containing 0.5% Albumax II (Invitrogen), 2 mM L-glutamine, 50 mg/L hypoxanthine, 25 mM HEPES, 0.225% $NaHCO_3$ and 10 mg/mL gentamycin. They were maintained at 37 °C and gassed with a sterile mixture of 1% O_2, 5% CO_2 and 94% N2. Parasite synchronization was achieved using two 5% D-sorbitol treatments, spaced one per re-invasion cycle, 24 hours apart (84-86).

Animal use Nonhuman primate infections were required to generate *P. cynomolgi* M/B strain parasites for *in vitro* testing because these parasites are not adapted to continuous culture. Infections were performed at the University of Georgia, an Association for Assessment and Accreditation of Laboratory Animal Care (AAALAC) accredited institution. The UGA Institutional Animal Care and Use Committee reviewed and approved all procedures including blood collections, infections with malaria parasites, clinical interventions, etc. During the experimental procedures, the animals were housed in compliance with Animal Welfare Act regulations as well as the Guide for the Care and Use of Laboratory Animals. This includes multiple enrichment activities provided daily, including physical manipulanda and environmental enrichment, and routine health checks performed by facility animal resources staff and veterinarians.

All animal care and use protocols involving Male CD1 (20-22g Envigo) and NOD-*scid IL-2Rγ*^{null} (NSG) studies adhered to the specific national and international guidelines in accordance with the current Spanish laws (RD 53/2013; order ECC/566/2015) and the corresponding European Directive (2010/63 EU). All experimental protocols and procedures are internally reviewed and agreed by the Tres Cantos GSK Ethical Committee before starting experimental work. For assays involving the use of mice, including in the presence of possible toxic effects such as >20%

reduction in weight, aggressive or unexpected animal behavior, animals are immediately euthanized with CO_2. If no toxic effects are observed, mice are euthanized with CO_2 at the end of the experiment.

***P. cynomolgi* M/B strain parasite generation and cryopreservation** Captive bred Japanese macaques (*Macaca fuscata*) were obtained from Oregon National Primate Research Center. These animals were infected with *P. cynomolgi* M/B strain blood stage parasites that were cryopreserved. After infection, parasitemia was monitored daily by thick and thin blood films as previously described in Joyner *et al.* 2019 (86). When parasitemia reached about 1-3% ring stages, blood was collected from the infected animal and cryopreserved in Glycerolyte 57 solution as previously described.

***P. cynomolgi* M/B strain Drug Assay** *P. cynomolgi* M/B strain parasites preserved in Glycerolyte 57 were thawed as previously described (86). After thawing, the parasitemia was enumerated and adjusted to approximately 1% rings by diluting with freshly isolated Japanese macaques RBCs. The 1% parasitemia mixture was then plated into a 96 well round bottom culture plate at 5% hematocrit. The culture medium was comprised of IMDM supplemented with GlutaMAX (Gibco #31980030) containing 30 mM HEPES (Sigma-Aldrich), 17 mM D-glucose (Fisher Chemical) and 440 μM hypoxanthine (Sigma-Aldrich) and 1% Albumax II (Gibco) and 20% (v/v) heat-inactivated rhesus or Japanese macaque serum. Med6-189 was then added to each well to obtain the needed final concentrations of drug. After plating the parasites were placed into a StemCell hypoxia chamber with a gas mixture of 95% N_2 and 5% CO_2. Cultures were then placed into a CellExpert Eppendorf Incubator and maintained at 37°C and samples harvested at the indicated time points. Culture media was replaced daily, and drug re-added as described above to ensure that fresh compound was always present. During the drug assay thin blood smears were made and stained with Giemsa to determine the phenotypic changes with the parasites after exposure to the compound. For determining the IC_{50}, the parasites were harvested at 60 hours after the initial drug dosing. To determine the parasitemia in each well, the wells were resuspended in 30 ul of 10 μg/ml of Hoechst 33342 and incubated at for 30 minutes at 37°C. The stained culture wells were pelleted by centrifugation and resuspended in PBS followed by analysis on a Novocyte Quanteon Flow Cytometer (84,85). The experiment was repeated twice, and each concentration was assessed in triplicate.

Data analysis The percentage of parasitized erythrocytes was determined by enumerating the number of Hoechst positive events out of the total erythrocytes after excluding non-single cells by forward and side scatter. The percent survival was calculated by averaging the parasitemia in the triplicates for each condition and then dividing by the parasitemia observed in the wells that did not receive drug treatment. The IC_{50} was then calculated using GraphPad Prism 9 (GraphPad Software, Inc.).

Localization of fluorescently labeled MED6-189 analogue 3D7, NF54 and D10-Acp-GFP parasite lines were washed in incomplete medium then incubated with 1 µM of JF549-labelled kalihinol analogue (MED6-131) for 30 min-2 hrs. at 37°C, were washed in incomplete medium and fixed onto coverslip with 4% paraformaldehyde for 20 min at RT under darkness. After 3-5 PBS washing, coverslips were mounted in Vectashield Antifade Mounting Medium with DAPI (H-1200). Images were acquired using Keyence BZ-X810 and processed with ImageJ program.

Parasitemia and asexual stage phenotype analyses To understand the phenotypic effects of MED6-189 on *P. falciparum*, 3D7 lines were synchronized, grown for one cycle post-synchronization, and treated with either the vehicle (DMSO) or MED6-189 at IC_{80} values for 48 hrs. Culture media were replaced daily with reintroduction of the drug. Parasitemia and proportion of the different asexual blood stages were determined by counting Giemsa-stained blood smears by light microscopy at every 12-hour time point.

Parasitemia and sexual stage phenotype analyses To understand the phenotypic effects of MED6-189 on *P. falciparum* sexual stage development, NF54 lines were synchronized, and exposed to stress induced gametocyte induction followed by treatment with *N*-acetyl glucosamine (NAG; Sigma-Aldrich) supplemented media for 6-7 days to eliminate remaining asexual parasites. Upon first signs of gametocyte formation observed through giemsa stained smears (5-7 post stress induction), samples were treated with either the vehicle (DMSO) or MED6-189 at 3x IC_{80} values with 3 biological replicates per condition. Culture media were replaced daily with reintroduction of the drug. Gametocyte parasitemia and proportion of the different sexual blood stages were determined by counting Giemsa-stained blood smears by light microscopy at every 24-hour time point.

***P. falciparum in vitro* SYBR green fluorescence-based assays** The anti-malarial activity of individual analogues were evaluated *in-vitro* against *P. falciparum* 3D7 (MRA-102, drug sensitive) and W2 (MRA-157, Chloroquine resistant) strains (ATCC® Manassas, VA), Dd2 (MRA-156), NF54 (MRA-1000), HB3 (MRA-155), and D10-Acp-GFP (MRA-568) via SYBR green-I based fluorescence assay. Parasite growth rate and stages of development were determined via microscopy through Giemsa-stained smears of the cultures. Serial dilutions of the compounds ranging from 10uM-0.5nM were prepared in 96-well plates (Corning, Costar 3904) followed by the introduction of synchronized ring stage parasites cultures at 0.5% parasitemia and 2.5% hematocrit added to each well. Eight wells were treated with vehicle control (DMSO) as control. Plates were incubated in a modular incubation chamber (Billups-Rothenberg, Del Mar, CA) maintained at 37 °C for 72 hrs. in a low oxygen environment (96% N_2, 3% CO_2, 1% O_2). After 72 h, plates were removed from incubation and stored at -80˚C for 24 hrs. Plates were subsequently thawed followed by incubation with 1X volume of lytic buffer (20 mM Tris-HCl, pH 7.5, 5mM EDTA, 0.008% Saponin, 0.08% Triton X-100) containing SYBR Green 1X for 4–6 h at 37˚C in darkness. Plates were read with a Molecular Devices SpectraMAX Gemini EM at Ex. 495 nm, Em. 525 nm. Assessment of anti-malarial activity of compounds was made on the basis of fifty percent inhibitory concentration values (IC_{50}) determined by DNA content of the parasite using SigmaPlot (10 (Systat)) or GraphPad Prism v 9.0.0 under program Sigmoidal, 4PL, X is concentration, nonlinear regression, CI:95%) (82-83).

***P. knowlesi In vitro* IC_{50} Assays** Parasite lines were double synchronized with 5% D-sorbitol, grown for one cycle post-synchronization, and treated with a range of concentrations of either the vehicle (DMSO) or MED6-189, plated in triplicate. *P. knowlesi* parasites were allowed to re-invade (24 hours post-assay) and assays were harvested at 36 hours post-reinvasion. Parasitemia was quantitatively measured by flow cytometry using SYBR Green I, and Mitrotracker Deep Red as previously described (84-85) on a Beckman Coulter CytoFLEX (fig. S10). Data from 100,000 erythrocytes were acquired per sample and the percentage of double positive SYBR Green I positive, viable parasitized cells was calculated as parasitemia.

RNA-sequencing Two independent biological replicates were generated for each time point at the ring, trophozoite or schizont stage, for every culture condition and line. Total RNA was extracted with TRIzol® LS Reagent (Invitrogen) followed by incubation for 1 hr. with 4 units of DNase I

(NEB)at 37°C. RNA samples were visualized by RNA electrophoresis and quantified on Synergy™ HT (BioTek). mRNAs were then purified using NEBNext® Poly(A) mRNA Magnetic Isolation Module (NEB) according to the manufacturer's instructions. Libraries were prepared using NEBNext® Ultra™ Directional RNA Library Prep Kit (NEB) and amplified by PCR with KAPA HiFi HotStart Ready Mix (KAPA Biosystems). PCR conditions consisted of 15 min at 37°C followed by 12 cycles of [98°C (30 s), 55°C (10 s) and (62°C 1 min 15)] and 5 min at 62°C. The quantity and quality of the final libraries were assessed using a Bioanalyzer (Agilent Technology Inc). Libraries were sequenced using a NovaSeq 6000 DNA sequencer (Illumina), producing paired-end 100-bp reads. FastQC (https://www.bioinformatics.babraham.ac.uk/projects/fastqc/) was used to analyze raw read quality. Based on the quality data, the first 11 bp of each read and any adapter sequences were removed using Trimmomatic (http://www.usadellab.org/cms/?page=trimmomatic). Bases were trimmed from reads using Sickle with a Phred quality threshold of 25 (https://github.com/najoshi/sickle) and reads shorter than 18 bp were removed. The resulting reads were mapped against the *P. falciparum* 3D7 genome (v46) using HISAT2 (version 2-2.2.1) with default parameters. Uniquely mapped, properly paired reads with mapping quality of 40 or higher were retained using SAMtools (http://samtools.sourceforge.net/). PCR duplicates were removed using PicardTools MarkDuplicates (Broad Institute). Genome browser tracks were generated and viewed using the Integrative Genomic Viewer (IGV) (Broad Institute). Raw read counts were determined for each gene in the *P. falciparum* genome using BedTools (https://bedtools.readthedocs.io/en/latest/#) to intersect the aligned reads with the genome annotation. Differential expression analysis was done by use of R package DESeq2 to call up- and down-regulated genes with an adjusted P-value cutoff of 0.05. Volcano plots were made using R package Enhanced Volcano or GraphPad Prism 9 (GraphPad Software, Inc.).

Metabolomic profiling: Sample Preparation Tightly synchronized parasites (9×10^8 parasites at 72hrs post synchronization) were lysed with saponin, flash frozen and stored at −80 °C in triplicate. Lipids and polar metabolites were extracted from parasite pellets using a biphasic approach. To each sample, 1 mL of ice cold 3:2 methyl tert-butyl ether:80% methanol was added. To break up parasite pellets, samples were vortexed 2 min, sonicated for 15 min, vortexed for 2 min, sonicated for 15 min, then vortexed for 30 min at 4° C. All sonication was performed in an

ice bath. 200 µL of water was added to induce phase separation followed by a 5 min vortex. After centrifugation for 15 min at 4° C at 16,000 x g, 200 µL of the top, nonpolar layer was transferred to a 2 mL glass vial and the bottom, polar layer, was transferred to a new 2 mL glass vial then analyzed by LC-MS. The nonpolar fraction was dried under a gentle stream of nitrogen at room temperature then resuspended in 400 µL of 9:1 methanol: toluene and analyzed by LC-MS.

LC-MS Metabolomics-lipids LC-MS metabolomics analysis was performed on a Synapt G2-Si quadrupole time-of-flight mass spectrometer (Waters) coupled to an I-class UPLC system (Waters). Separations were carried out on a CSH phenyl-hexyl column (2.1 x 100 mm, 1.7 µM) (Waters). The mobile phases were (A) water with 0.1% formic acid and (B) acetonitrile with 0.1% formic acid. The flow rate was 250 µL/min, and the column was held at 40° C. The injection volume was 1 µL. The gradient was as follows: 0 min, 1% B; 1 min, 1% B; 8 min, 40% B; 24 min, 100% B; 26.5 min, 100% B; 27 min, 1% B. The MS was operated in positive ion mode (50 to 1200 m/z) with a 100 ms scan time. Source and desolvation temperatures were 150° C and 600° C, respectively. Desolvation gas was set to 1100 L/hr and cone gas to 150 L/hr. All gases were nitrogen except the collision gas, which was argon. Capillary voltage was 1 kV in positive ion mode. A quality control sample, generated by pooling equal aliquots of each sample, was analyzed every 3 injections to monitor system stability and performance. Samples were analyzed in random order. Leucine enkephalin was infused and used for mass correction.

LC-MS Metabolomics-polar metabolites Targeted metabolomics of polar, primary metabolites was performed on a TQ-XS triple quadrupole mass spectrometer (Waters) coupled to an I-class UPLC system (Waters). Separations were carried out on a ZIC-pHILIC column (2.1 x 150 mm, 5 µM) (EMD Millipore). The mobile phases were (A) water with 15 mM ammonium bicarbonate adjusted to pH 9.6 with ammonium hydroxide and (B) acetonitrile. The flow rate was 200 µL/min, and the column was held at 50° C. The injection volume was 2 µL. The gradient was as follows: 0 min, 90% B; 1.5 min, 90% B; 16 min, 20% B; 18 min, 20% B; 20 min, 90% B; 28 min, 90% B. The MS was operated in selected reaction monitoring mode. Source and desolvation temperatures were 150° C and 600° C, respectively. Desolvation gas was set to 1100 L/hr and cone gas to 150 L/hr. Collision gas was set to 0.15 mL/min. All gases were nitrogen except the collision gas, which was argon. Capillary voltage was 1 kV in positive ion mode and 2 kV in negative ion mode. System

stability was monitored by analyzing a quality control sample (generated by pooling together equal volumes of all sample extracts) every 3 injections. Samples were analyzed in random order.

Metabolomic data processing and analysis Untargeted data processing (peak picking, alignment, deconvolution, integration, and spectral matching) was performed in Progenesis Qi software (Nonlinear Dynamics). Data were normalized to total ion count. To group features belonging to the same metabolite, features were assigned a cluster ID using RAMClust (41). An extension of the metabolomics standard initiative guidelines was used to assign annotation level confidence (87-88). Several metabolite databases were screened including Metlin, Lipidblast, Mass Bank of North America, and an in-house database. Targeted data processing (manual peak integration) was performed in Skyline software (89).

Preparation of Thermal Proteome Profiling (TPP) samples for mass spectrometry analysis Acetone precipitated protein samples were resuspended in 100 µl of 100 mM triethylammonium bicarbonate (TEAB) and digested overnight at 37°C with 2.5 µg trypsin (Promega, Madison, WI). After digestion, approximately 0.5 *p*moles of LysC/trypsin digested porcine albumin was added to each sample as an internal control to monitor sample integrity. Peptide concentration was measured using the Pierce quantitative fluorometric peptide assay (Thermo Scientific, San Jose, CA). A sample volume equivalent to 30 µg in the lowest temperature sample (37°C) was used for subsequent labeling with TMT10plex reagents (Thermo Scientific, San Jose, CA). Samples were labeled with approximately 0.27 mg of TMT10plex reagent according to the manufacturer's instructions. Labels were matched to thermal challenge temperatures in order (TMT^{10}-126, 37°C to TMT^{10}-131, 73°C). Labeled samples for each treatment (DMSO, MED6-189) were pooled, buffer was removed using a vacuum centrifuge, and samples fractionated using the Pierce high pH fractionation kit according to the manufacturer's instructions. After fractionation, buffer was removed using a vacuum centrifuge and pellets resuspended in buffer A containing 95% HPLC grade water, 5% acetonitrile, and 0.1% formic acid (v/v/v), pH 2.6.

Mass spectrometry analysis of TPP samples TMT labeled samples were resolved for mass spectrometry analysis using a Dionex UltiMate 3000 RSLCnano liquid chromatography system. Peptides were first transferred from the autosampler to an Acclaim PepMap 100 C18 LC Trap

Cartridge (inside diameter 0.3 mm, length 5 mm). Peptides were then resolved using an analytical column (75 µm inside diameter, 250 mm length) packed with 1.9 µM ReproSil®-Pur C18-aQ resin. Chromatographic separations used different combinations of buffer A (95% HPLC grade water, 5% acetonitrile, and 0.1% formic acid (v/v/v), pH 2.6), and buffer B (20% HPLC grade water, 80% acetonitrile, and 0.1% formic acid (v/v/v), pH 2.6). Chromatography steps were performed with a flow rate of 180 nl/minute as follows: (1) 2% B for 35 minutes (column equilibration); (2) a linear gradient from 2% B to 10% B over 3 minutes; (3) a linear gradient from 10% B to 40% B over 40 minutes; (4) a linear gradient from 40% B to 80% B over 10 minutes; (5) 80% B for 10 minutes (column wash); (6) a linear gradient from 80 % B to 2% B over 0.1 minutes; (7) 2% B for 12 minutes (column re-equilibration).

Peptides were analyzed using an Orbitrap Eclipse Tribrid mass spectrometer (Thermo Scientific, San Jose, CA) fitted with a FAIMS Pro™ interface. FAIMS compensation voltages (CV values) of 40 V and 60 V were used. For peptide identification and quantitation, an MS3 method was used: Full MS scans were performed using the Orbitrap mass analyzer (120,000 m/z resolution, 400-1600 m/z scan range); Peptides were isolated using a window of 0.7 m/z for MS2 fragmentation (35% CID energy) and subsequent detection with the ion trap mass analyzer. Synchronous precursor selection was used to select up to 10 peptide fragments identified during the MS2 step for MS3 analysis; Peptides for MS3 analysis were isolated using an MS1/MS2 isolation windows of 2 m/z; TMT reporter ions were removed from peptide fragments using HCD (collision energy 55%) and detected using the Orbitrap mass analyzer (50,000 m/z resolution, 100-500 m/z scan range).

The resulting .raw files for two technical replicates of each condition were processed using Proteome Discoverer 2.5 (Thermo Scientific, San Jose, CA) using the common workflow templates "PWF_Tribid_TMT_Quan_SPS_MS3_sequestHT_Percolator" (processing step) and "CWF_Comprehensive_Enhanced Annotation_Reporter_Quan" (consensus step). The "Normalization Mode" of the "Reporter Ions Quantifier" node in the consensus step was set to "none". Data were searched using the database published by Dziekan *et al.*(49) with the addition of the sequence of porcine albumin.

Downstream Analysis of TPP mass spectrometry data Protein quantitation data were exported to .txt files (table S5) for analysis using the mineCETSA R-language package as described

previously (49, 52, 66). Exported files were pre-processed to retain only high-confidence *P. falciparum* proteins for which abundance ratios had been generated. The resulting TPP curves are provided as Supporting Information S2.

Immunoprecipitation and MudPIT mass spectrometry Mid- to- late-stage asexual parasites were collected following saponin treatment and purified. Samples were then resuspended in 50 mM–Tris-HCl pH 7.5, 300 mM NaCl, 0.5 mM EDTA, 0.5 mM EGTA, 2 mM AEBSF 0.5% Triton X-100, 20mM N-ethymaleimide and 1 mM AEBSF and EDTA-free protease inhibitor cocktail (99). Post cell lysis solution was homogenized via sonication for 6-9 rounds. The soluble extracts were centrifuged at 13,000g for 15 min at 4°C. The lysates were precleared with Dynabeads MyOne TM Streptavidin T1 (Invitrogen) for 1h at 4°C. 100uM of biotinylated kalihinol analogue (MED6-118) was added to each sample with the exception of DMSO added to a negative control and incubated for 1 hr at 4˚C. Following drug incubation Fresh Dynabeads MyOne TM Streptavidin T1 beads were added to each sample and incubated overnight at 4°C. Dynabeads were used to precipitate biotinylated drug-protein complexes and were washed with buffer (PBS, 0.05% Tween20). Proteins were eluted using 50mM Tris-HCl pH 6.7, 100mM DTT and 2% SDS. The eluent was subsequently precipitated on ice in 20% TCA overnight followed by cold acetone washes. The urea-denatured, reduced, alkylated, and digested proteins were analyzed by Multidimensional Protein Identification Technology (MudPIT) on a linear ion trap tandem mass spectrometer coupled to an Agilent 1100 series HPLC as described previously (98).

Proteomics data processing and analysis Tandem mass (MS/MS) spectra were interpreted using ProluCID v.1.3.3 (94) against a database consisting of 5527 non-redundant (NR) *Plasmodium falciparum* 3D7 proteins (PlasmoDB-42 release), 36661 NR human proteins (NCBI, 2018-03-30 release), 419 usual contaminants (human keratins, IgGs, and proteolytic enzymes). DTASelect v.1.9 (95) and swallow v.0.0.1, an in-house developed software (https://github.com/tzw-wen/kite) (96) were used to control FDRs at less than 1.2%. All datasets were contrasted against their merged data set, respectively, using Contrast v1.9 (95) and in-house developed sandmartin v.0.0.1 (https://github.com/tzw-wen/kite/tree/master/ kitelinux). Our in-house developed software, NSAF7 v.0.0.1 (https://github.com/ tzw-wen/kite/tree/master/windowsapp/NSAF7x64), was used to generate spectral count-based label free quantitation results (40). QSPEC/QPROT (40,42) was used to calculate $\log_2$ Fold-Changes and Z-statistics to compare five replicate MED6-189 affinity

purifications to three negative controls. Proteins detected in at least 2 of the 5 replicate analyses and not in the controls and/or with a log_2 FC ≥1.5 and Z-statistic ≥1.645 were considered significantly enriched in the MED6-189 affinity purifications.

Generation of transgenic *P. falciparum PfSec13-mut* strains Gene editing of the *P. falciparum Sec13* gene *(PF3D7_1230700)* spanning position (Ch12:1258922 - 1262608 (+)) on chromosome 12 was performed using a two-plasmid-based strategy. The plasmid pCasG-Cas9-sgRNA plasmid vector (gifted by the Prigge lab) contains the site to express the sgRNA, along with the yDHODH gene as the positive selection marker. The sgRNA was selected from the database generated by Desai *et al.* and cloned into pCasG-Cas9 plasmid vector at the BsaI restriction site (table S8A, B). The homology directed repair plasmid (modified pDC2-donor-*bsd* without eGFP) was designed to remove the 21 nt target region (Pf3D7_12_v3:1261089-1261110) with PCR insert of interest through homology arms using a BamHI restriction enzyme digest followed by overnight ligation for donor insertion. Selection was performed though selectable markers, blasticidin S-deaminase (bsd) (90) present in the pDC2-donor plasmid construct along with DSM-1, in which the resistance gene yDHODH is present in the pCASG plasmid. The 541bp Pf*Sec13* target specifying homology arm sequences were isolated through PCR amplification and gel purification using primer set A and B respectively amplified at 60°C and 54°C annealing temperatures respectively. The final donor vectors were confirmed by restriction digest and Sanger sequencing.

Plasmids were isolated from 250 mL cultures of *Escherichia coli* (XL10-Gold Ultracompetent Cells, Agilent Cat. 200314) and 60 µg of each plasmid was used to transfect ring stage parasites.

24-hrs before transfection, mature parasite cultures (6-8% parasitemia) were magnetically separated using magnetic columns (MACS LD columns, Miltenyi Biotec) and diluted to 1% parasitemia containing 0.5 mL fresh erythrocytes. The next day, ~3% ring stage parasites were pelleted and washed in 4 mL of cytomix (93). 200 µl of the infected erythrocytes were resuspended with the two plasmids in cytomix to a total volume of 400 µl in a 0.2 cm cuvette. Electroporation was performed with a single pulse at 0.310 kV and 950 µF using the Biorad GenePulser electroporator. Cells were immediately transferred to a flask containing 12 mL media and 400 µl erythrocytes. The culture medium was exchanged five hours post electroporation with 12 mL of fresh media. The following day, fresh culture media were added and supplemented with 1.5 µM DSM-1 (Calbiochem) and 2.5 µg/mL Blasticidin (RPI Corp B12150-0.1). Media and drug

selection was replenished every 24 hours for 7 consecutive days after which drug selection was halted and fresh media was replenished weekly. After 14 days, the culture was split into two flasks and 50 µl of erythrocytes were added every two weeks. Once parasites were detected by microscopy, integration of the insert was confirmed by gDNA extraction, PCR amplification of fragment regions upstream of 5' arm and downstream of 3'arm found in plasmid construct using primer sets indicated in (table S8B) and through genomic sequencing.

Isolation of *PfSec13-mut* Clones To generate a genetically homogenous parasite lines, the transfected parasites were serially diluted to approximately 0.5 parasite/well into 96 well plates. 200µl final volume of cultured parasites were incubated with Blasticidin S for 4 weeks with a weekly change of media and addition of fresh red blood cells during the first 2 weeks followed by media changes every 2 days until parasite recovery is observed through microscopic analysis of Giemsa-stained blood smears.

Molecular analysis of the *PfSec13-mut* line Genomic DNA (gDNA) was extracted and purified using DNeasy Blood & Tissue kit (Qiagen) following instructions from the manufacturer. The diagnostic and genotyping PCR analysis was used to genotype the transfected lines using the primer pairs (Set A: Sec13-insert-fwd, Sec13-insert-rev; Set B:Sec13-pam-mut-fwd, Sec13-pam-mut-R). The PCR amplification was conducted using 2xKAPPA master mix for thirty cycles with an annealing temperature of 54°C and 60°C for Sec13 extension, respectively, and extension temperature of 57°C. The PCR amplicons were analyzed by gel electrophoresis and sequenced. For whole genome sequencing, genomic DNAs were fragmented using a Covaris S220 ultrasonicator and libraries were generated using KAPA LTP Library Preparation Kit (Roche, KK8230). To verify that the insertion was present in the genome at the correct location in both transfected lines, reads were mapped using Bowtie2 (version 2.4.4) to the *P. falciparum* 3D7 reference genome (v48, PlasmoDB), edited to include the insertion sequence in the intended location. IGV (Broad Institute) was used to verify that reads aligned to the insertion sequence.

Analysis of MED6-189 effect on the growth of yeast strains with repressed or over-expressed Sec13p For overexpression of *S. cerevisiae* Sec13p, a DNA fragment consisting of 860 bp upstream, 894 bp *SEC13* coding sequence, and 297 bp 3' untranslated sequence was generated by

PCR amplification and introduced into an episomal *E. coli*/yeast shuttle vector, pRS425, by using an in-fusion cloning kit (Takara Bio USA, Inc) and the resulting pRS425-SEC13 plasmid was transformed into the BY4741 yeast strain. To swiftly repress the *SEC13* gene, we utilized the tet-off system. This involved substituting the native *SEC13* gene promoter with a tetracycline (tet)-regulatable counterpart, while leaving the original *SEC13* open reading frame intact. The yeast strain with the *SEC13* gene under the control of Tet off promoter (BY4741 kanR-tetO7-TATA-SEC13, URA::CMV-tTA) was obtained from Horizon Discovery Ltd and the repression of the gene on the strain was achieved by the addition of doxycycline to the yeast culture. To determine the effect of MED6-189 on the growth of the yeast strains, the cells were grown in a synthetic minimal growth medium to mid log phase, diluted and inoculated in 96-well plates at the cell destiny of 10^3 cells/mL in 100 µL culture volume also containing MED6-189 (12 sequential 2-fold dilutions at a range of 0 to 400 µM). Cell growth was monitored every 12 hours by measuring optical density at 600 nm using a BioTek SynergyMx microplate reader. Prizm software was used to analyze the data.

Variant analysis by genome-wide sequencing To call variants (SNPs/indels) in the transfected lines compared to a previously sequenced control 3D7 line, genomic DNA reads were first trimmed to adapters and aligned to the *Homo sapiens* genome (assembly GRCh38) to remove human-mapped reads. Remaining reads were aligned to the *P. falciparum* 3D7 genome using bwa (version 0.7.17) and PCR duplicates were removed using PicardTools (Broad Institute). GATK HaplotypeCaller (https://gatk.broadinstitute.org/hc/en-us) was used to call variants between the sample and the 3D7 reference genome for both the transfected lines and the 3D7 control. Only variants that were present in both transfected lines but not the 3D7 control line were kept. We examined only coding-region variants and removed those that were synonymous variants or were located in *var*, *rifin*, or *stevor* genes. Quality control of variants was done by hard filtering using GATK guidelines.

***In vitro* safety profile** The Enhanced Cross Screen Panel (X-Screen, eXP) consists of *in vitro* assays designed to measure the effect of test compounds on receptor sites, ion channels, transporters, and enzymes, and in phenotypic assays. These assays are generally run for small-molecule compounds (NCEs) to help define selectivity and/or to identify potential safety concerns.

These assays can also aid mode of action identification of an *in vivo* effect produced by a test agent.

Tolerability study Male CD1 (20-22g Envigo) were used in tolerability studies.
All animal studies were ethically reviewed and carried out in accordance with European Directive 2010/63/EEC and the GSK Policy on the Care, Welfare and Treatment of Animals. The animals were housed in cages (Tecniplast®). The cages contained autoclaved dust free corncob bedding (Rehofix MK2000, 1.7-2.2 mm particle size) and carry filters covers. Animal maintenance standard g-irradiated standard rodent diet (Envigo Teklad 2914) and ultra-filtered water was provided ad libitum. Environmental conditions: Photoperiods, simulated dawn/dusk facility, were adjusted to 12h of light (300 lux) and 12h darkness daily. The temperature range was 22± 2ºC. Relative humidity was 55% ± 10 and the air room was changed 15-20 times per hour. Environmental enrichment included nesting material (nesting cups, LBS), a red-tinted igloo (Datesand), a hanging clear tunnel (Datesand, and two wood blocks (Datensand).

Protocol design Male CD1 mouse (n=1) was dosed with an initial dose at 100 mg/kg (1-day, oral route, once a day, 10ml/kg). The animals were observed during the first four hours after administration and after 24h. The neurobehavioral assessment Functional Observational Battery (FOB) was performed. Adverse effects were found at 100 m/kg and new dose was selected used the modified symptom-based Up-&-Down method and adjusted a constant factor (1.6). Other male CD1 mouse was dosed at 62.5mg/kg and showed adverse effects. Two mice were treated at 50 mg/kg and at this dose didn´t show adverse effects. To confirm the data, 3 animals per dose (100mg/kg and 63.4 mg/kg) and vehicle group with 1% Methylcellulose + 0.5% Tween (n=2) were dosed. Systemic exposure levels at different times were taken (0.5h, 2h, 4h, 8, 24, 48h) of treated animals (n=3 per group) and a toxicological profile was performed after 7 days of treatment (hematology, clinical chemistry, and necropsy). Additionally in the same animals with the same scheduling sampling, blood samples of 15 uL were taken from lateral tail vein and recovered in a vial with the same quantity of water, mix and freeze until bioanalytical quantification for the determination of blood level exposures and pharmacokinetic analysis.

Toxicological Profile The bodyweight of animals was recorded before dosage, 24 hours after and every day in animals kept for 7 days. Any sign of toxicity was recorded for 4h and 24h hours after drug administration (Functional Observational Battery). All animals were sacrificed by CO_2 inhalation. Blood samples for analysis were then taken by intracardiac puncture and fractionated to blood recount and serum analysis. The blood recount was carried out in the Abacus5 junior Vet → (Practice CVM S.L.L). Clinical chemistry was performed by mean analysis of whole blood in Vetscan→ (ABAXIS) & F560 (Menarini→) analyzer and by mean analysis of plasma, obtained after centrifugation at 1800g rpm for 10 min in S-Microvette tubes (Sarstedt→) with Heparin-Lithium. The plasma parameters tested were: glucose, ALP, AST, ALT, GGT, LDH, AMY, CK Calcium, Phosphate, Sodium, Potassium, total bilirrubin, total protein, albumin, blood ureic nitrogen (BUN), creatinine, triglycerides, cholesterol. After necropsy, several organs (liver, kidney, spleen, Caecum, and stomach) were removed and weighed. Liver, kidney, and spleen were fixed in formalin (formaldehyde 10%) to further histopathology study (Hematoxilin/Eosin stain).

***In vivo* Efficacy** MED6-189 oral efficacy was evaluated using a standard 4-day test in female NOD-*scid IL-2Rγ*^{null} (NSG) mice engrafted with human erythrocytes and infected with 20×10^6 *Plasmodium falciparum*-infected erythrocytes (*P. falciparum Pf*3D7$^{0087/N9}$). The experiment was conducted by GlaxoSmithKline (Tres Cantos) and was ethically reviewed and carried out in accordance with European Directive 2010/63/EEC and the GSK Policy on the Care, Welfare and Treatment of Animals. The mice were obtained from Charles River Labs, bred specifically for research and certified pathogen free.

The human biological samples were sourced ethically, and their research use was in accord with the terms of the informed consent under an IRB/EC approved protocol. Erythrocyte concentrates from malaria-negative donors were provided by *Biobancos de Castilla y Leon and Centro de Transfusiones de Madrid, Spain.* Research was conducted according to POL-GSKF-410 and was in accord with the terms of the informed consent of each donor.

NSG mice were engrafted daily with human red blood cells intravenously injected in a final volume of 0.7 ml (50% hematocrit). Mice were engrafted during two weeks prior to *P. falciparum* infection, mice achieve around 60% of human red blood cells in mice peripheral blood.

P. falciparum infections were performed by intravenous inoculation. All mice were randomly assigned to their corresponding treatment. MED6-189 was given by oral gavage at doses of 50

mg/kg per day for four consecutive days. Treatment started at day 3 and finished at day 6 after infection. In all cases, parasitemia was assessed in samples from peripheral blood obtained at days 3, 4, 5, 6, and 7 after infection. Fresh samples of peripheral blood from *P. falciparum*-infected mice were stained with TER-119-Phycoerythrine (marker of murine erythrocytes) and SYTO-16 (nucleic acid dye) and then analyzed by flow cytometry (FACSCalibur, BD). Efficacy was determined using flow cytometry data as the percentage of Parasitemia reduction compared with Vehicle-treated group. MED6-189 levels in blood after oral administration were measured in serial blood samples obtained during the 23 h period after the first dose in all mice. Peripheral blood samples (15 µL) were taken at frequent intervals, mixed with 30 µL of H_2O milli Q and immediately frozen on dry ice. The frozen samples were stored at −80 ˚C until analysis. Vehicle-treated mice experienced the same blood-sampling regimen (table S9, S10).

Bioanalysis and pharmacokinetics analysis: LC-MS

Bioanalysis of *in vivo* samples: Sample pretreatment Before the analysis 15ul of blood samples were defrost at ambient temperature and mixture with 200ul of ACN:MeOH (80:20). After protein precipitation samples were filtered by 0.45um filter plate (Multiscreen Solvinert 0.45um FTPE, Millipore). The 2ul TFA (Trifluoroacetic acid, sigmaAldrich) were added to each filtered sample in order to derivatize the compound to formamide analogue. This process occurs spontaneously.

LC-MS analysis An Acquity Ultra-Performance liquid chromatography (UPLC) system (Waters Corp., Milford, MA, USA) couple to a triple quadrupole mass spectrometer (API 4000™, AB Sciex, Foster City, CA, USA) was used for the analysis. The MS/MS transition was 311.340 / 203.100.

The chromatographic separation was carried out at 0.4ml/min in a Acquity UPLC™ BEH C18 column (50x2.1 mm i.d., 1.7 mm; Waters Corp.) at 40ºC with Acetonitrile (sigmaAldrich) and 0.1% formic acid as eluents.

Pharmacokinetic analysis Blood concentration time data were analyzed by Non-Compartmental PK analysis using Phoenix WinNonlin software (Certara NY, US) to calculate PK parameters. The maximum observed concentration (Cmax) and the time to reach it (Tmax), were determined. The area under concentration-time curve from dosing at time zero to the last measured concentration

time (AUClast) and the total area under concentration-time curve from time zero extrapolated to infinity (AUCinf), were calculated using a linear trapezoidal rule.

Supplemental Figures

Figure S1. Route for the synthesis of MED6-189 on gram scale (See Supporting Information S1).

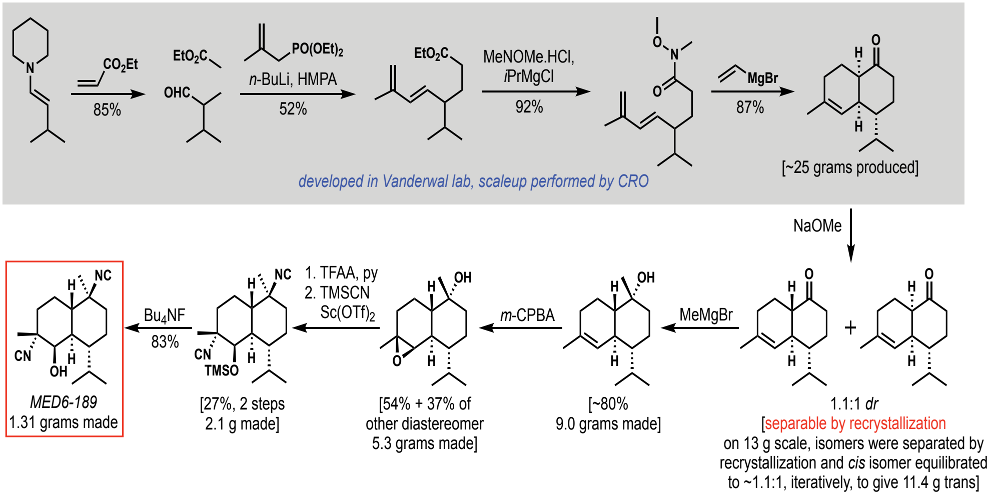

Figure S2A. Sexual Stage developmental impact of *P. falciparum* NF54 parasites incubated with MED6-189 at 3XIC80 concentration. **B**. Representative chemical profile of JF549-labelled kalihinol analogue. **C.** Representative chemical profile of the biotinylated kalihinol analogue.

A

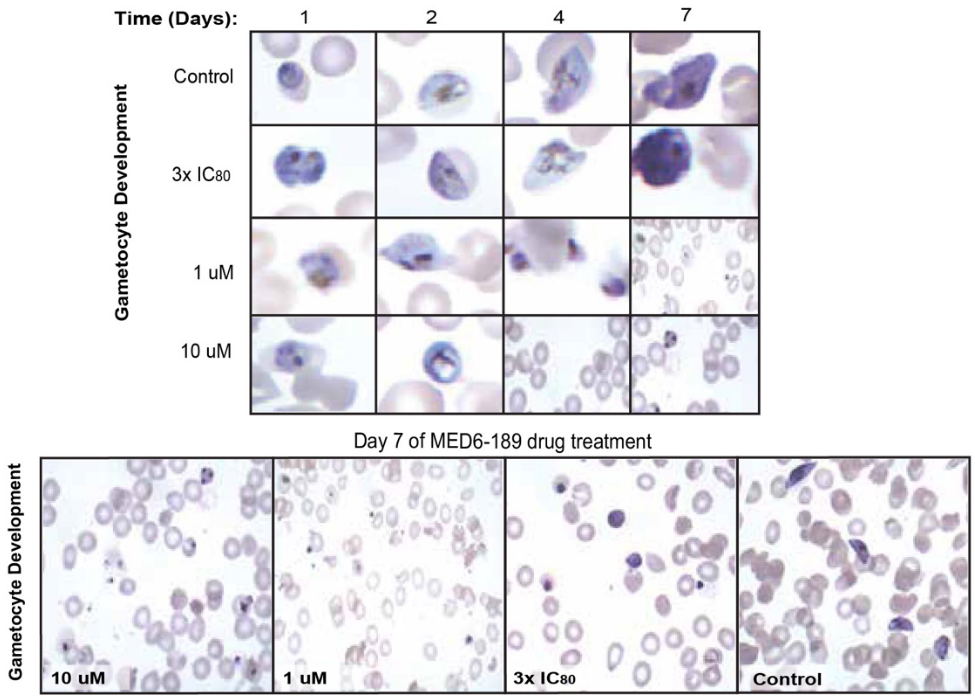

B

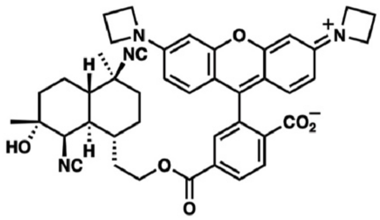

MED6-131

3D7 17 nM

C

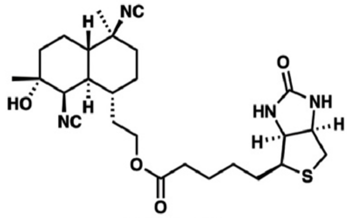

MED6-118

3D7 92 nM

Figure S3. Gene ontology bar graphs representing parasite transcriptomic responses to MED6-189. Gene ontology bar graph representing the top downregulated (top) and upregulated (bottom) transcripts from parasite samples cultured with either DMSO or MED6-189 collected at 72 HPS. Represented transcripts are filtered by highest number and percentage among most significantly expressed GO terms (Adjusted p-value ≤0.05).

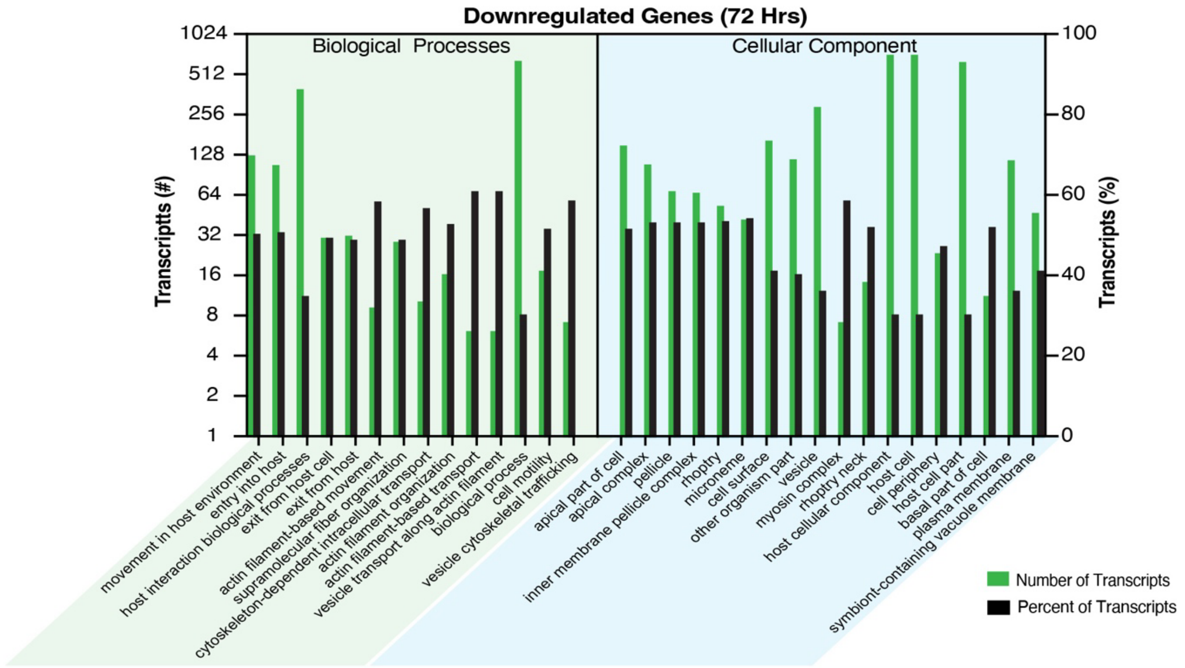

Downregulated Genes (72 Hrs)
Biological Processes
Cellular Component
Transcriptts (#)
Transcripts (%)
1024
512
256
128
64
32
16
8
4
2
1
100
80
60
40
20
0
movement in host environment
entry into host
host interaction biological processes
exit from host cell
exit from host
actin filament-based movement
supramolecular fiber organization
cytoskeleton-dependent intracellular transport
actin filament organization
actin filament-based transport
vesicle transport along actin filament
biological process
cell motility
vesicle cytoskeletal trafficking
apical part of cell
apical complex
pellicle
inner membrane pellicle complex
rhoptry
microneme
cell surface
other organism part
vesicle
myosin complex
rhoptry neck
host cellular component
host cell
cell periphery
host cell part
basal part of cell
plasma membrane
symbiont-containing vacuole membrane
Number of Transcripts
Percent of Transcripts

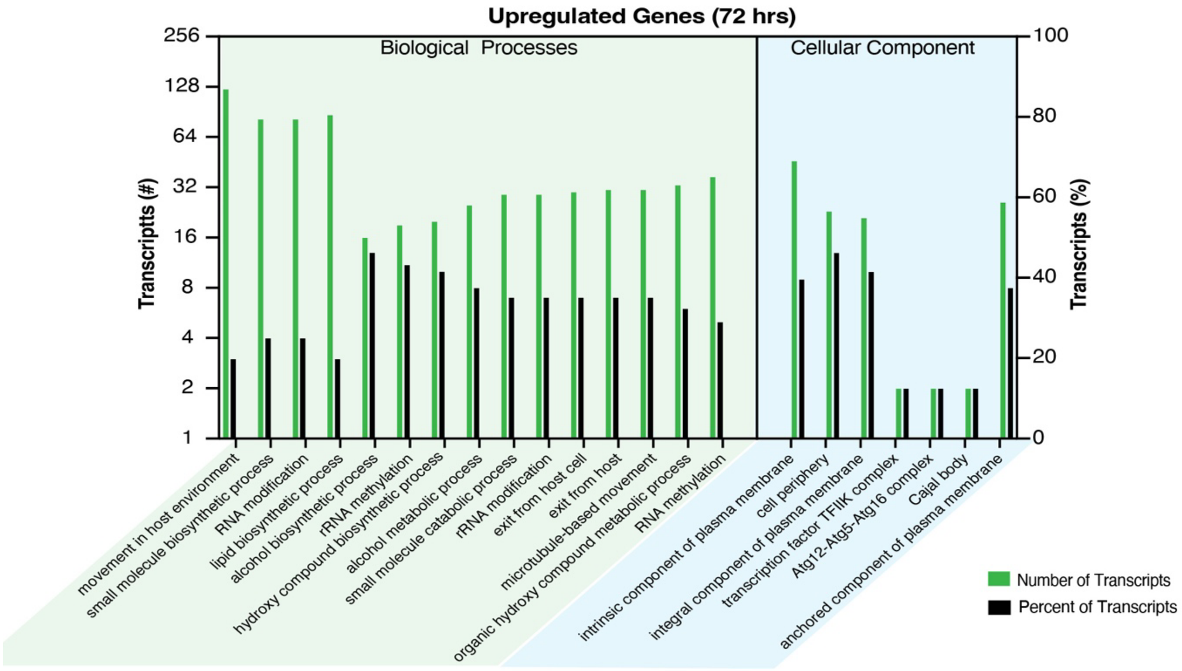
Upregulated Genes (72 hrs)
Biological Processes
Cellular Component
Transcriptts (#)
Transcripts (%)
256
128
64
32
16
8
4
2
1
100
80
60
40
20
0
movement in host environment
small molecule biosynthetic process
RNA modification
lipid biosynthetic process
alcohol biosynthetic process
rRNA methylation
hydroxy compound biosynthetic process
alcohol metabolic process
small molecule catabolic process
rRNA modification
exit from host cell
exit from host
microtubule-based movement
organic hydroxy compound metabolic process
RNA methylation
intrinsic component of plasma membrane
cell periphery
integral component of plasma membrane
transcription factor TFIIK complex
Atg12-Atg5-Atg16 complex
Cajal body
anchored component of plasma membrane
Number of Transcripts
Percent of Transcripts

Figure S4. Heatmap visual representation of polar metabolites significantly upregulated (green) or downregulated (red) in response to MED6-189 treatment. Log_2 transformation was applied to the data to compute q-value (BH adjusted p-value) and p-value from Welch's t-test or ANOVA test.

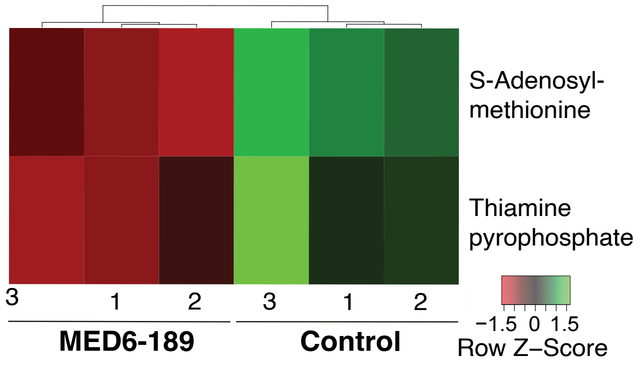

Figure S5. TPP melt-curve analysis of *P. falciparum* lysates treated with MED6-189. Protein stabilization curves for *P. falciparum* proteins identified in lysates treated with or without 100µM Med6-189 (MED6). Stabilization is assessed from the relative amount of soluble protein remaining (y axis for panels A-D) after thermal challenge (x axis for panels A-D). Replicate analyses are plotted in shades of red (MED6-189 treated) or blue (DMSO controls), while the pyrimethamine data from Dziekan et al 2020 is in shades of green (panel D). From the mineCETSA outputs (Table S6), differences in melting temperatures (ΔTms) between the Med6-189 and DMSO datapoints are reported on each plot when available (Tm values calculated in both replicates of DMSO and Med6-189 datasets). When ΔTms could not be calculated, a dashed arrow indicates trends (red - stabilization/ blue - destabilization). **A.** Proteins enriched in MED6-189 pull-down but not showing the distinct right-shifted stabilization profile in the presence of MED6-189. **B.** Proteins detected in MED6-189 affinity purification that did not pass significance cut-off but showed slight to marked stabilization to increasing temperature in the presence of MED6-189. **C.** Proteins showing significant changes in thermal denaturation in the presence of the drug but not detected in MED6-189 affinity purification. **D.** Thermal profiles of the Endoplasmin and SEC13 proteins whose genes are mutated in MED6-189 refractory parasites; Endoplasmin was not significantly enriched in MED6-189 pull-downs and does not show any stabilization to temperature denaturation in the presence of MED6-189; SEC13 was not detected in MED6-189 affinity purification yet showed no protein denaturation with increasing temperature in the presence of the drug compared to the melting curves obtained with pyrimethamine and DMSO controls from *Dziekan et al* 2020. **E.** Distribution of the melting temperatures (Tm) measured in this study compared to the ones observed in *Dziekan et al* for 337 proteins with Tm calculated with R2 >0.9 in both DMSO replicates of both studies (Table S6).

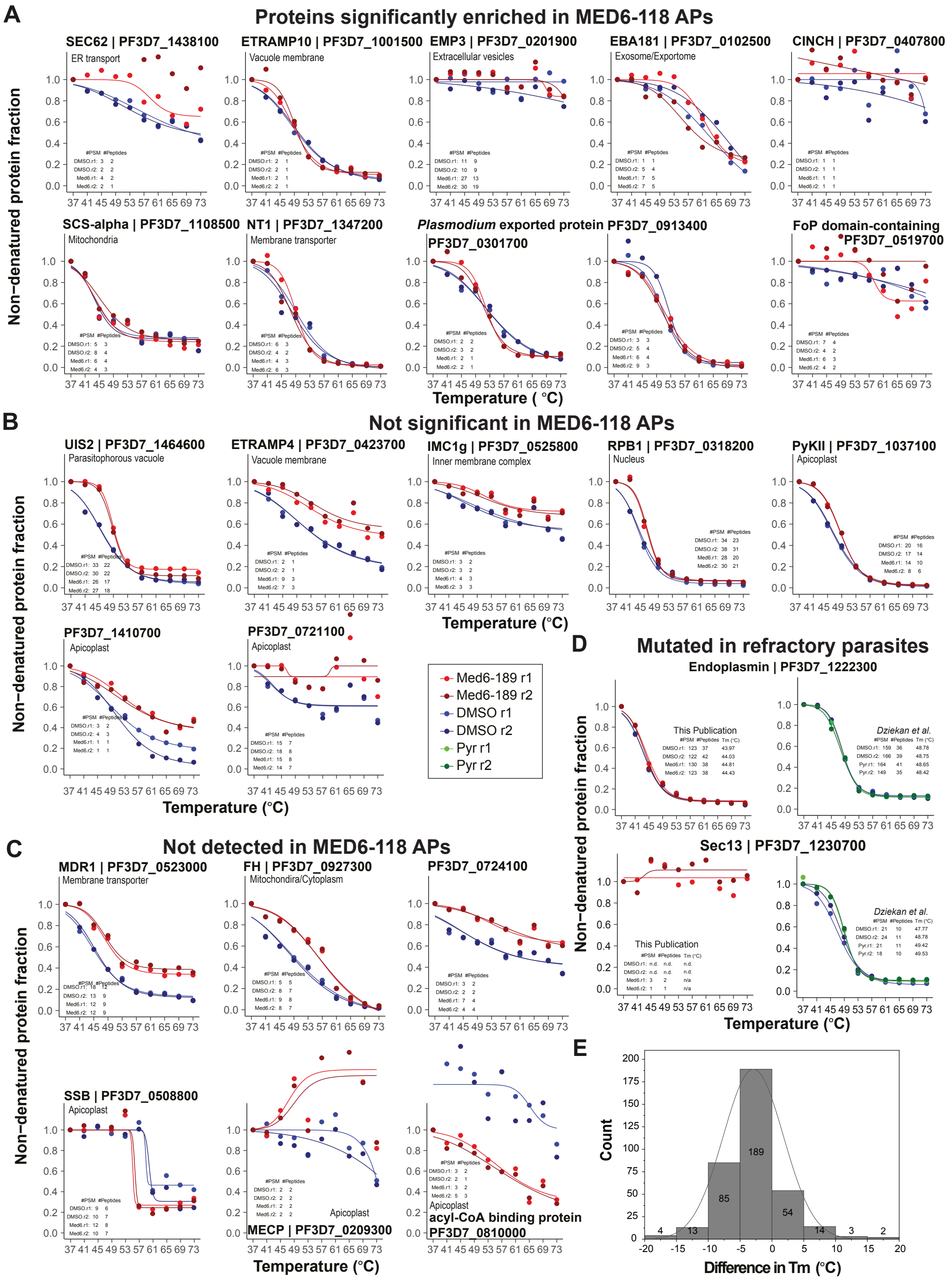
A
Proteins significantly enriched in MED6-118 APs
SEC62 | PF3D7_1438100
ER transport
ETRAMP10 | PF3D7_1001500
Vacuole membrane
EMP3 | PF3D7_0201900
Extracellular vesicles
EBA181 | PF3D7_0102500
Exosome/Exportome
CINCH | PF3D7_0407800
SCS-alpha | PF3D7_1108500
Mitochondria
NT1 | PF3D7_1347200
Membrane transporter
Plasmodium exported protein PF3D7_0913400
PF3D7_0301700
FoP domain-containing PF3D7_0519700
Non−denatured protein fraction
Temperature (°C)
B
Not significant in MED6-118 APs
UIS2 | PF3D7_1464600
Parasitophorous vacuole
ETRAMP4 | PF3D7_0423700
Vacuole membrane
IMC1g | PF3D7_0525800
Inner membrane complex
RPB1 | PF3D7_0318200
Nucleus
PyKII | PF3D7_1037100
Apicoplast
PF3D7_1410700
Apicoplast
PF3D7_0721100
Apicoplast
Med6-189 r1
Med6-189 r2
DMSO r1
DMSO r2
Pyr r1
Pyr r2
Temperature (°C)
C
Not detected in MED6-118 APs
MDR1 | PF3D7_0523000
Membrane transporter
FH | PF3D7_0927300
Mitochondria/Cytoplasm
PF3D7_0724100
SSB | PF3D7_0508800
Apicoplast
Apicoplast
MECP | PF3D7_0209300
Apicoplast
acyl-CoA binding protein
PF3D7_0810000
Temperature (°C)
D
Mutated in refractory parasites
Endoplasmin | PF3D7_1222300
This Publication
Dziekan et al.
Sec13 | PF3D7_1230700
Temperature (°C)
E
Count
Difference in Tm (°C)

Figure S6. TPP melting-curve analysis of *P. falciparum* lysates treated with pyrimethamine or DMSO from *Dziekan et al* 2020. **A-C.** Thermal profiles previously established by *Dziekan et al*, 2020 for the proteins plotted in Fig. S5A-C. **D-E.** Thermal profiles reported in *Dziekan et al 2020* for the proteins plotted in Fig 3 D-E.

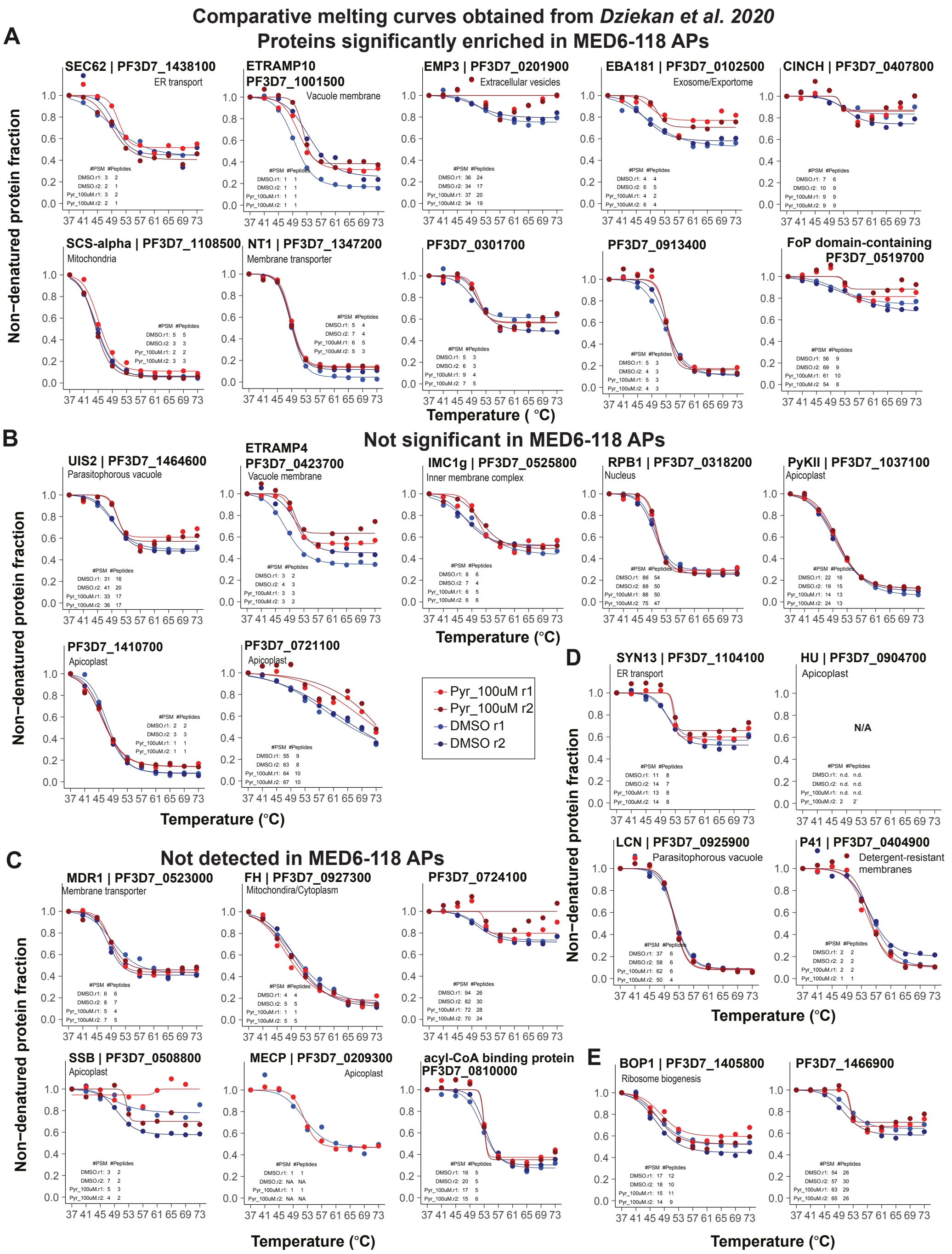
Comparative melting curves obtained from Dziekan et al. 2020
A
Proteins significantly enriched in MED6-118 APs
SEC62 | PF3D7_1438100
ER transport
ETRAMP10
PF3D7_1001500
Vacuole membrane
EMP3 | PF3D7_0201900
Extracellular vesicles
EBA181 | PF3D7_0102500
Exosome/Exportome
CINCH | PF3D7_0407800
SCS-alpha | PF3D7_1108500
Mitochondria
NT1 | PF3D7_1347200
Membrane transporter
PF3D7_0301700
PF3D7_0913400
FoP domain-containing
PF3D7_0519700
Non−denatured protein fraction
Temperature (°C)
B
Not significant in MED6-118 APs
UIS2 | PF3D7_1464600
Parasitophorous vacuole
ETRAMP4
PF3D7_0423700
Vacuole membrane
IMC1g | PF3D7_0525800
Inner membrane complex
RPB1 | PF3D7_0318200
Nucleus
PyKII | PF3D7_1037100
Apicoplast
PF3D7_1410700
Apicoplast
PF3D7_0721100
Apicoplast
Pyr_100uM r1
Pyr_100uM r2
DMSO r1
DMSO r2
Temperature (°C)
C
Not detected in MED6-118 APs
MDR1 | PF3D7_0523000
Membrane transporter
FH | PF3D7_0927300
Mitochondria/Cytoplasm
PF3D7_0724100
SSB | PF3D7_0508800
Apicoplast
MECP | PF3D7_0209300
Apicoplast
acyl-CoA binding protein
PF3D7_0810000
D
SYN13 | PF3D7_1104100
ER transport
HU | PF3D7_0904700
Apicoplast
N/A
LCN | PF3D7_0925900
Parasitophorous vacuole
P41 | PF3D7_0404900
Detergent-resistant membranes
E
BOP1 | PF3D7_1405800
Ribosome biogenesis
PF3D7_1466900

Figure S7. Enhanced Cross Screen Panel (eXP) graph. Pharmacological and toxicology values among compounds of interest

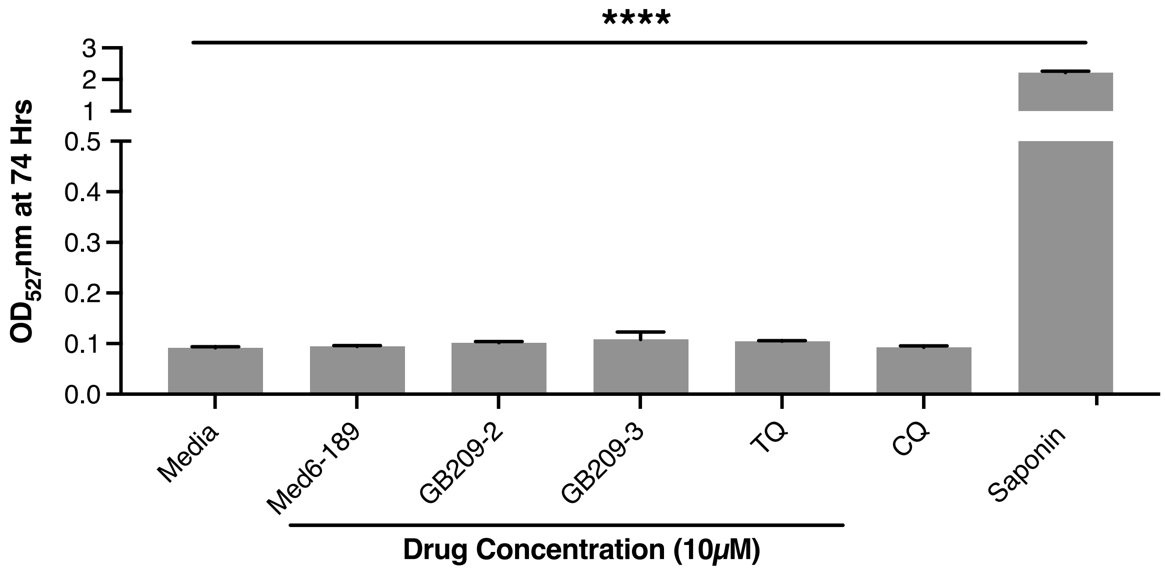

Figure S8. Giemsa smeared culture images depicting the hemolytic activity assays for MED6-189 and two formamide derivatives. No hepatoxicity was observed in response to MED6-189, GB209-2 and GB209-3 compounds tested after 48Hr and 72Hr drug incubation.

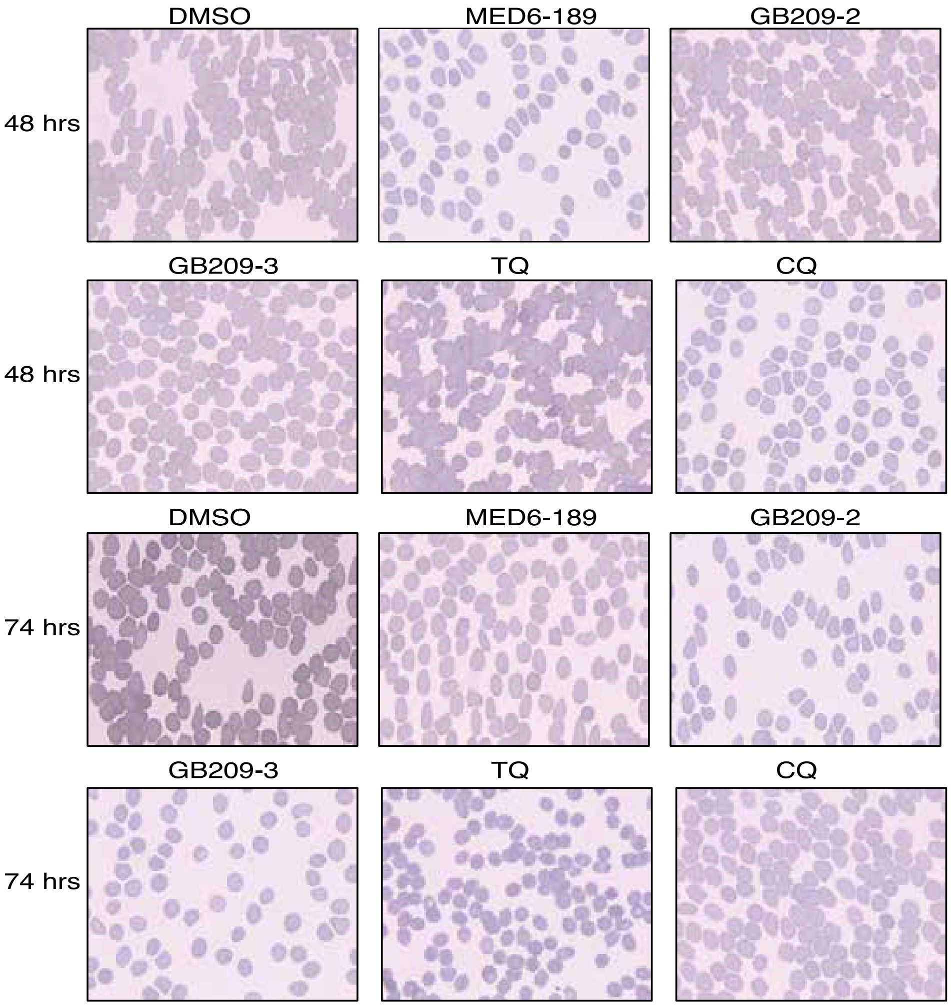

Figure S9. Flow cytometry panels illustrating results of SYBR green staining as well as Mitotracker Deep Red to determine viable parasites. Data for the IC_{50} plots are result of double positive staining (SYBR+, MTDR+) - representing viable parasites.

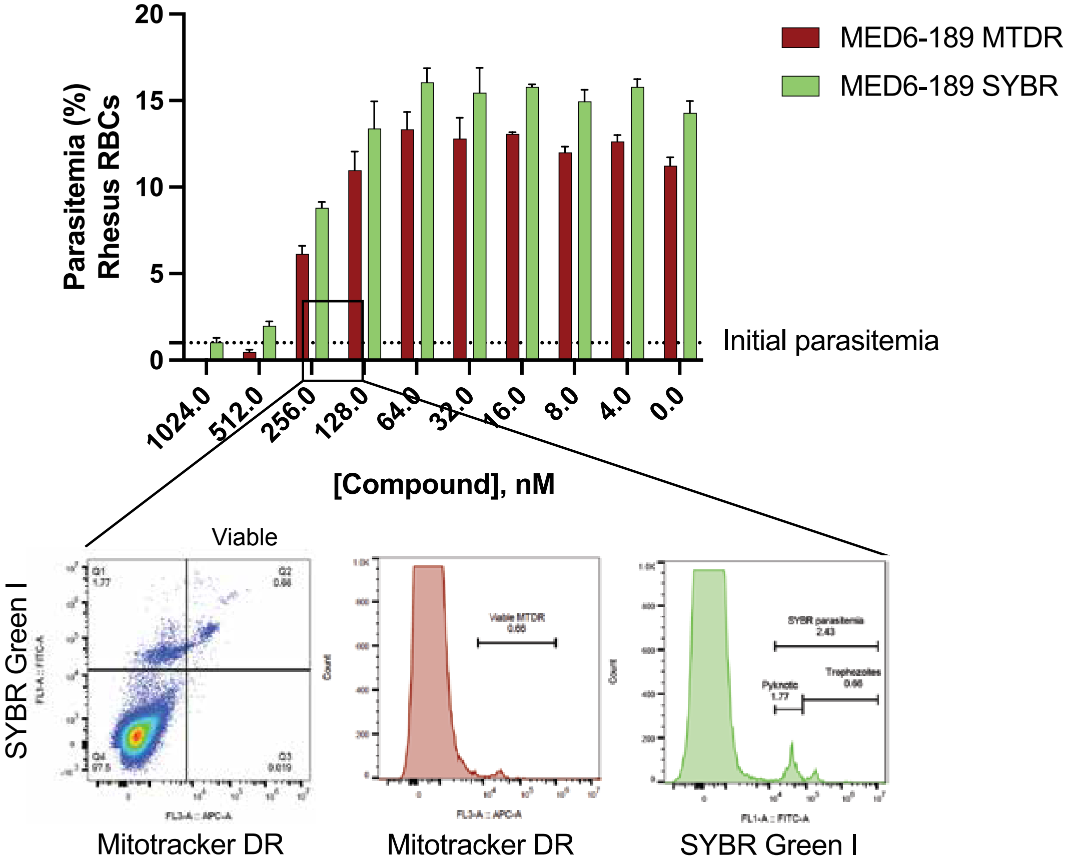

Figure S10 A. Dose-dependent response of MED6-189 on *P. cynomolgi* M/B strain parasites. The figures exhibit the logarithmic growth of parasites (Y-axis) corresponding to varying drug concentrations (X-axis). The error bars represent the standard deviation obtained from two independent experiments conducted in triplicate. The regression line represents a nonlinear regression analysis (Variable slope with four parameters, least squares fit). **B.** Giemsa-stained images of *P. cynomolgi* M/B strain parasites incubated with MED6-189 examined by light microscopy at different developmental stages of the parasite cell cycle.

A

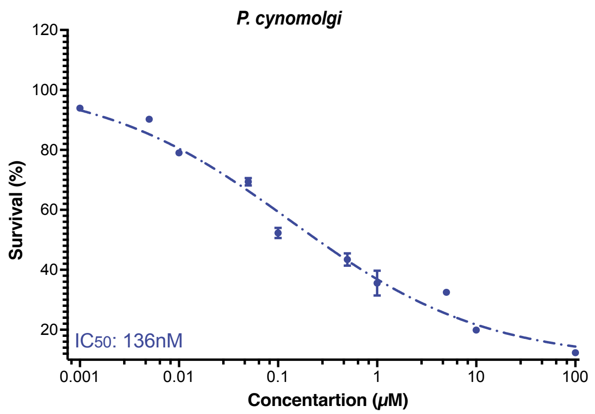

B

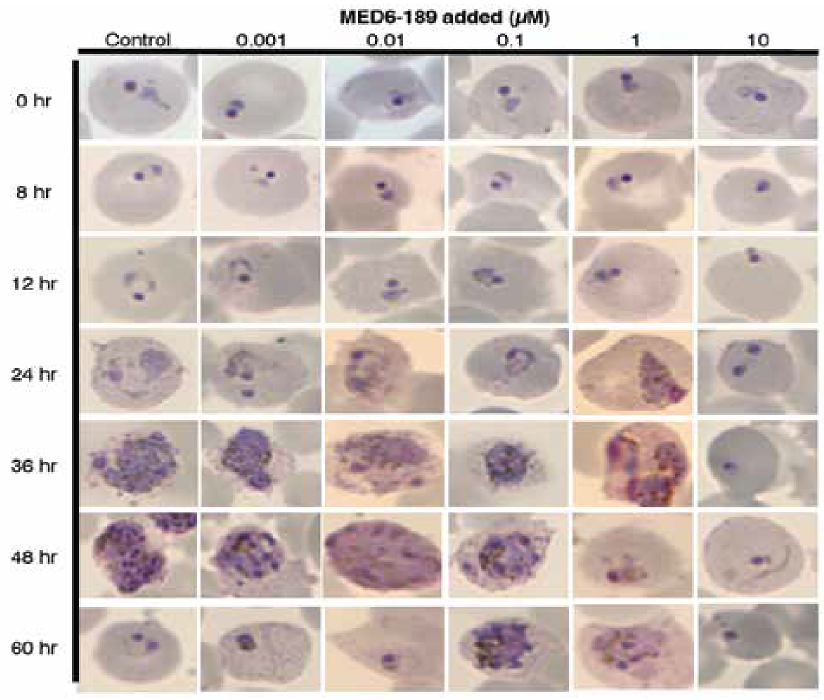

Supplemental Tables

Table S1A. MED6-189 SYBR green dose response assays. **B.** Sexual Stage developmental impact of *P. falciparum* NF54 parasites incubated with MED6-189 at 3X IC_{80}.

Table S2A. The Fractional Inhibitory concentration index (FIC) shown for drug ratio combination interactions on four tested compounds against MED6-189 at respective IC_{50} values. **B.** The Mean Inhibitory Concentration (MIC) scores respective of each compound is listed and used to calculate the ∑FIC. ∑FIC values refer to the kinetic order and shape of the respective curve where ∑FIC = 1, > 1, and < 1 indicates hyperbolic, sigmoidal, and negative sigmoidal shape, respectively. The *r* value is the linear correlation coefficient for the median-effect plot and indicates conformity to the curve. Differences in IC_{50} values between treatment groups were analyzed using GraphPad Prism 9 (GraphPad Software, Inc.) and differences considered significant if $P < 0.05$. Activity correlations between each compound and MED6-189 were analyzed by Pearson correlation (r) using GraphPad Prism 9 (GraphPad Software, Inc.). **C.** Exponential killing rate values of Fosmidomycin and MED6-189 treated *P. falciparum* cultures at respective IC_{80} values.

Table S3. Transcriptomic comparisons between MED6-189 treated and untreated samples at all tested time points. (Accession cite for data: BioProject database under SubmissionID: SUB12241156 with BioProject ID: PRJNA930408) **A.** DEseq2 analysis of drugged and control parasites. All genes for each time point (12, 24, 36, 48, 60 and 72 hours) are indicated**. B.** Significant genes obtained by DEseq2 analysis at 24, 60 and 72 hours for drugged and control parasites. No differentially expressed genes were identified at 12, 36 and 48 hours.

Table S4. Proteins Identified by Mass Spectrometry Analysis of MED6-118 Affinity Purifications and Negative Controls. (ftp://MSV000090812@massive.ucsd.edu) (100)

Table S5. *Plasmodium* Proteins Quantified by Mass Spectrometry Analysis of Thermal Proteome Profiling (TPP) in the Presence of MED6-189 or DMSO. (ftp://MSV000090667@massive.ucsd.edu)(101)

Table S6. Comparison of *Plasmodium* Proteins Identified by Thermal Proteome Profiling (TPP) in this study and by *Dziekan et al,* 2020.

Table S7. Whole genome sequencing results for MED6-189 drug pressured cultures. (BioProject database under SubmissionID: SUB12241156 with BioProject ID: PRJNA930408 (102))

Table S8A. Plasmid construct design and selectable markers present for generation of Sec13-mut lines. **B.** Primer sets, gRNA and gene sequence information used Sec13-mut plasmid constructs.

Table S9A. Activity of MED6-189 against *P. falciparum* strains and human cell lines and the resulting in vitro therapeutic index (TI). **B.** Pharmacokinetic profile of Kalihinol (GSK3943190A) at 25 and 50 (mg/Kg).

Table S10. Pharmacokinetics parameters calculated after oral administration (in 1% Methylcellulose) of 50 mg/kg to *P. falciparum*-infected SCID mice. Additionally blood levels at 96 hrs. has been measured and compound show no accumulation after four days of administration every 24h.

Supporting information S1

General experimental procedures:

S1. Supplemental Information for the synthesis of MED6-189 on gram scale (See Fig. S1).

Tetrahydrofuran (THF, Fisher, HPLC Grade) was dried by percolation through a column packed with neutral alumina and a column packed with Q5 reactant, a supported copper catalyst for scavenging oxygen, under a positive pressure of argon. Solvents for workup and chromatography include hexanes (Fisher, ACS Grade), ethyl acetate (EtOAc, Fisher, ACS Grade), dichloromethane (DCM, Fisher, ACS Grade), chloroform ($CHCl_3$, Fisher, ACS Grade), methanol (MeOH, Fisher, ACS grade) and were used as obtained commercially unless otherwise stated. Column chromatography was performed using EMD Millipore 60 Å (0.040–0.063 mm) silica gel (SiO_2). Analytical thin-layer chromatography was performed on Merck silica gel 60 F254 TLC plates. Visualization was accomplished with UV (254 or 210 nm), p-anisaldehyde, potassium permanganate, iodine/silica, and/or heat as developing agents. Chloroform-d ($CDCl_3$, D 99.8%, DLM-7) was purchased from Cambridge Isotope Laboratories. Methanol-d4 (D_3COD, 99.8%) was purchased from Sigma–Aldrich. Proton and carbon magnetic resonance spectra (^1H NMR and ^{13}C NMR) were recorded at 298K on a Bruker CRYO500 (500 MHz, ^1H; 125 MHz, ^{13}C) or a Bruker AVANCE600 (600 MHz, ^1H; 151 MHz, ^{13}C) spectrometer with solvent or residual solvent resonance as the internal standard (^1H NMR: [$CDCl_3$], $CHCl_3$ at 7.26 ppm, [D_3COD], HD_2COD at 3.31 ppm, ^{13}C NMR: $CDCl_3$ at 77.16 ppm). ^1H NMR data are reported as follows: chemical shift, multiplicity (s = singlet, d = doublet, t = triplet, q = quartet, dd = doublet of doublets, ddd = doublet of doublet of doublets, td = triplet of doublets, tdd = triplet of doublet of doublets, qd = quartet of doublets, m = multiplet, br s = broad singlet), coupling constants (Hz), and integration. Mass spectrometry data were obtained from the University of California, Irvine Mass Spectrometry Facility. High resolution mass spectra (HRMS) were recorded on a Waters LCT Premier spectrometer using ES+ and data are reported in the form of (m/z).

Synthetic preparation of MED6-189: Following modified procedures of Taber *et al* (1979), Shenvi *et al* (2013), and Vanderwal *et al* (2017), 1.31 g of racemic MED6-189 was prepared according to the route shown in figure S1 below.

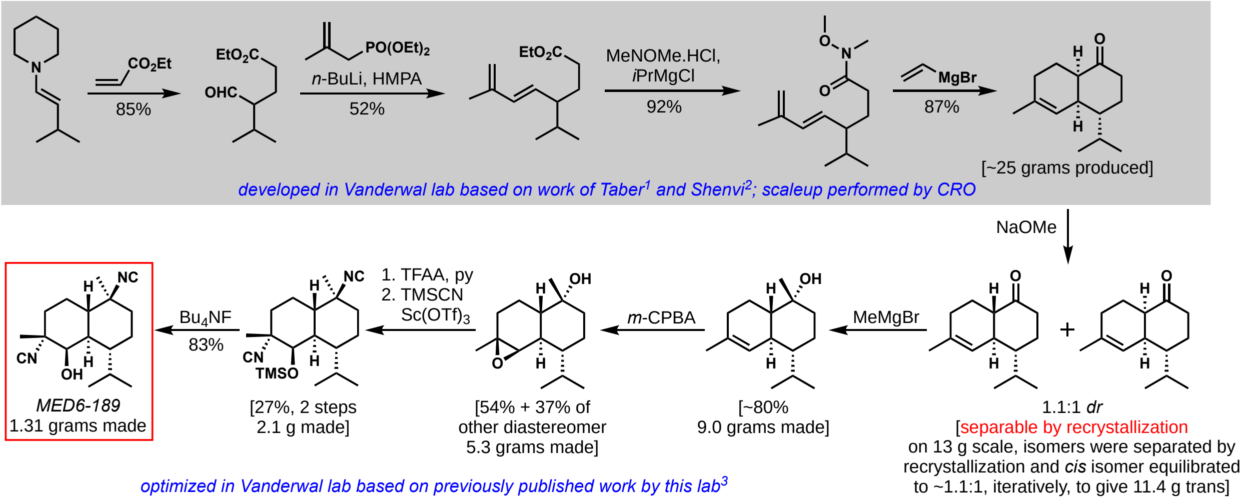

Fig. S1. Route for the synthesis of MED6-189 on gram scale.

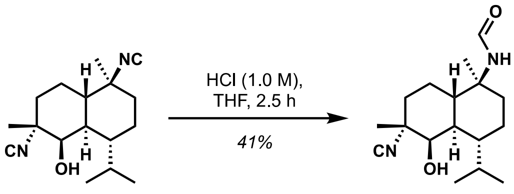

MED6-189 GB-209-2

Synthetic preparation of GB-209-2: To a 1-dram vial containing MED6-189 (4.8 mg, 17 µmol) and a magnetic stir bar was added THF (tetrahydrofuran, 1.7 mL) and hydrochloric acid (0.87 mL, 1.0 M, 870 µmol). The mixture was stirred at room temperature for 2.5 h. The reaction was quenched by the addition of sodium bicarbonate (saturated aqueous solution, 1.0 mL). The organic phase was extracted using CH_2Cl_2 (3 x 1 mL) and the combined extracts were dried using anhydrous magnesium sulfate. The volatiles were removed under reduced pressure in a rotary evaporator then under high vacuum to afford a colorless residue that was further purified using flash column chromatography (eluent: EtOAc:hexanes = 40:60, both distilled). GB-209-2 was obtained as a colorless oil that solidified upon standing (2.1 mg, 41% yield). The material will precipitate from DCM upon addition of hexanes to obtain the product in powdered form.

Note: GB-209-2 was observed as two different amide rotameric species (R^1, R^2 ratio = 55:45, respectively) under ambient conditions, complicating NMR spectroscopic analysis.

^{1}H NMR (500 MHz, $CDCl_3$, residual $CHCl_3$ at 7.26 ppm): δ 8.28 (R^1: [O=C**H**], d, *J* = 12.3 Hz, 1H), 8.05 (R^2: [O=C**H**], d, *J* = 2.1 Hz, 1H), 5.52 (R^1: [N**H**], d, *J* = 12.2 Hz, 1H), 5.09 (R^2: [N**H**], s, 1H), 3.81 (R^1: [HO–C**H**], apparent s, 1H), 3.79 (R^2: [HO–C**H**], apparent s, 1H), 2.32 (R^1: [HO–CH–C**H**], td, *J* = 12.1, 3.3 Hz, 1H), 2.26 (R^2: [HO–CH–C**H**], td, *J* = 12.1, 3.3 Hz, 1H), 2.0–1.92 (m, 1H), 1.91–1.79 (m, 2H), 1.78–1.60 (m, 5H), 1.54–1.40 (m, 6H), 1.32–1.18 (m, 4H), 0.97–0.92 (m, 3H), 0.95 (R^1: [CH(C**H**$_3$)$_2$], d, *J* = 7.1 Hz, 3H), 0.94 (R^2: [CH(C**H**$_3$)$_2$], *J* = 7.1 Hz, 3H), 0.84 (R^1: [CH(C**H**$_3$)$_2$], d, *J* = 7.0 Hz, 3H), 0.82 (R^2: [CH(C**H**$_3$)$_2$], d, *J* = 7.0 Hz, 3H). **^{13}C NMR** (126 MHz, $CDCl_3$ at 77.16 ppm): δ 162.8, 160.5, 156.9, 156.8, 156.8, 156.4, 156.4, 156.3, 71.4, 71.2, 61.3, 61.2, 61.2, 61.1, 61.1, 61.0, 57.7, 55.8, 42.8, 41.8, 41.8, 41.8, 38.8, 38.7, 37.9, 36.9, 31.4, 31.3, 29.8, 26.7, 26.6, 25.9, 25.8, 21.5, 21.5, 21.3, 20.3, 20.1, 20.0, 19.4, 15.4. **HRMS (ES+)**: 315.2068 (*m/z* calc'd for $C_{17}H_{28}N_2O_2$ $[M+Na]^+$:315.2048).

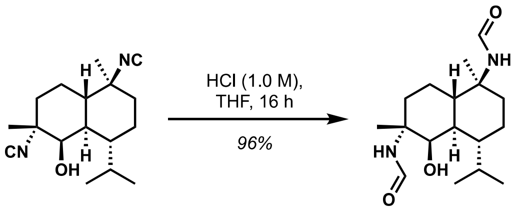

MED6-189 GB-209-3

Synthetic preparation of GB-209-3: To a 1-dram vial containing MED6-189 (4.8 mg, 17 µmol) and a magnetic stir bar was added THF (tetrahydrofuran, 1.7 mL) and hydrochloric acid (0.87 mL, 1.0 M, 870 µmol). The mixture was stirred at room temperature for 16 h. The volatiles were

removed under reduced pressure in a rotary evaporator then under high vacuum to afford bis-formamide GB-209-3 as a colorless oil that solidified upon standing (5.2 mg, 96% yield).

Note: GB-209-3 was observed as four different amide rotameric species (estimated ratio = 54:35:7:4) under ambient conditions, complicating NMR spectroscopic analysis:

^{1}H NMR (500 MHz, D_3COD, residual HD_2COD at 3.31 ppm): δ 8.29–7.87 (2H, m), 4.54–3.70 (1H, m), 2.13–1.94 (2H, m), 1.85–1.76 (1H, m), 1.76–1.66 (1H, m), 1.65–1.46 (6H, m), 1.41 (3H, s), 1.39–1.32 (1H, m), 1.32–1.25 (2H, m), 1.25–1.11 (2H, m), 0.97–0.89 (3H, m), 0.79–0.70 (3H, m). **^{13}C NMR** (126 MHz, D_3COD at 49.00 ppm): 166.4, 163.7, 163.6, 163.6, 68.2, 68.1, 59.3, 59.1, 58.4, 58.4, 58.3, 58.2, 43.0, 43.0, 42.9, 41.7, 41.6, 40.9, 39.6, 39.4, 38.5, 38.4, 32.3, 32.1, 28.8, 26.4, 26.4, 26.3, 24.3, 24.3, 24.2, 22.2, 22.1, 22.0, 22.0, 21.9, 21.6, 20.8, 20.8, 20.4, 19.2, 19.1, 18.2, 18.1, 18.1, 15.5. **HRMS (ES+)**: 333.2143 (*m/z* calc'd for $C_{17}H_{30}N_2O_3$ $[M+Na]^+$: 333.2154).

Time course stability of MED6-189 to conditions of varying pH:

Isonitriles are known to be somewhat reactive functional groups. While previous studies demonstrated that a closely related bis(isonitrile) compound had reasonable liver microsomal stability,[3] we also wanted to account for the potential stomach-acid-catalyzed hydrolysis of the isonitriles in the course of in vivo experiments. We developed a mass spectrometric assay to understand the acid stability of the isonitriles with respect to hydrolysis to the corresponding formamides. For this assay, we generated authentic samples of the hydrolysis products GB-209-2 (faster reacting isonitrile converted to mono-formamide) and GB-209-3 (both isonitriles hydrolyzed to form bis-formamide). Each of these compounds were tested against Plasmodium falciparum strain 3D7 (triplicate) and found to have very little antiplasmodial activity (IC_{50} of 3.8 and 3.9 µM, respectively). These data, coupled with the reasonable stability of MED6-189 toward acid hydrolysis down to pH 4, strongly supports that a significant quantity of MED6-189 survives passage through the gut, and it is the parent compound that is responsible for the antiplasmodial activity.

Pure samples of MED6-189, GB-209-2, and GB-209-3 were calibrated using a Micromass® Quattro Premier XE mass spectrometer with triple quadrupole (ES+) ionization parameters described in the table below. MED6-189 was calibrated using both a parent mass transition (275 – 221 Da) and a fragment mass transition (248 – 221 Da) due to its low parent ion count. With these parameters, calibration curves were generated by analyzing pure samples of each compound at concentrations (nm) shown in the Calibration curves below in acetonitrile:water = 20:80.

a.

Compound	M/Z Transition ($g*mol^{-1}$)	Dwell (s)	Cone Voltage (V)	Collision Energy (eV)	Delay (s)	t_R (min)
MED6-189	275 – 221	0.100	30.0	10.0	0.010	2.15
MED6-189	248 – 221	0.100	30.0	10.0	0.020	2.15
GB-209-2	293 – 203	0.100	20.0	10.0	0.010	1.70
GB-209-3	311 – 203	0.100	20.0	10.0	0.010	1.25

b.

Time (min)	Flow Rate ($mL*min^{-1}$)	%A	%B	Curve
Initial	0.3	90	10	
2.00	0.3	10	90	6
3.00	0.3	90	10	11
4.00	0.3	90	10	11

Supporting Table 1. a. Ionization (ES+) and ***b.*** inlet parameters for quantification of MED6-189 and formamides GB-209-2, and GB-209-3. Column Type: ACQUITY CSHTM Fluoro-Phenyl 1.7μm. Solvents A = formic acid (0.1% aq.) and B = acetonitrile.

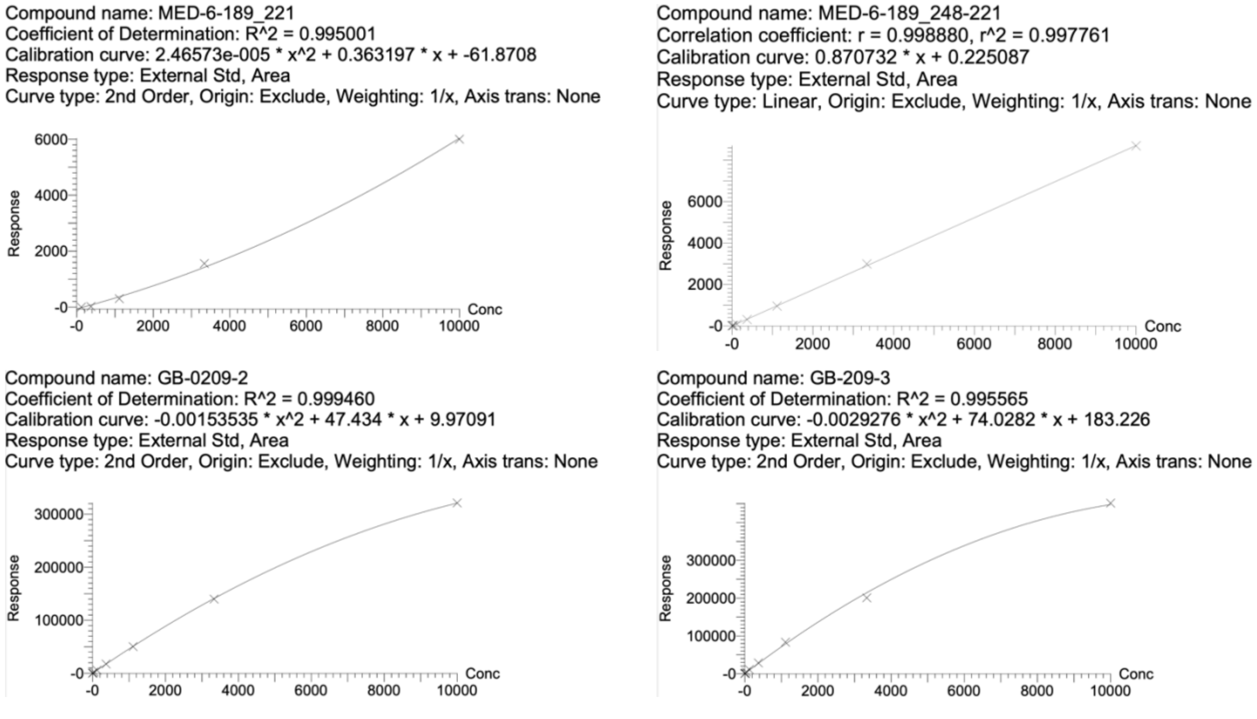

Calibration curves for MED6-189, GB-209-2, and GB-209-3.

A stock solution of MED6-189 (5.0 mM, 1.5 mg in 1.1 mL acetonitrile) was prepared. Aqueous buffer solutions of varying pH (400 μL, 0.1 M, KCl/HCl [pH = 2], sodium citrate/citric acid [pH = 3, 4, 5, 6], ammonium bicarbonate [pH = 7]) were charged with MED6-189 solution (100 μL)

at room temperature so that the substrate concentration was 1.0 mM. At various time points (10 min, 30 min, 1 h, 2 h, 4 h, 8 h, 16 h) aliquots (10.0 μL) from each reaction mixture were diluted 300–fold using two serial 17.3-fold dilutions in neutral buffer solution (acetonitrile:ammonium bicarbonate solution [0.1 M, aqueous] = 1:4) so that the total concentration of substrate and products was approximately 3.3 μM. Diluted aliquots were immediately frozen at –20 ºC until analysis the following day. Using the calibration curves above, the concentrations of MED6-189, GB-209-2 and GB-209-3 were determined for each aliquot and plotted over time for respective pH values in the figure below describing the time course stability of MED6-189 to conditions of varying pH over time via quantitative analysis of formamide products GB-209-2 and GB-209-3.

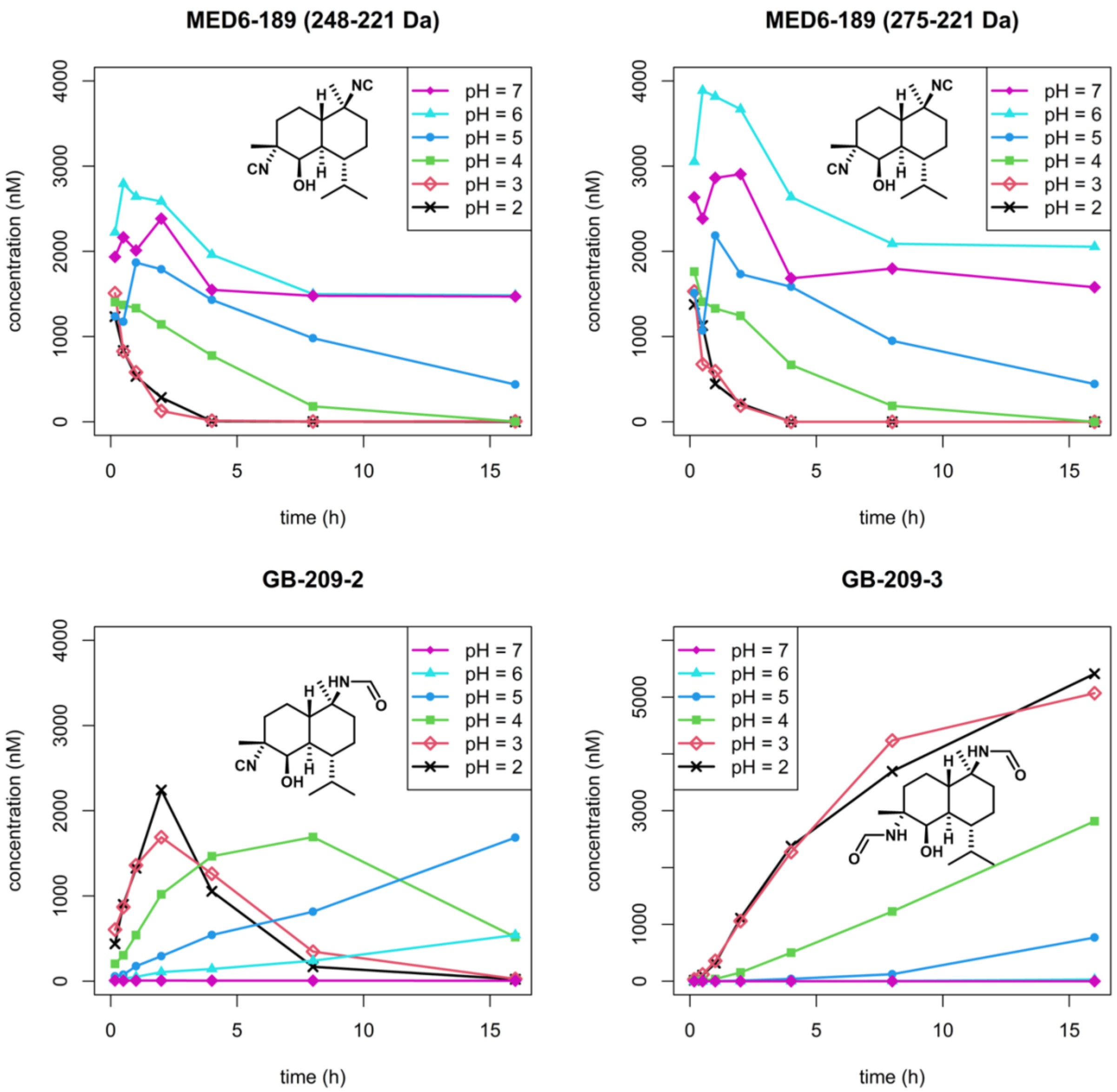

Time course stability of MED6-189 to conditions of varying pH over time via quantitative analysis of formamide products GB-209-2 and GB-209-3.

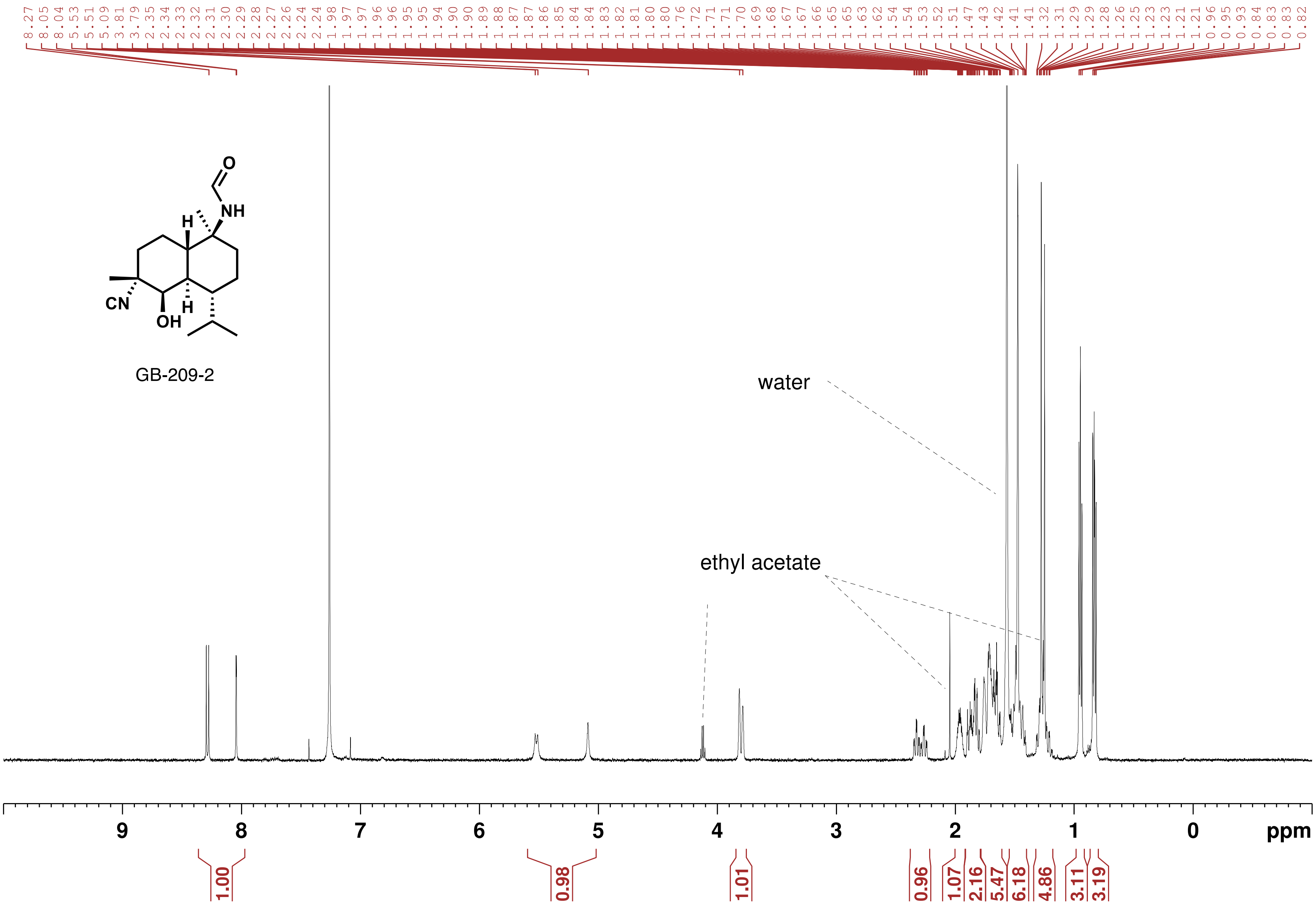
8.27 8.05 8.04 5.53 5.51 5.09 3.81 3.79 2.35 2.34 2.33 2.32 2.31 2.30 2.29 2.28 2.27 2.26 2.24 2.24 1.98 1.97 1.97 1.96 1.96 1.95 1.95 1.94 1.90 1.90 1.89 1.88 1.87 1.87 1.86 1.85 1.84 1.84 1.83 1.82 1.81 1.80 1.80 1.76 1.72 1.71 1.71 1.70 1.69 1.68 1.67 1.67 1.66 1.65 1.65 1.63 1.62 1.54 1.54 1.53 1.52 1.51 1.47 1.43 1.42 1.41 1.41 1.32 1.31 1.29 1.29 1.28 1.26 1.25 1.23 1.23 1.21 1.21 0.96 0.95 0.93 0.84 0.83 0.83 0.82
O
NH
H
H
CN
OH
GB-209-2
water
ethyl acetate
9
8
7
6
5
4
3
2
1
0
ppm
1.00
0.98
1.01
0.96
1.07
2.16
5.47
6.18
4.86
3.11
3.19

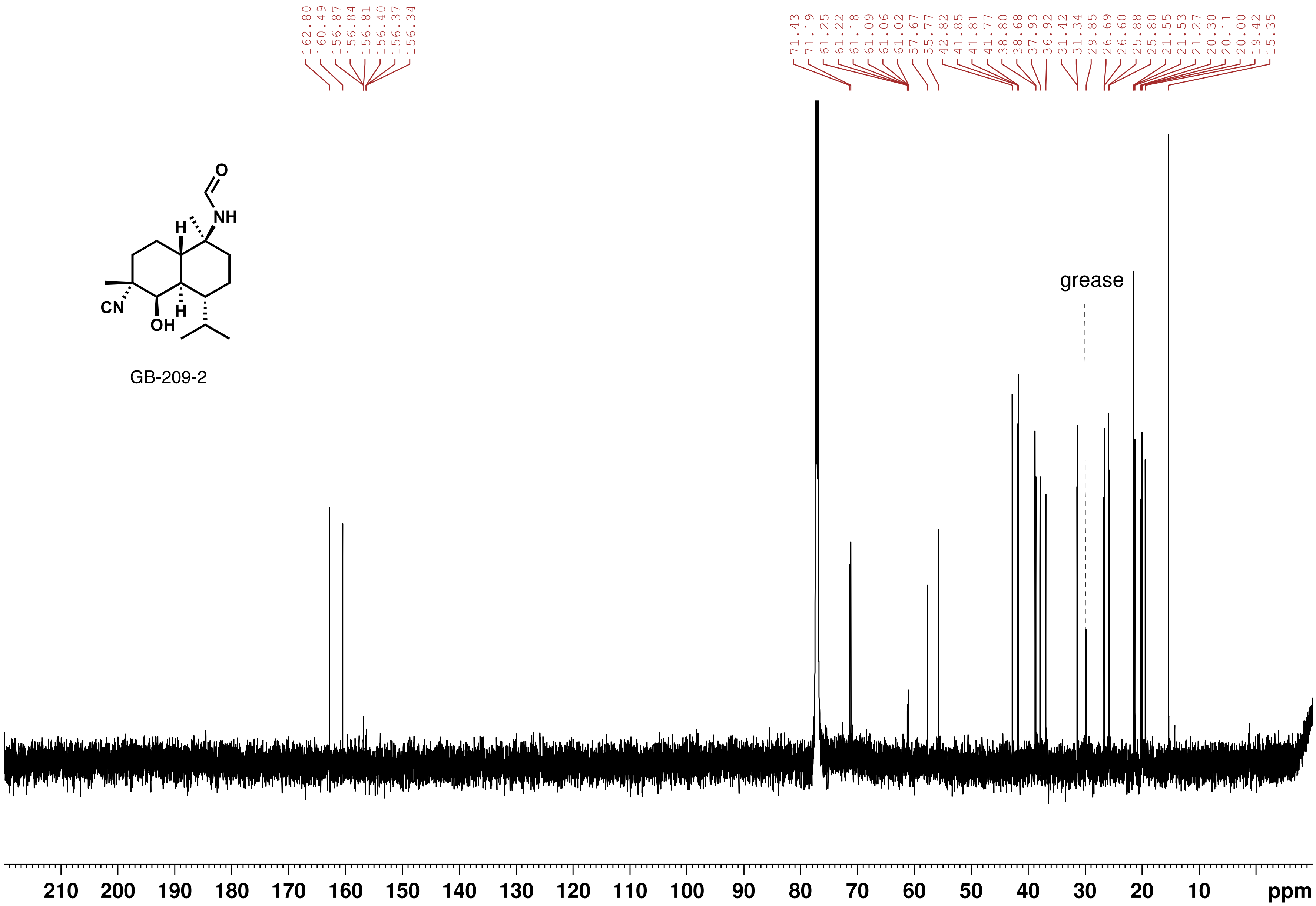
CN
OH
H
H
NH
O
162.80
160.49
156.87
156.84
156.81
156.40
156.37
156.34
71.43
71.19
61.25
61.22
61.18
61.09
61.06
61.02
57.67
55.77
42.82
41.85
41.81
41.77
38.80
38.68
37.93
36.92
31.42
31.34
29.85
26.69
26.60
25.88
25.80
21.55
21.53
21.27
20.30
20.11
20.00
19.42
15.35
grease
210
200
190
180
170
160
150
140
130
120
110
100
90
80
70
60
50
40
30
20
10
ppm

GB-209-2

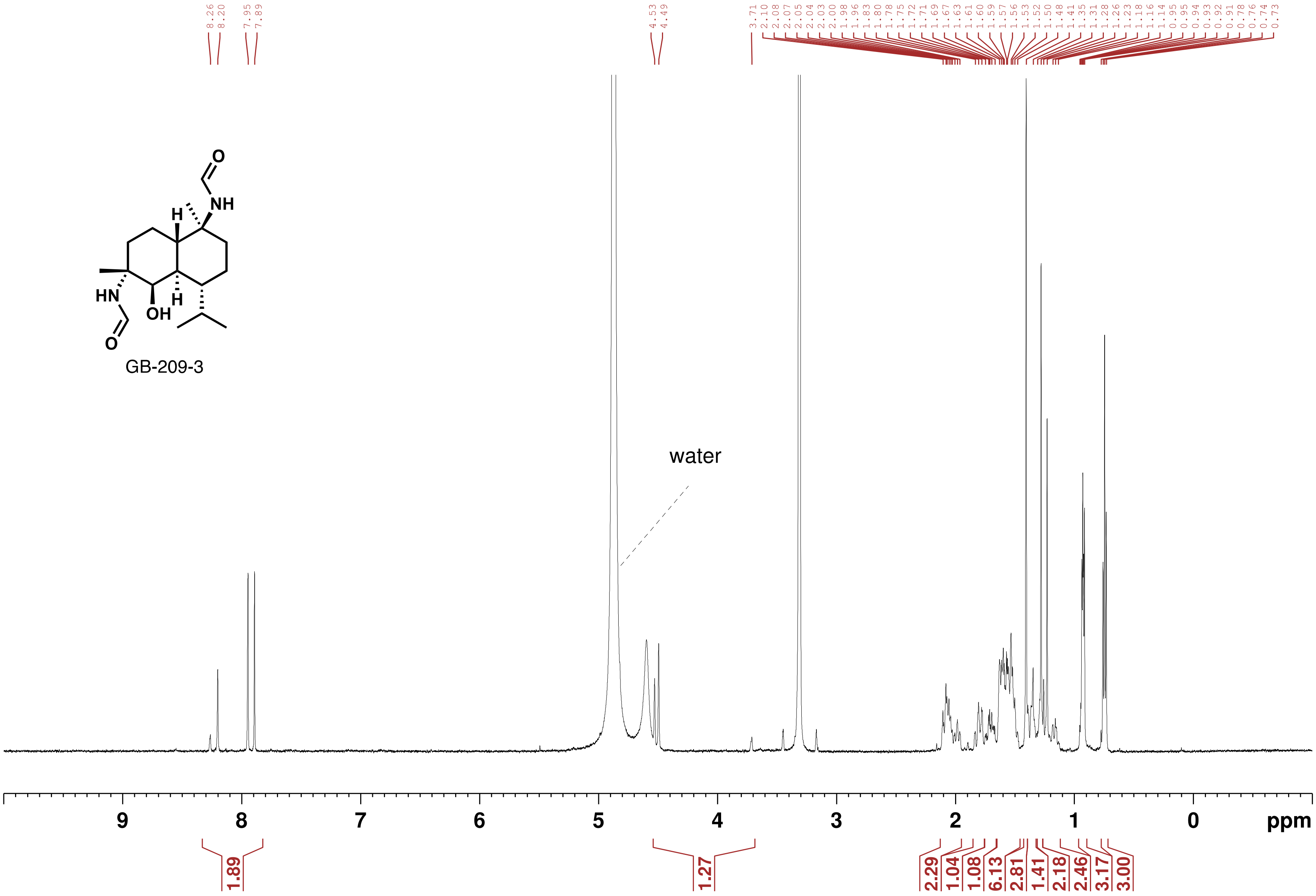
8.26
8.20
7.95
7.89
4.53
4.49
3.71
2.10
2.08
2.07
2.05
2.04
2.03
2.00
1.98
1.96
1.83
1.80
1.78
1.75
1.72
1.71
1.69
1.67
1.63
1.61
1.60
1.59
1.57
1.56
1.53
1.52
1.50
1.48
1.41
1.35
1.31
1.28
1.26
1.23
1.18
1.16
1.14
0.95
0.95
0.94
0.93
0.92
0.91
0.78
0.76
0.74
0.73
water
9
8
7
6
5
4
3
2
1
0
ppm
1.89
1.27
2.29
1.04
1.08
6.13
2.81
1.41
2.18
2.46
3.17
3.00

GB-209-3

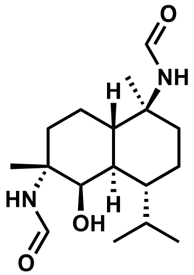

GB-209-3

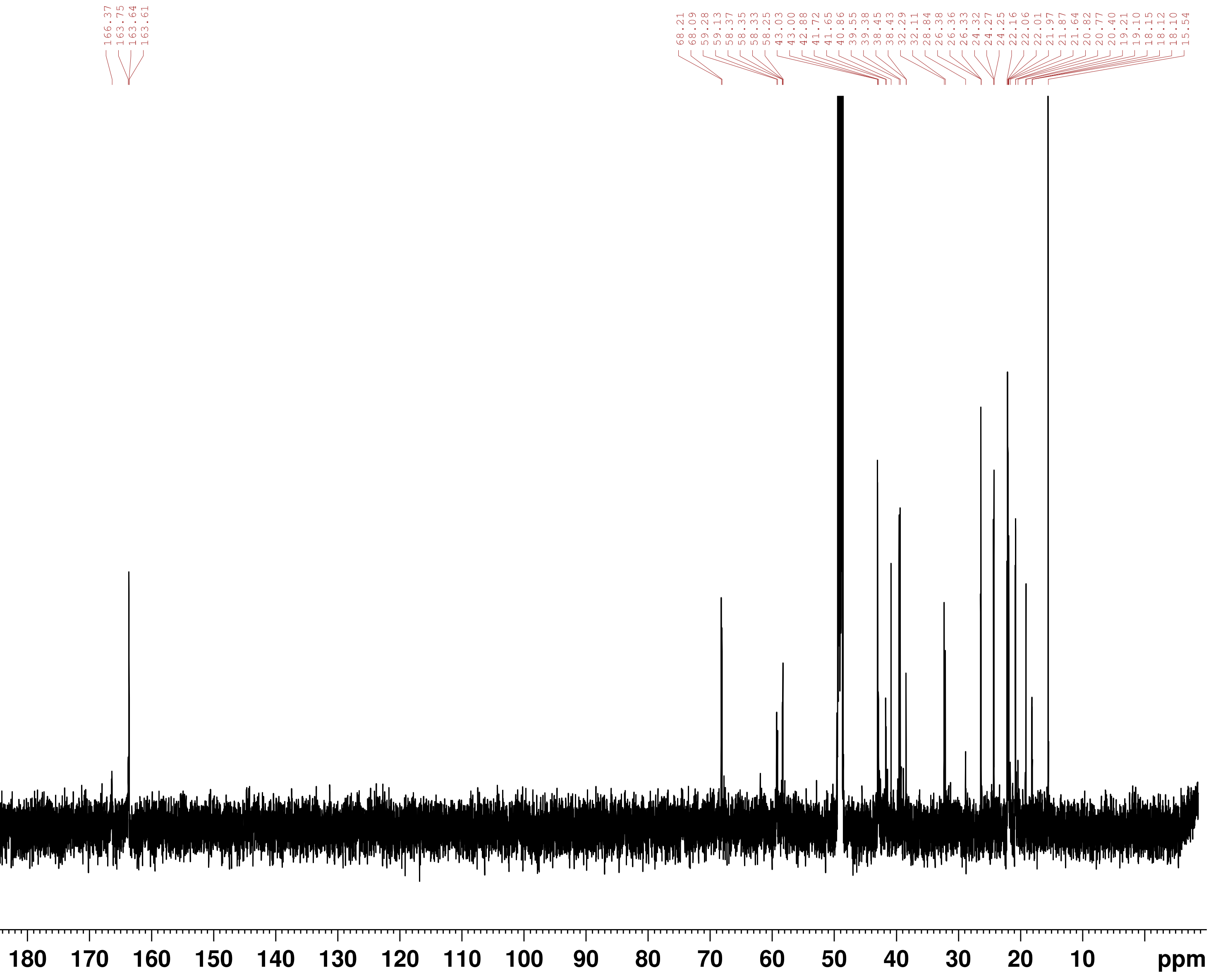

166.37
163.75
163.64
163.61
68.21
68.09
59.28
59.13
58.37
58.35
58.33
58.25
43.03
43.00
42.88
41.72
41.65
40.86
39.55
39.38
38.45
38.43
32.29
32.11
28.84
26.38
26.36
26.33
24.32
24.27
24.25
22.16
22.06
22.01
21.97
21.87
21.64
20.82
20.77
20.40
19.21
19.10
18.15
18.12
18.10
15.54
210 200 190 180 170 160 150 140 130 120 110 100 90 80 70 60 50 40 30 20 10 ppm

Supporting Information S2

TPP curves plotted for heat challenged samples treated with DMSO (Controls) or MED6-189 (MED6)

TPP curves are presented in order with decreasing euclidian distance (ED) scores for each condition:

MED6-189 (red) vs. DMSO (blue)

Med6 TPP data plotting_curve fitting

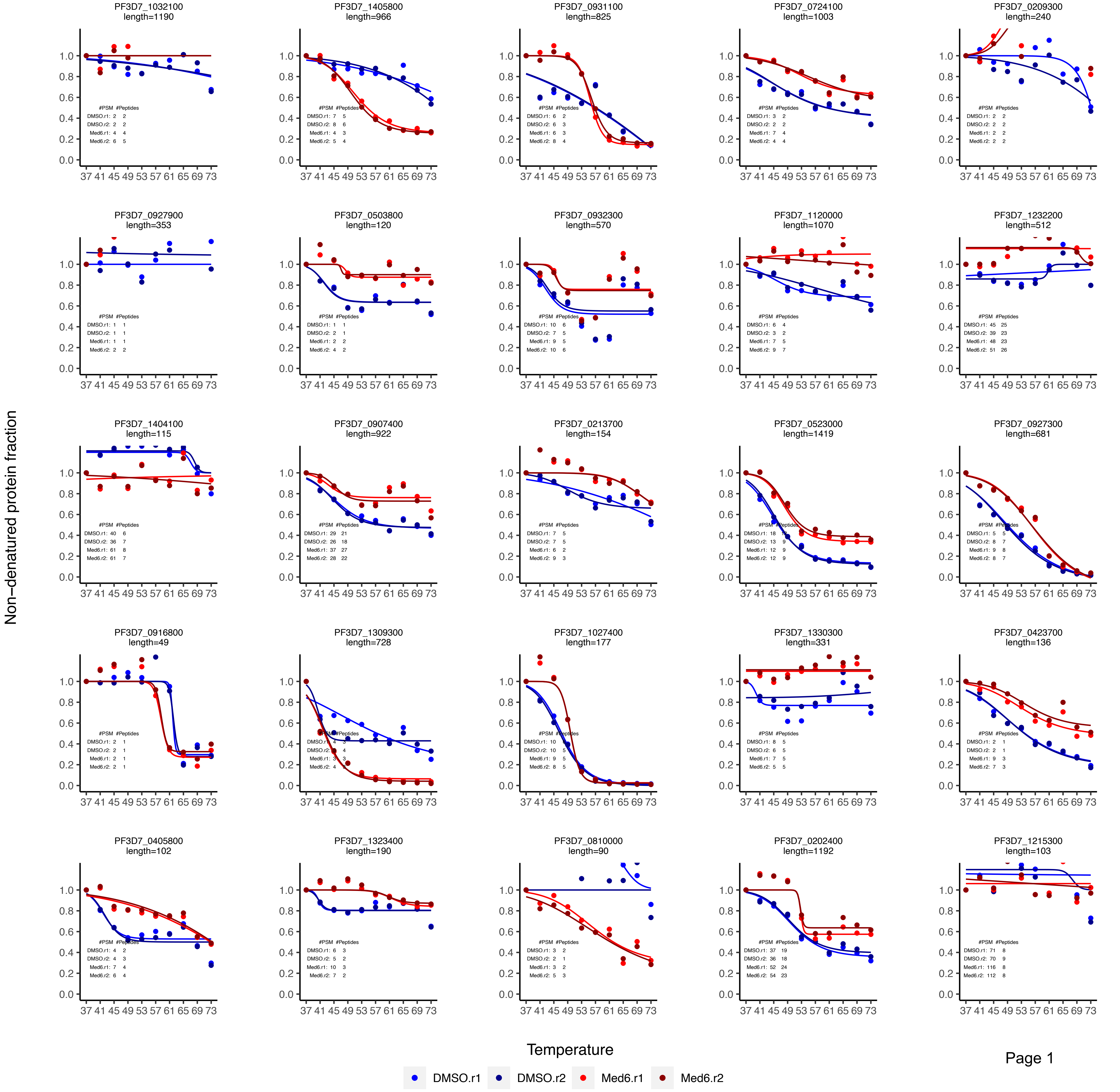

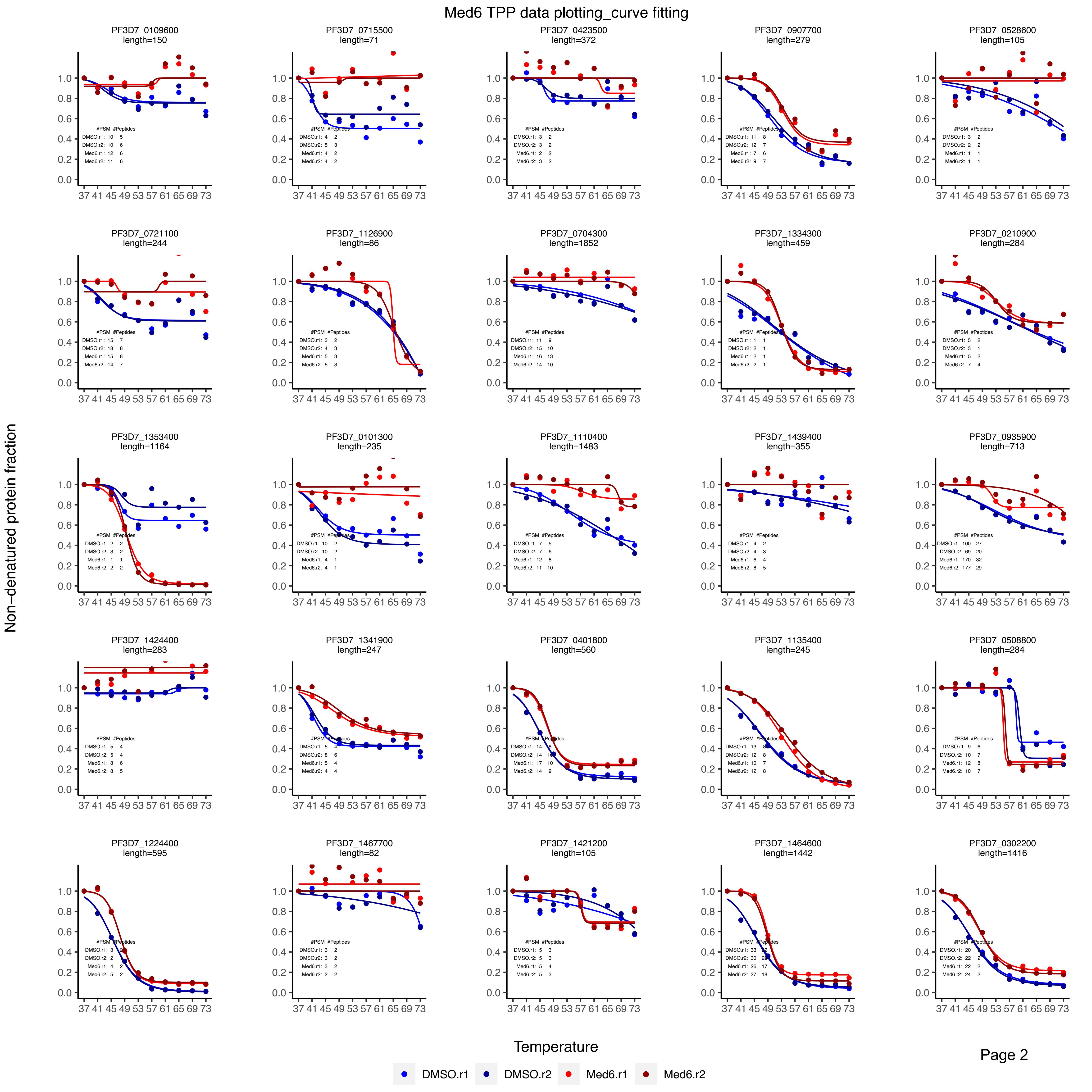
Med6 TPP data plotting_curve fitting
PF3D7_0109600 length=150
PF3D7_0715500 length=71
PF3D7_0423500 length=372
PF3D7_0907700 length=279
PF3D7_0528600 length=105
PF3D7_0721100 length=244
PF3D7_1126900 length=86
PF3D7_0704300 length=1852
PF3D7_1334300 length=459
PF3D7_0210900 length=284
PF3D7_1353400 length=1164
PF3D7_0101300 length=235
PF3D7_1110400 length=1483
PF3D7_1439400 length=355
PF3D7_0935900 length=713
PF3D7_1424400 length=283
PF3D7_1341900 length=247
PF3D7_0401800 length=560
PF3D7_1135400 length=245
PF3D7_0508800 length=284
PF3D7_1224400 length=595
PF3D7_1467700 length=82
PF3D7_1421200 length=105
PF3D7_1464600 length=1442
PF3D7_0302200 length=1416
Non−denatured protein fraction
Temperature
DMSO.r1
DMSO.r2
Med6.r1
Med6.r2
Page 2

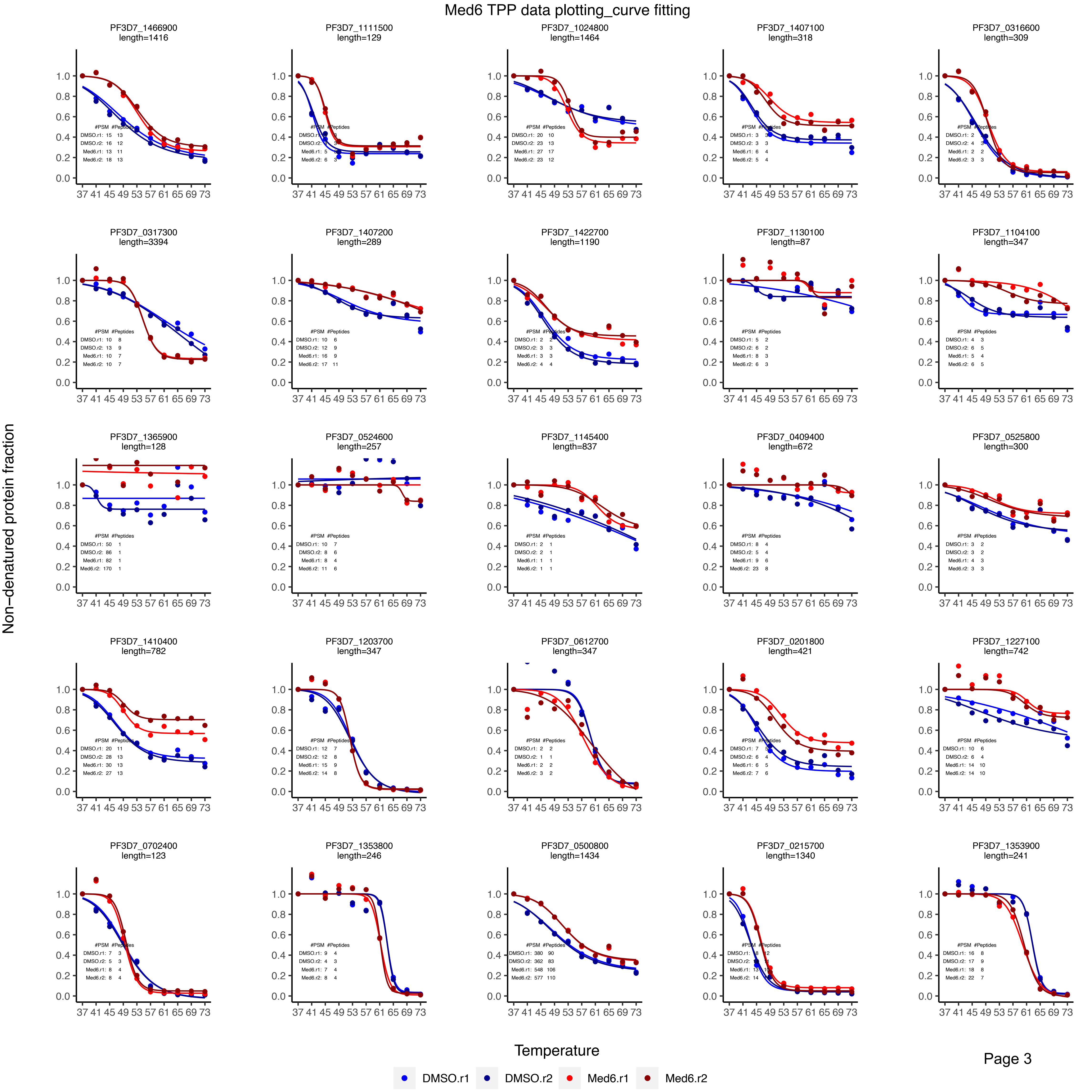

Med6 TPP data plotting_curve fitting
PF3D7_1466900 length=1416
PF3D7_1111500 length=129
PF3D7_1024800 length=1464
PF3D7_1407100 length=318
PF3D7_0316600 length=309
PF3D7_0317300 length=3394
PF3D7_1407200 length=289
PF3D7_1422700 length=1190
PF3D7_1130100 length=87
PF3D7_1104100 length=347
PF3D7_1365900 length=128
PF3D7_0524600 length=257
PF3D7_1145400 length=837
PF3D7_0409400 length=672
PF3D7_0525800 length=300
PF3D7_1410400 length=782
PF3D7_1203700 length=347
PF3D7_0612700 length=347
PF3D7_0201800 length=421
PF3D7_1227100 length=742
PF3D7_0702400 length=123
PF3D7_1353800 length=246
PF3D7_0500800 length=1434
PF3D7_0215700 length=1340
PF3D7_1353900 length=241
Non−denatured protein fraction
Temperature
DMSO.r1
DMSO.r2
Med6.r1
Med6.r2
Page 3

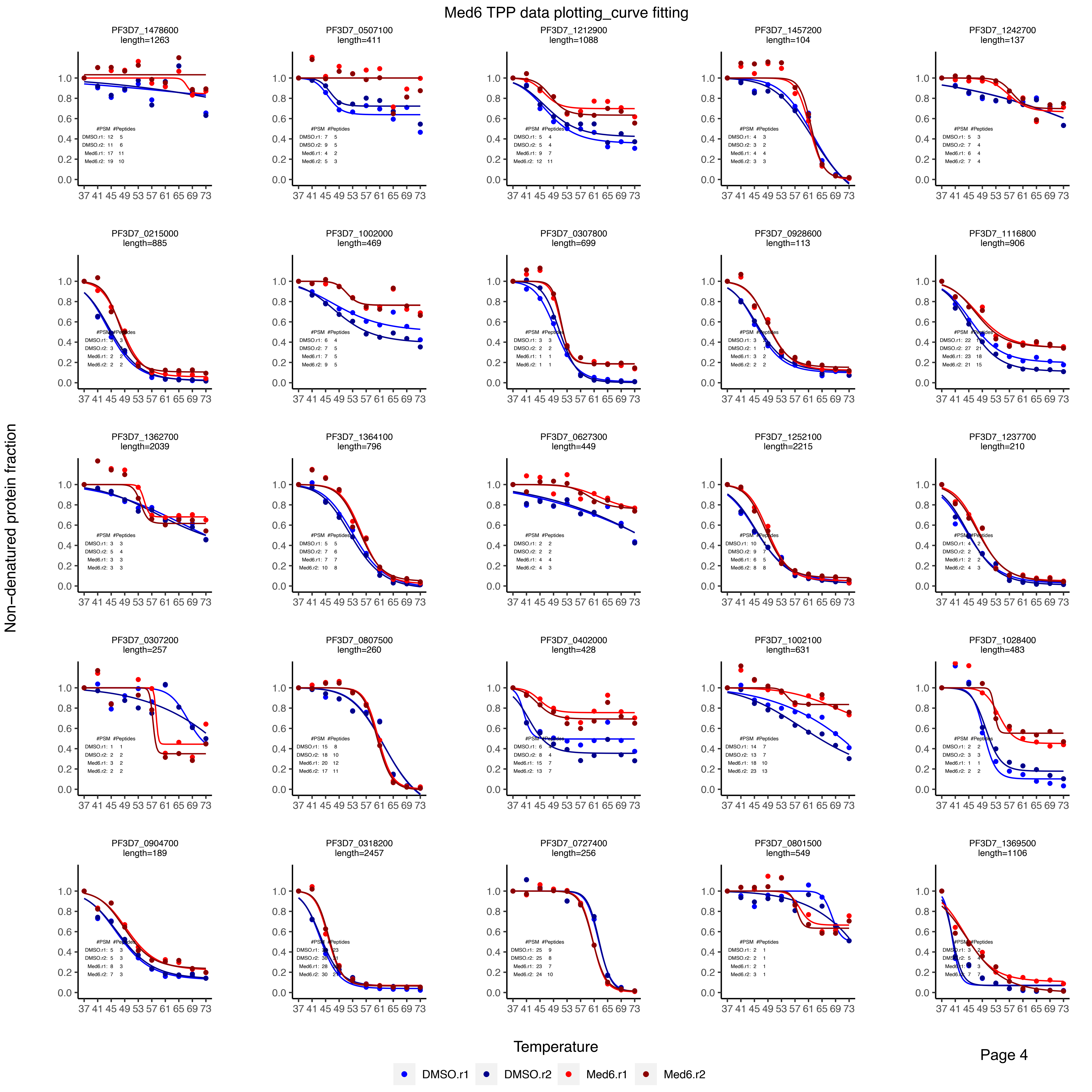
Med6 TPP data plotting_curve fitting
PF3D7_1478600 length=1263
PF3D7_0507100 length=411
PF3D7_1212900 length=1088
PF3D7_1457200 length=104
PF3D7_1242700 length=137
PF3D7_0215000 length=885
PF3D7_1002000 length=469
PF3D7_0307800 length=699
PF3D7_0928600 length=113
PF3D7_1116800 length=906
PF3D7_1362700 length=2039
PF3D7_1364100 length=796
PF3D7_0627300 length=449
PF3D7_1252100 length=2215
PF3D7_1237700 length=210
PF3D7_0307200 length=257
PF3D7_0807500 length=260
PF3D7_0402000 length=428
PF3D7_1002100 length=631
PF3D7_1028400 length=483
PF3D7_0904700 length=189
PF3D7_0318200 length=2457
PF3D7_0727400 length=256
PF3D7_0801500 length=549
PF3D7_1369500 length=1106
Non−denatured protein fraction
Temperature
DMSO.r1
DMSO.r2
Med6.r1
Med6.r2
Page 4

Med6 TPP data plotting_curve fitting

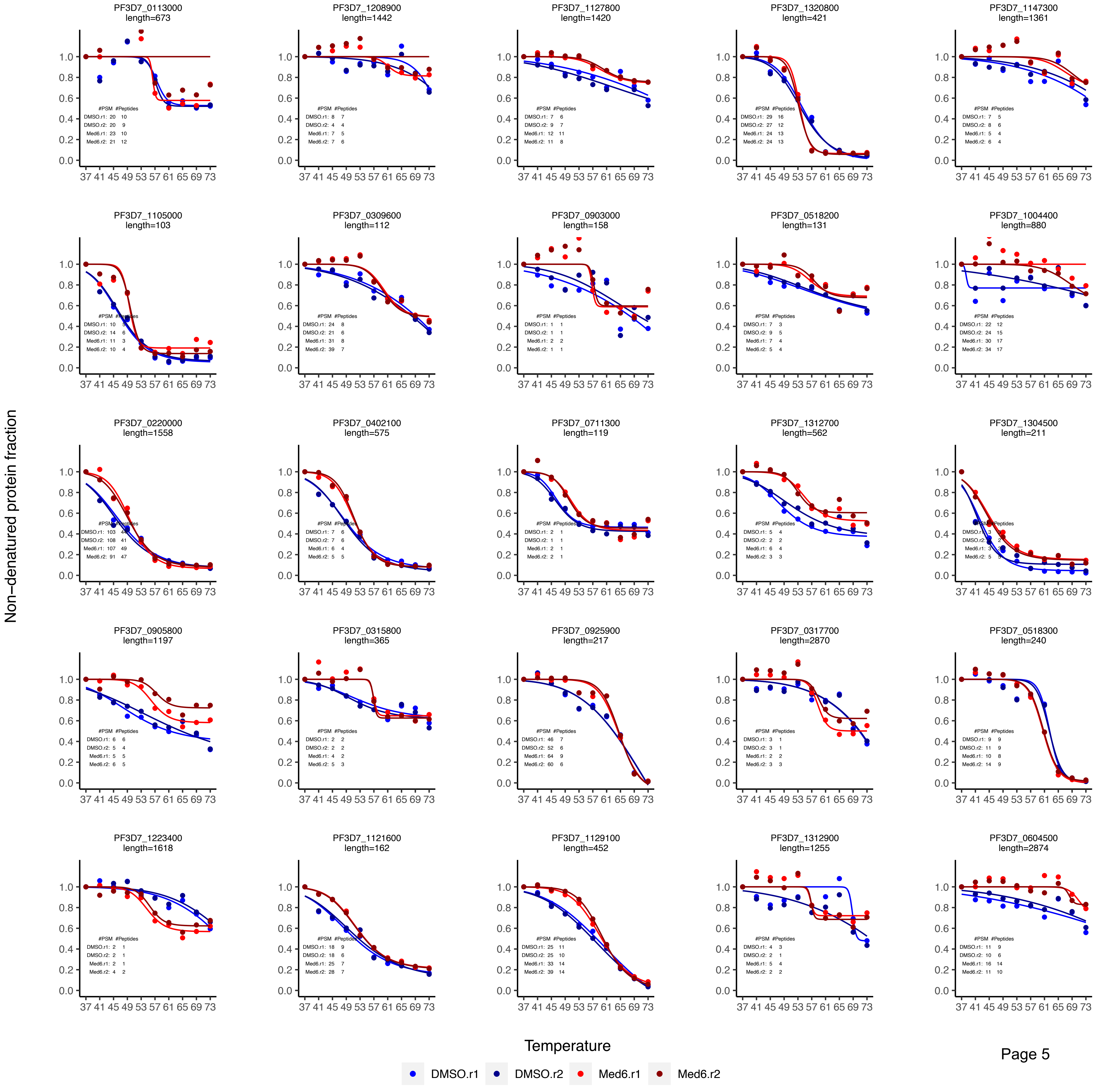

Med6 TPP data plotting_curve fitting

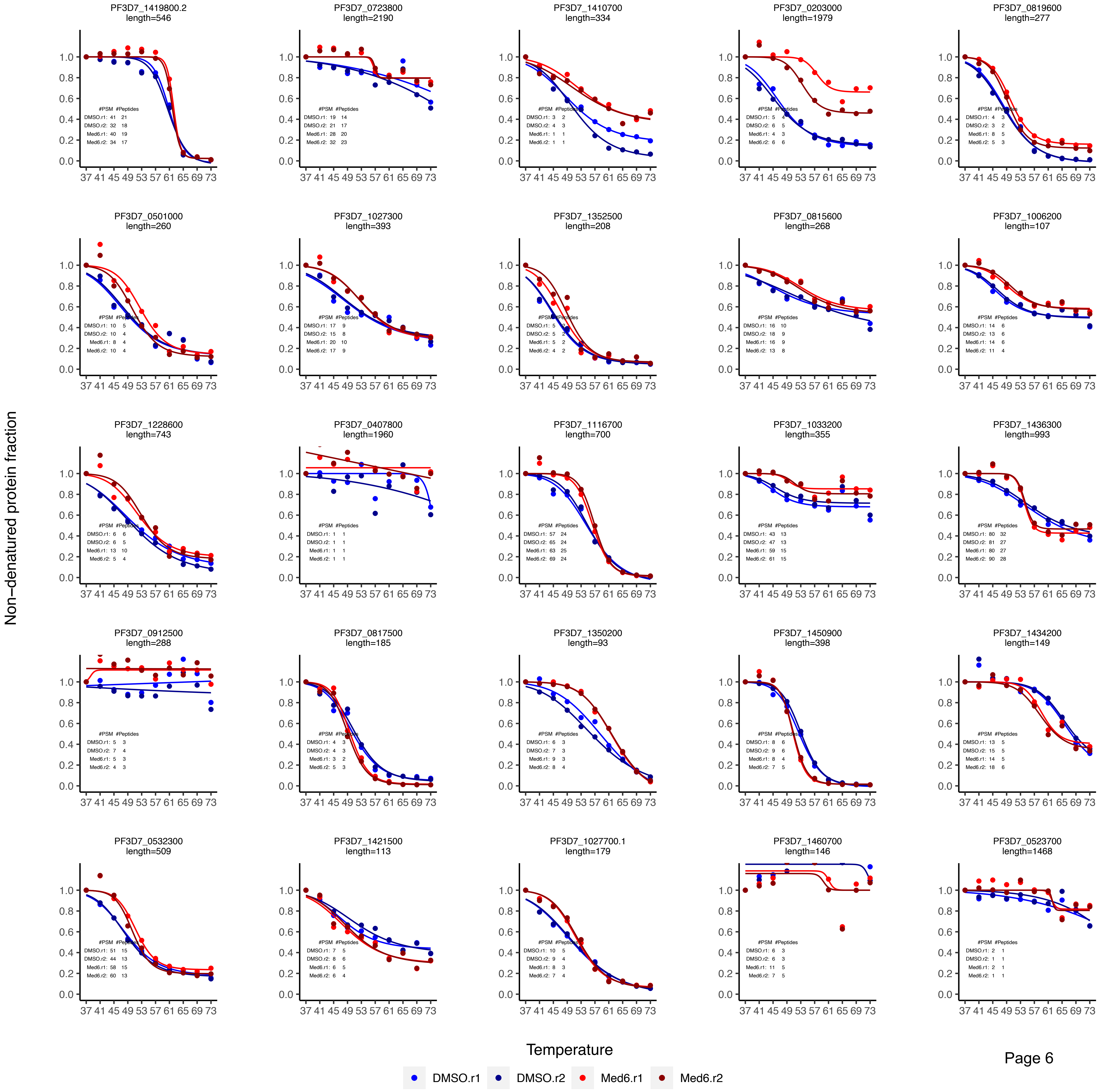

Med6 TPP data plotting_curve fitting

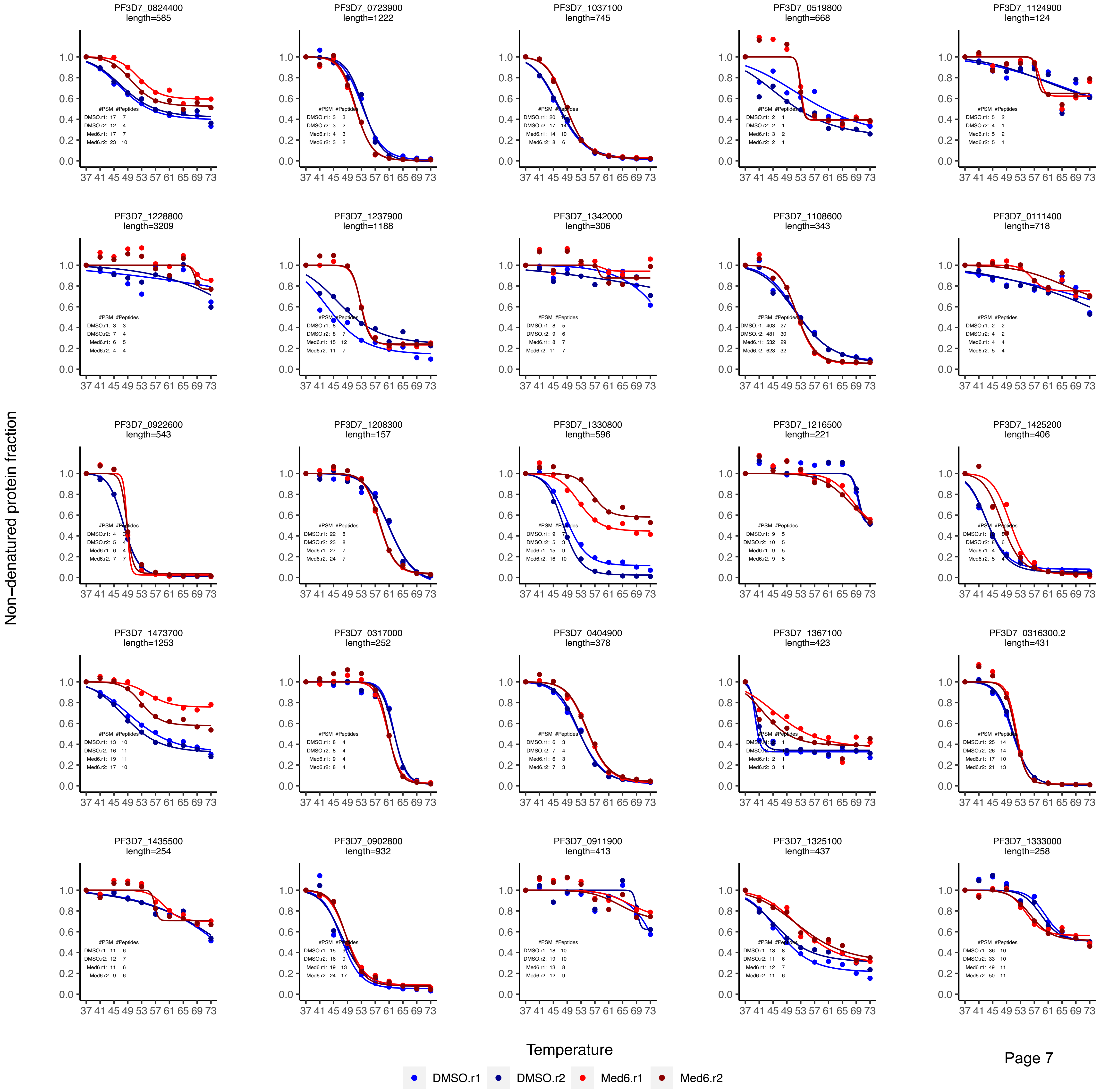

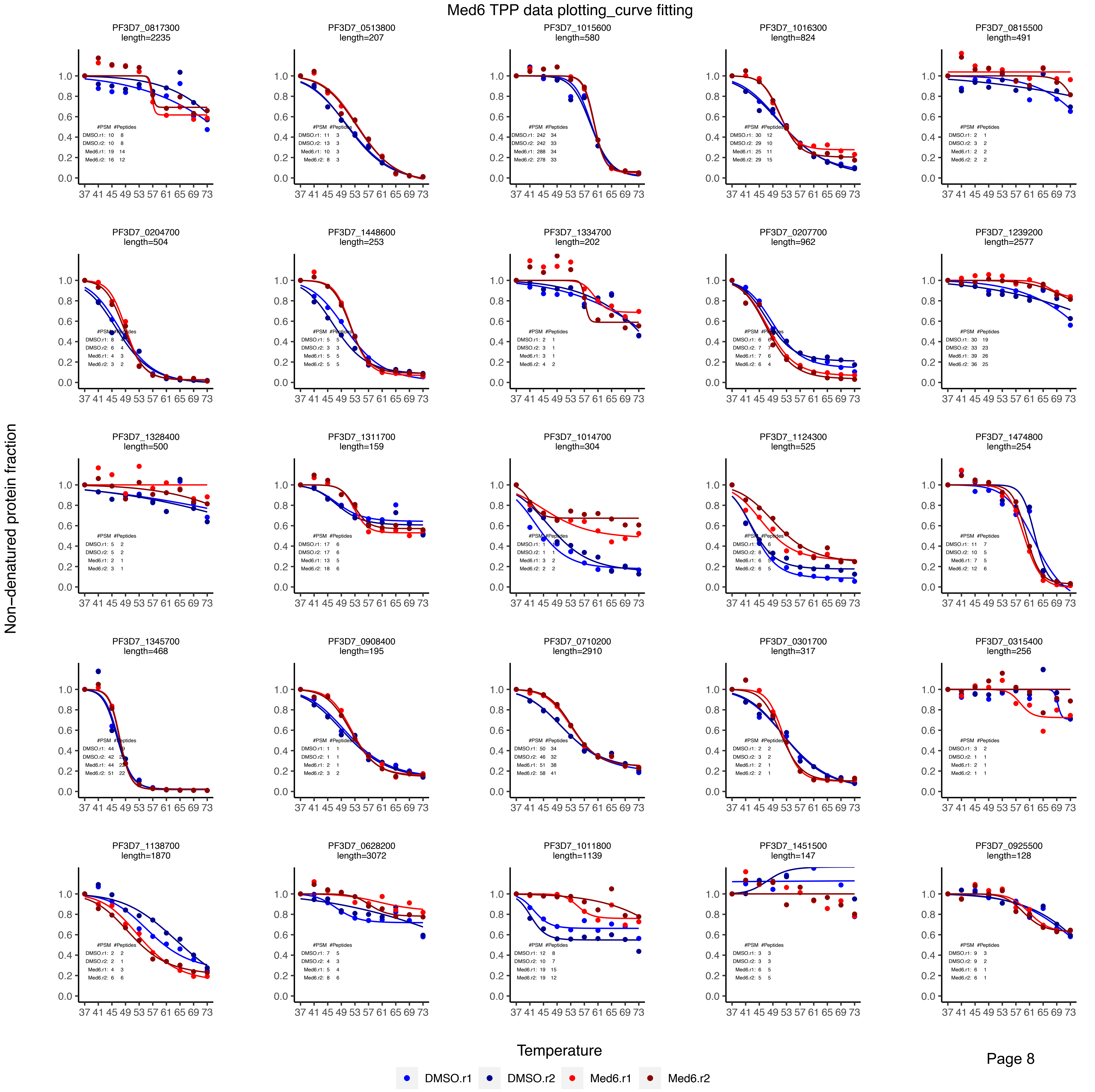

Med6 TPP data plotting_curve fitting
PF3D7_0817300 length=2235
PF3D7_0513800 length=207
PF3D7_1015600 length=580
PF3D7_1016300 length=824
PF3D7_0815500 length=491
PF3D7_0204700 length=504
PF3D7_1448600 length=253
PF3D7_1334700 length=202
PF3D7_0207700 length=962
PF3D7_1239200 length=2577
PF3D7_1328400 length=500
PF3D7_1311700 length=159
PF3D7_1014700 length=304
PF3D7_1124300 length=525
PF3D7_1474800 length=254
PF3D7_1345700 length=468
PF3D7_0908400 length=195
PF3D7_0710200 length=2910
PF3D7_0301700 length=317
PF3D7_0315400 length=256
PF3D7_1138700 length=1870
PF3D7_0628200 length=3072
PF3D7_1011800 length=1139
PF3D7_1451500 length=147
PF3D7_0925500 length=128
Non−denatured protein fraction
Temperature
DMSO.r1
DMSO.r2
Med6.r1
Med6.r2

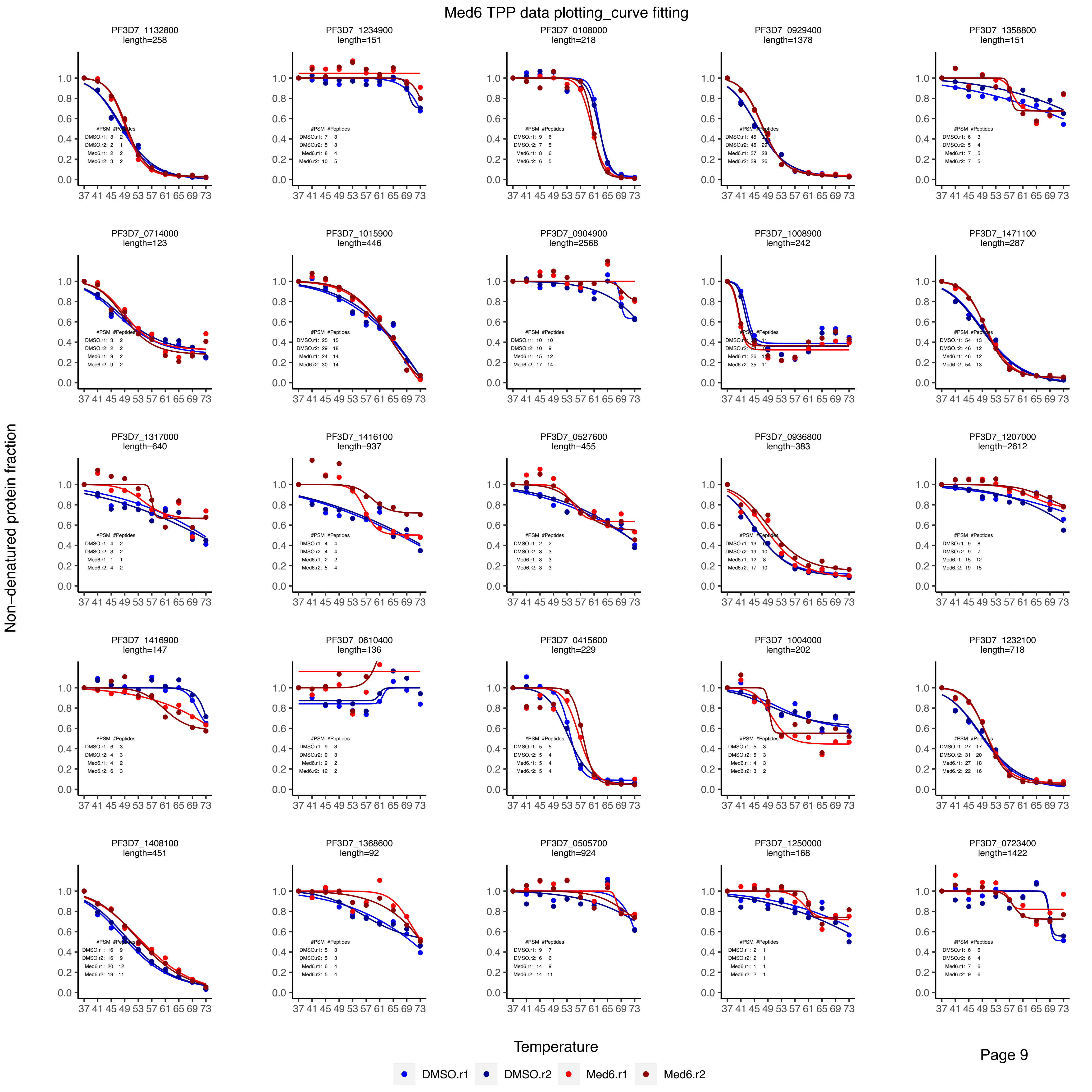

Med6 TPP data plotting_curve fitting
PF3D7_1132800 length=258
PF3D7_1234900 length=151
PF3D7_0108000 length=218
PF3D7_0929400 length=1378
PF3D7_1358800 length=151
PF3D7_0714000 length=123
PF3D7_1015900 length=446
PF3D7_0904900 length=2568
PF3D7_1008900 length=242
PF3D7_1471100 length=287
PF3D7_1317000 length=640
PF3D7_1416100 length=937
PF3D7_0527600 length=455
PF3D7_0936800 length=383
PF3D7_1207000 length=2612
PF3D7_1416900 length=147
PF3D7_0610400 length=136
PF3D7_0415600 length=229
PF3D7_1004000 length=202
PF3D7_1232100 length=718
PF3D7_1408100 length=451
PF3D7_1368600 length=92
PF3D7_0505700 length=924
PF3D7_1250000 length=168
PF3D7_0723400 length=1422
Non−denatured protein fraction
Temperature
DMSO.r1
DMSO.r2
Med6.r1
Med6.r2
Page 9

Med6 TPP data plotting_curve fitting

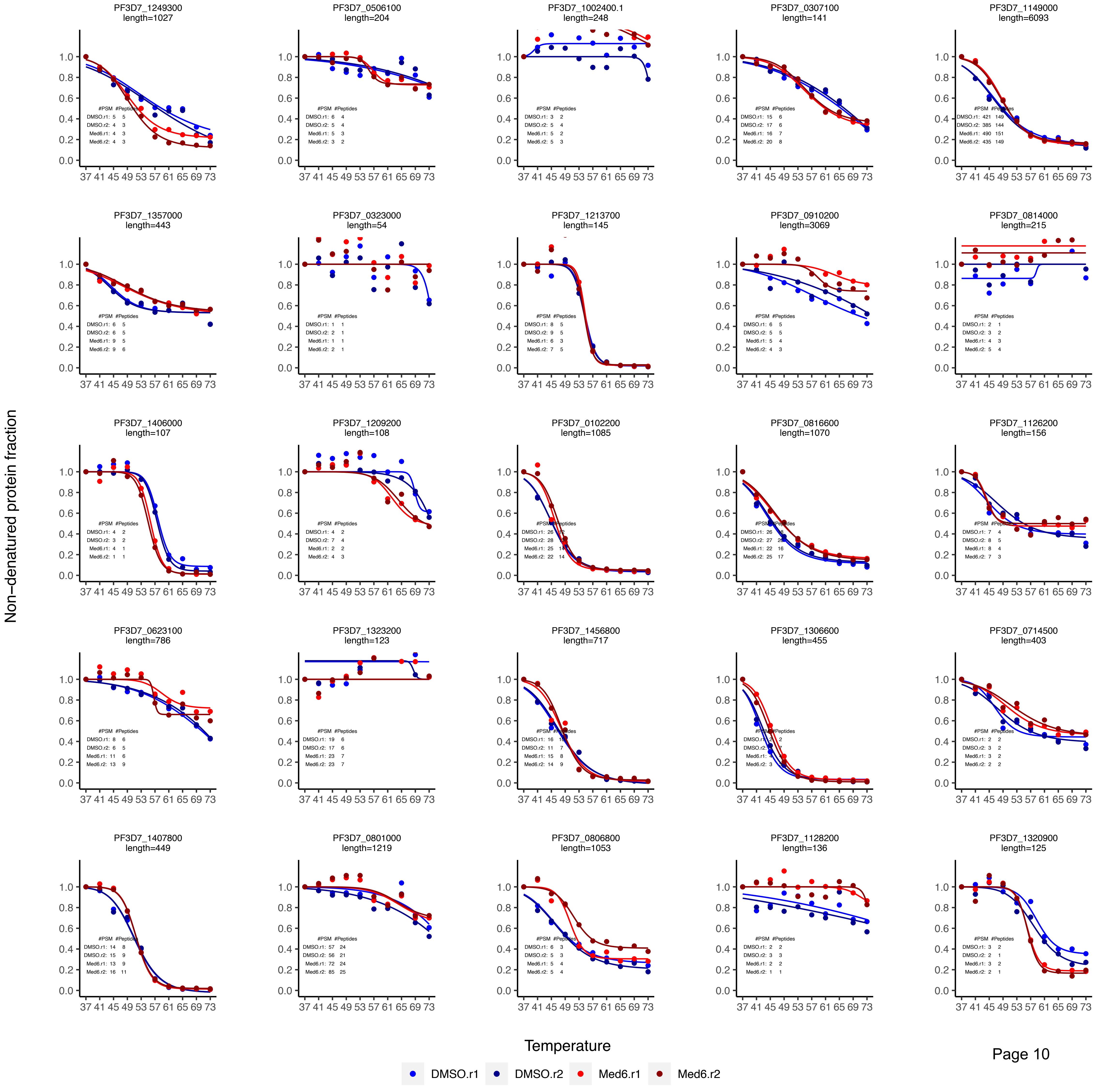

Med6 TPP data plotting_curve fitting

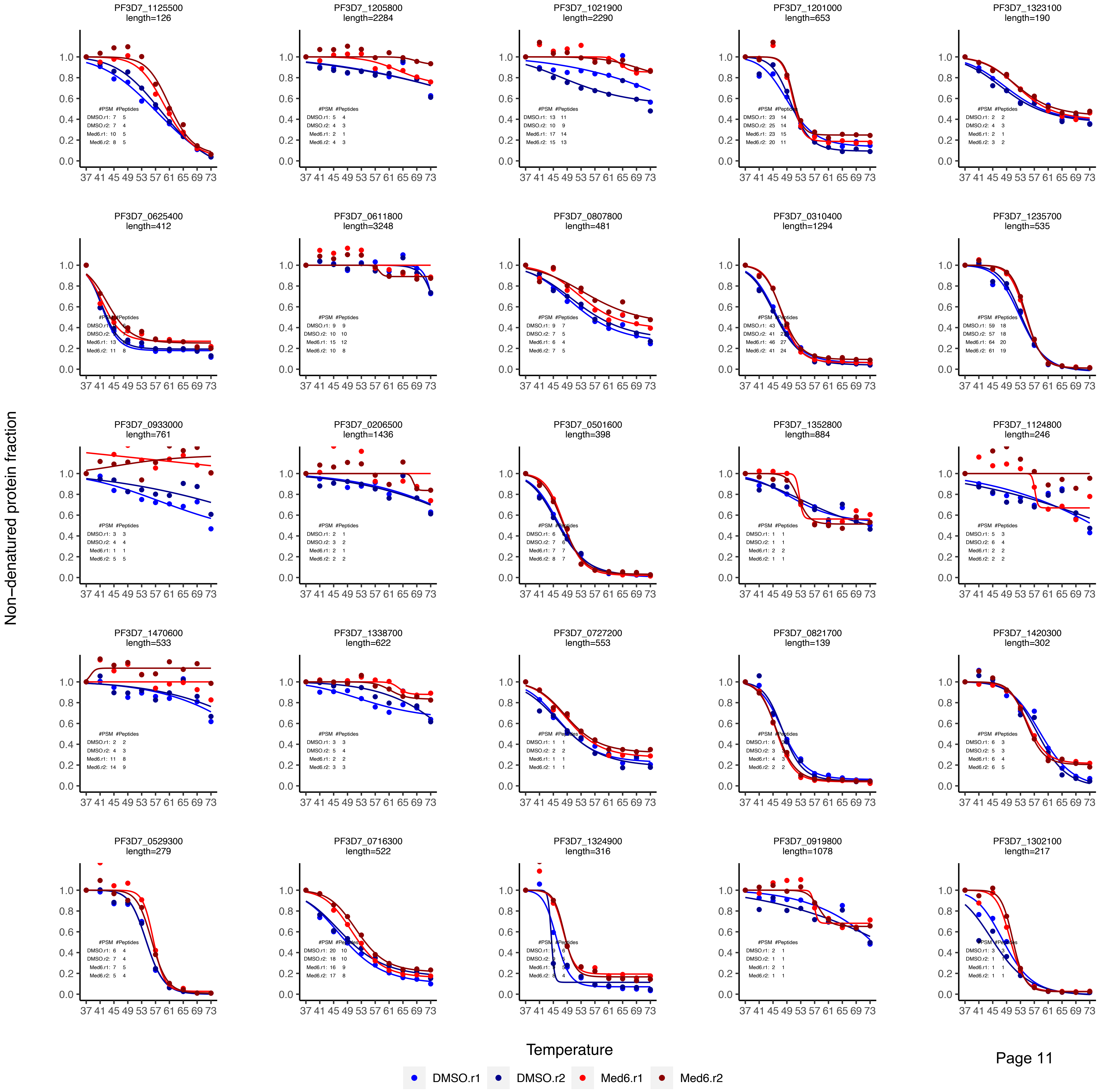

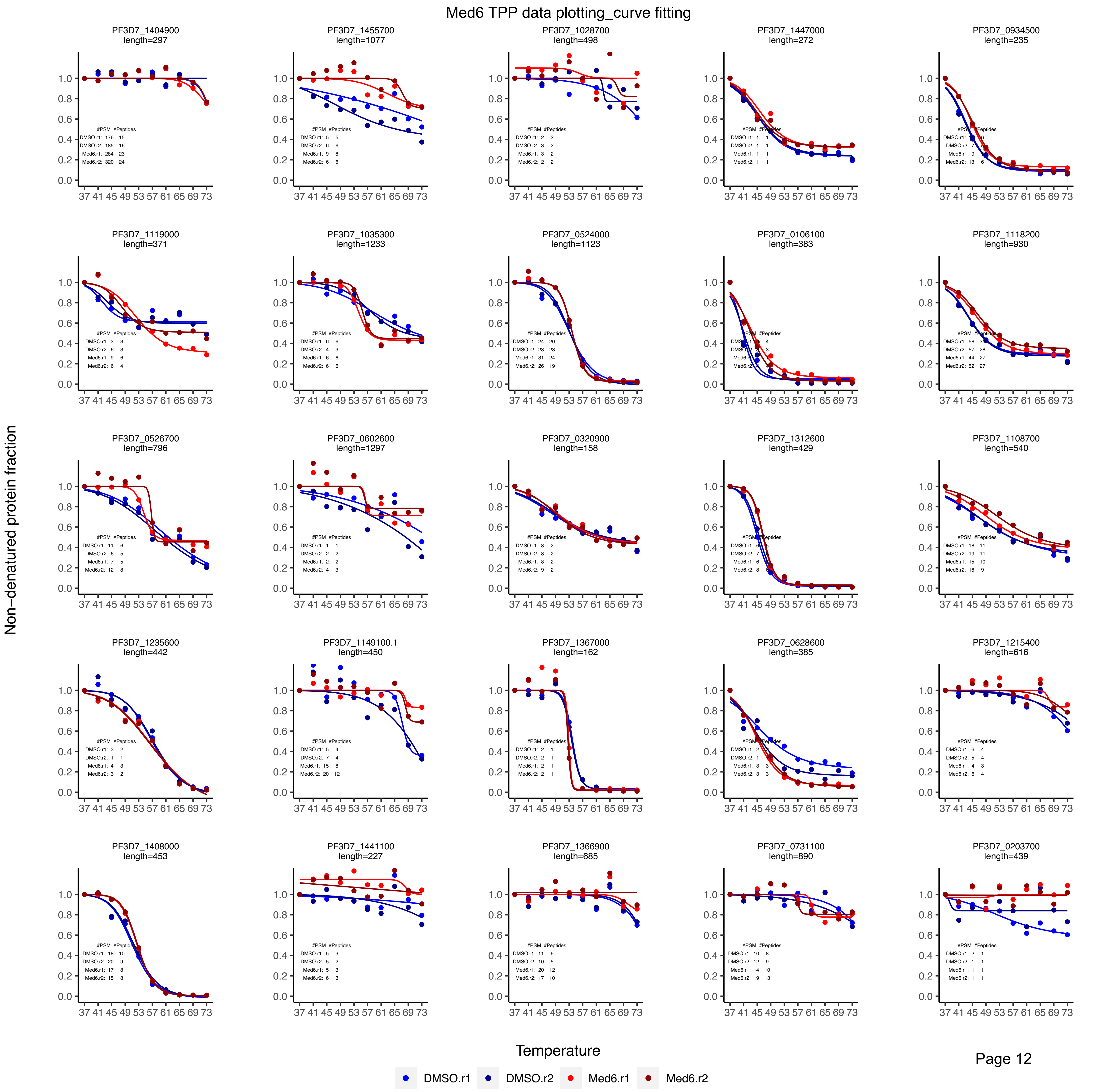
Med6 TPP data plotting_curve fitting
PF3D7_1404900 length=297
PF3D7_1455700 length=1077
PF3D7_1028700 length=498
PF3D7_1447000 length=272
PF3D7_0934500 length=235
PF3D7_1119000 length=371
PF3D7_1035300 length=1233
PF3D7_0524000 length=1123
PF3D7_0106100 length=383
PF3D7_1118200 length=930
PF3D7_0526700 length=796
PF3D7_0602600 length=1297
PF3D7_0320900 length=158
PF3D7_1312600 length=429
PF3D7_1108700 length=540
PF3D7_1235600 length=442
PF3D7_1149100.1 length=450
PF3D7_1367000 length=162
PF3D7_0628600 length=385
PF3D7_1215400 length=616
PF3D7_1408000 length=453
PF3D7_1441100 length=227
PF3D7_1366900 length=685
PF3D7_0731100 length=890
PF3D7_0203700 length=439
Non−denatured protein fraction
Temperature
DMSO.r1
DMSO.r2
Med6.r1
Med6.r2

Med6 TPP data plotting_curve fitting

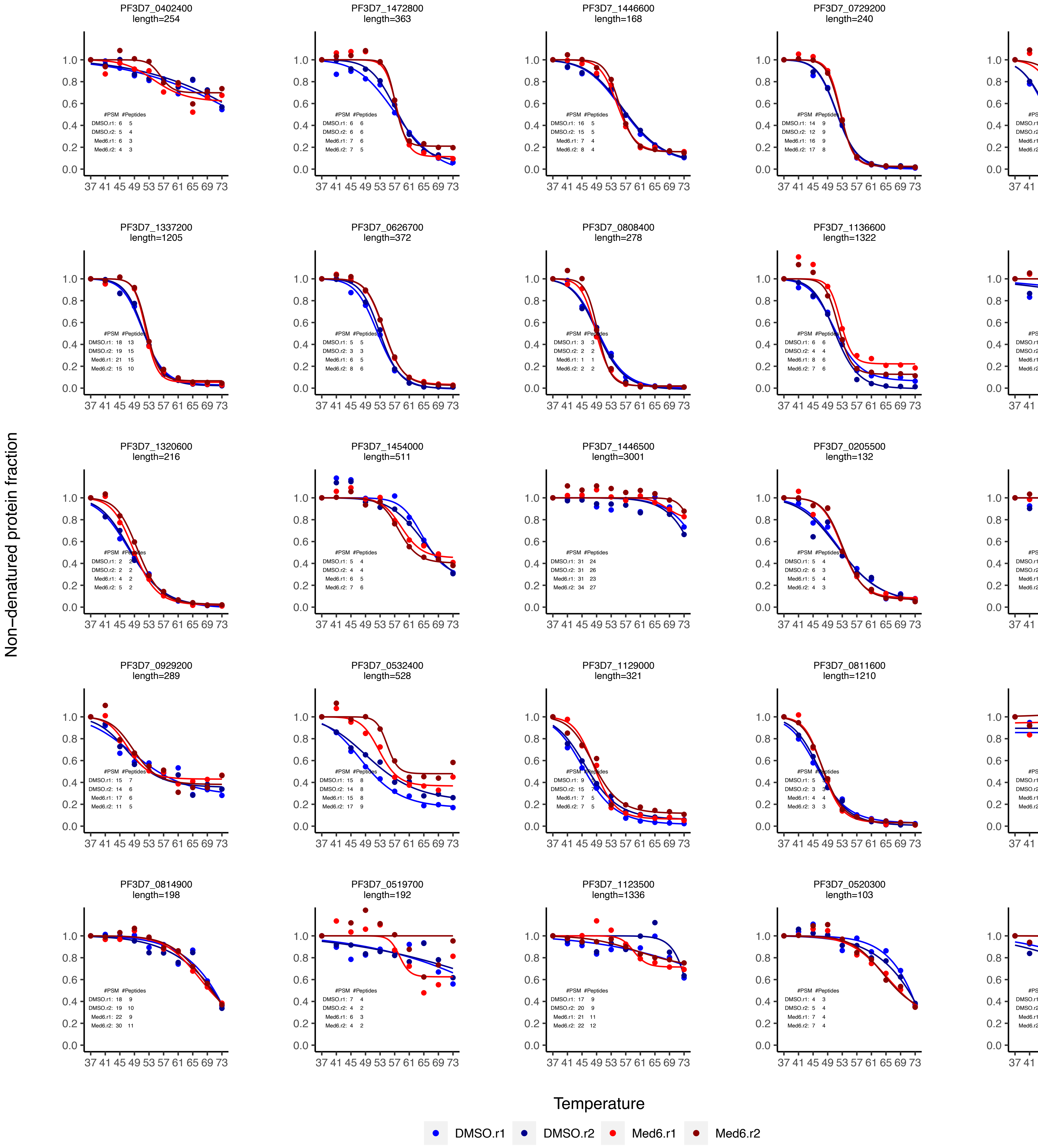

Med6 TPP data plotting_curve fitting

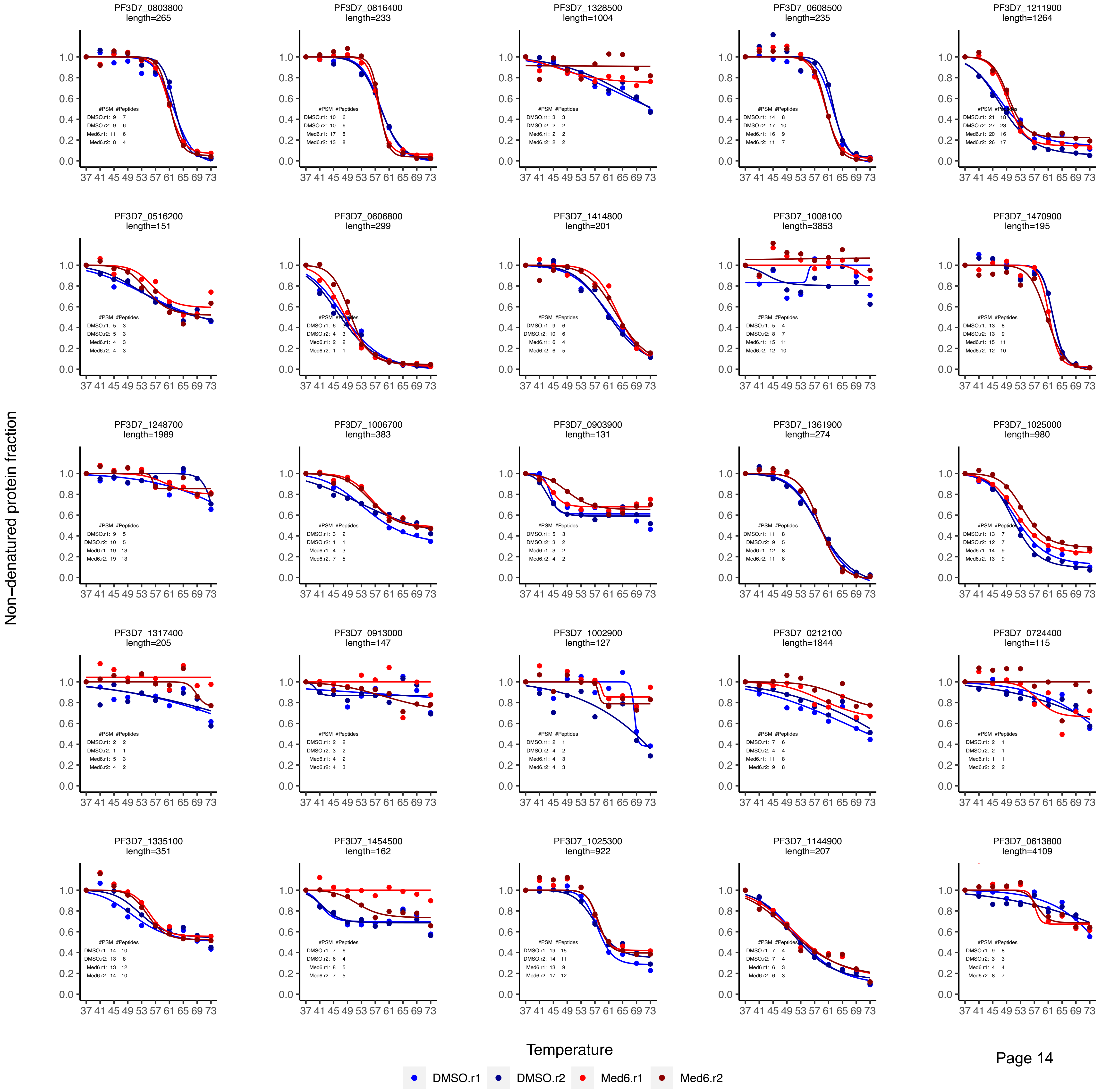

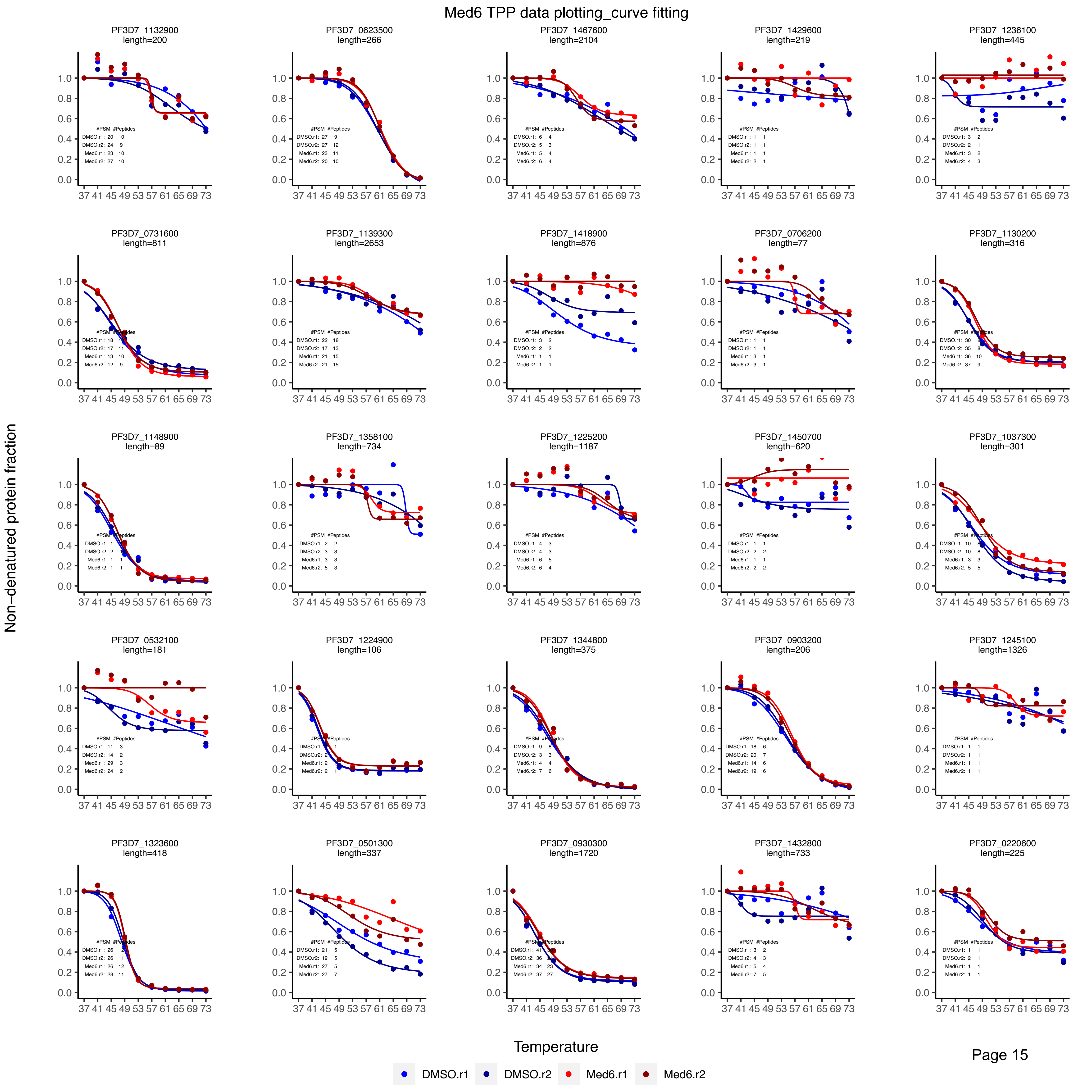
Med6 TPP data plotting_curve fitting
PF3D7_1132900
length=200
PF3D7_0623500
length=266
PF3D7_1467600
length=2104
PF3D7_1429600
length=219
PF3D7_1236100
length=445
PF3D7_0731600
length=811
PF3D7_1139300
length=2653
PF3D7_1418900
length=876
PF3D7_0706200
length=77
PF3D7_1130200
length=316
PF3D7_1148900
length=89
PF3D7_1358100
length=734
PF3D7_1225200
length=1187
PF3D7_1450700
length=620
PF3D7_1037300
length=301
PF3D7_0532100
length=181
PF3D7_1224900
length=106
PF3D7_1344800
length=375
PF3D7_0903200
length=206
PF3D7_1245100
length=1326
PF3D7_1323600
length=418
PF3D7_0501300
length=337
PF3D7_0930300
length=1720
PF3D7_1432800
length=733
PF3D7_0220600
length=225
Non−denatured protein fraction
Temperature
DMSO.r1
DMSO.r2
Med6.r1
Med6.r2
Page 15

Med6 TPP data plotting_curve fitting

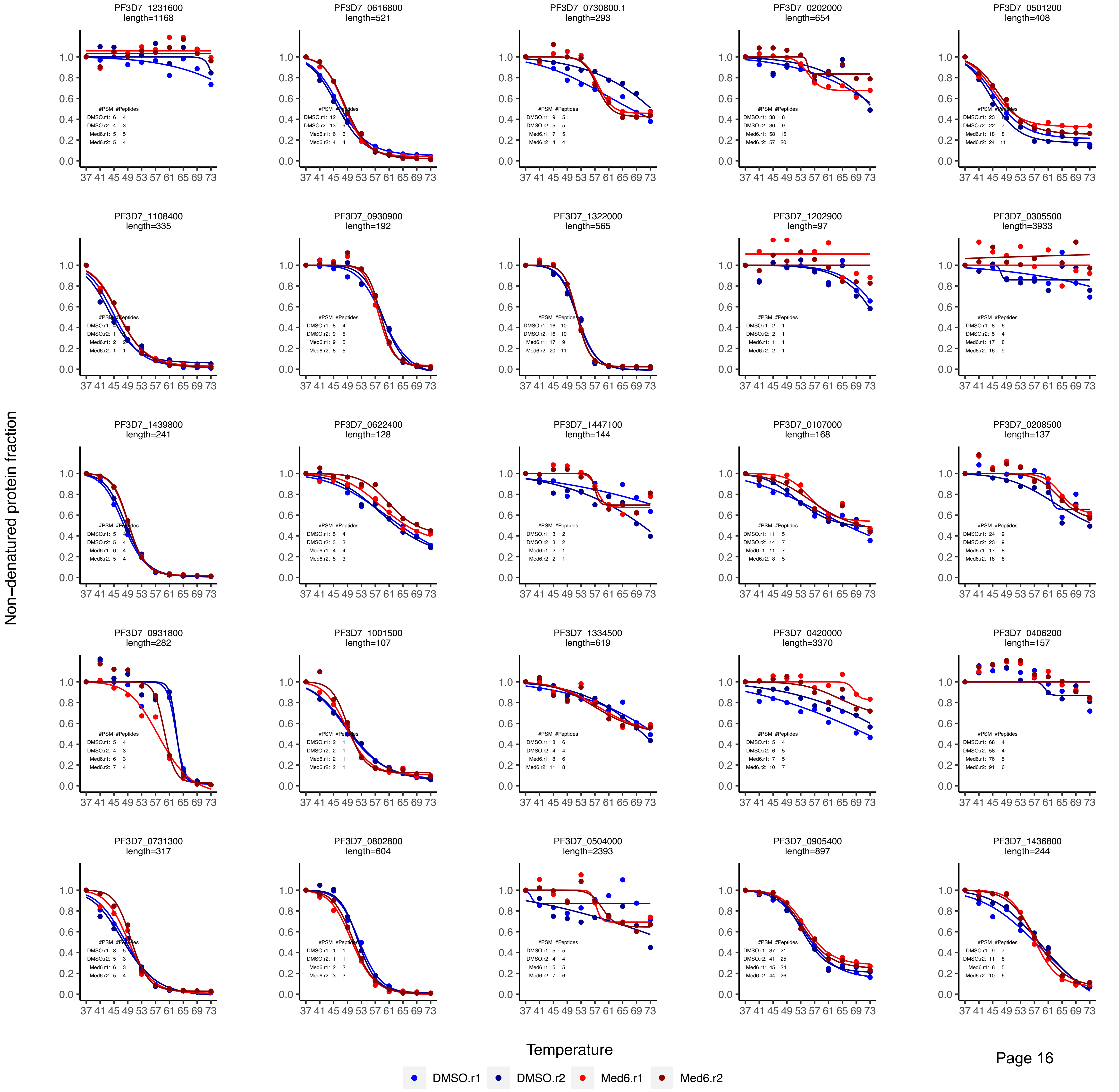

Med6 TPP data plotting_curve fitting

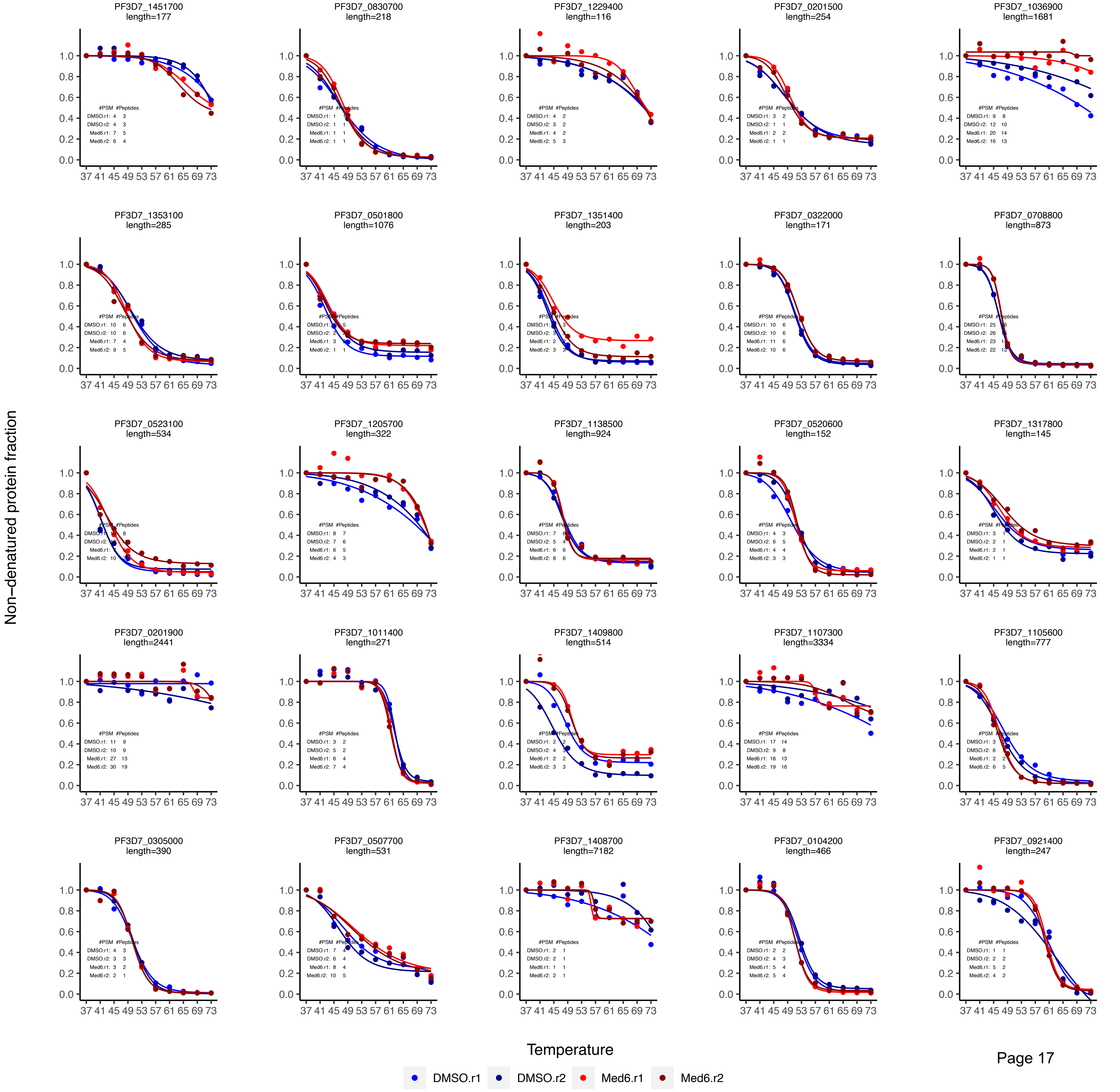

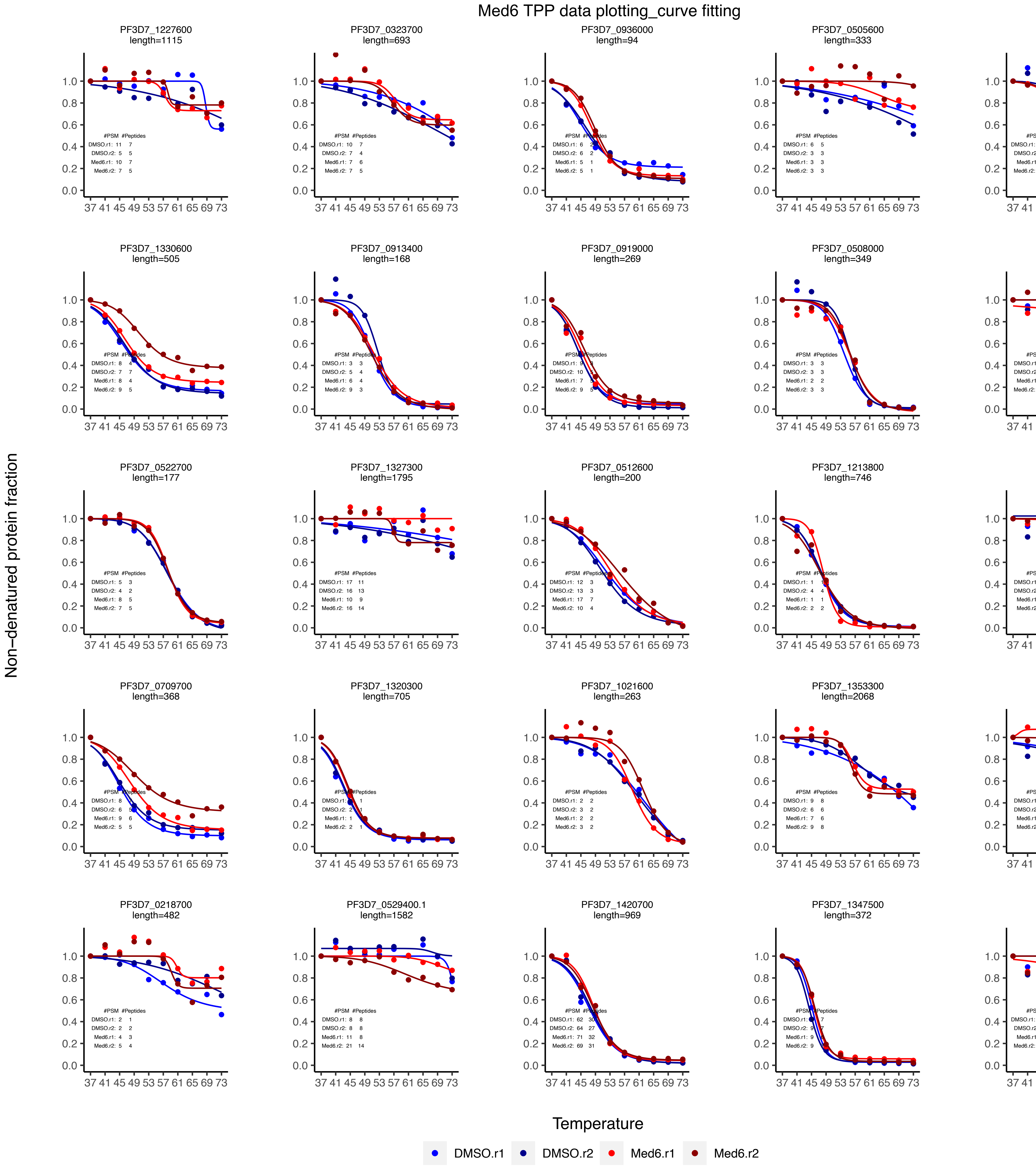
Med6 TPP data plotting_curve fitting
PF3D7_1227600 length=1115
PF3D7_0323700 length=693
PF3D7_0936000 length=94
PF3D7_0505600 length=333
PF3D7_1020900 length=181
PF3D7_1330600 length=505
PF3D7_0913400 length=168
PF3D7_0919000 length=269
PF3D7_0508000 length=349
PF3D7_1322200 length=3438
PF3D7_0522700 length=177
PF3D7_1327300 length=1795
PF3D7_0512600 length=200
PF3D7_1213800 length=746
PF3D7_0501400 length=1720
PF3D7_0709700 length=368
PF3D7_1320300 length=705
PF3D7_1021600 length=263
PF3D7_1353300 length=2068
PF3D7_0104300 length=3499
PF3D7_0218700 length=482
PF3D7_0529400.1 length=1582
PF3D7_1420700 length=969
PF3D7_1347500 length=372
PF3D7_1229300 length=990
Non−denatured protein fraction
Temperature
DMSO.r1
DMSO.r2
Med6.r1
Med6.r2

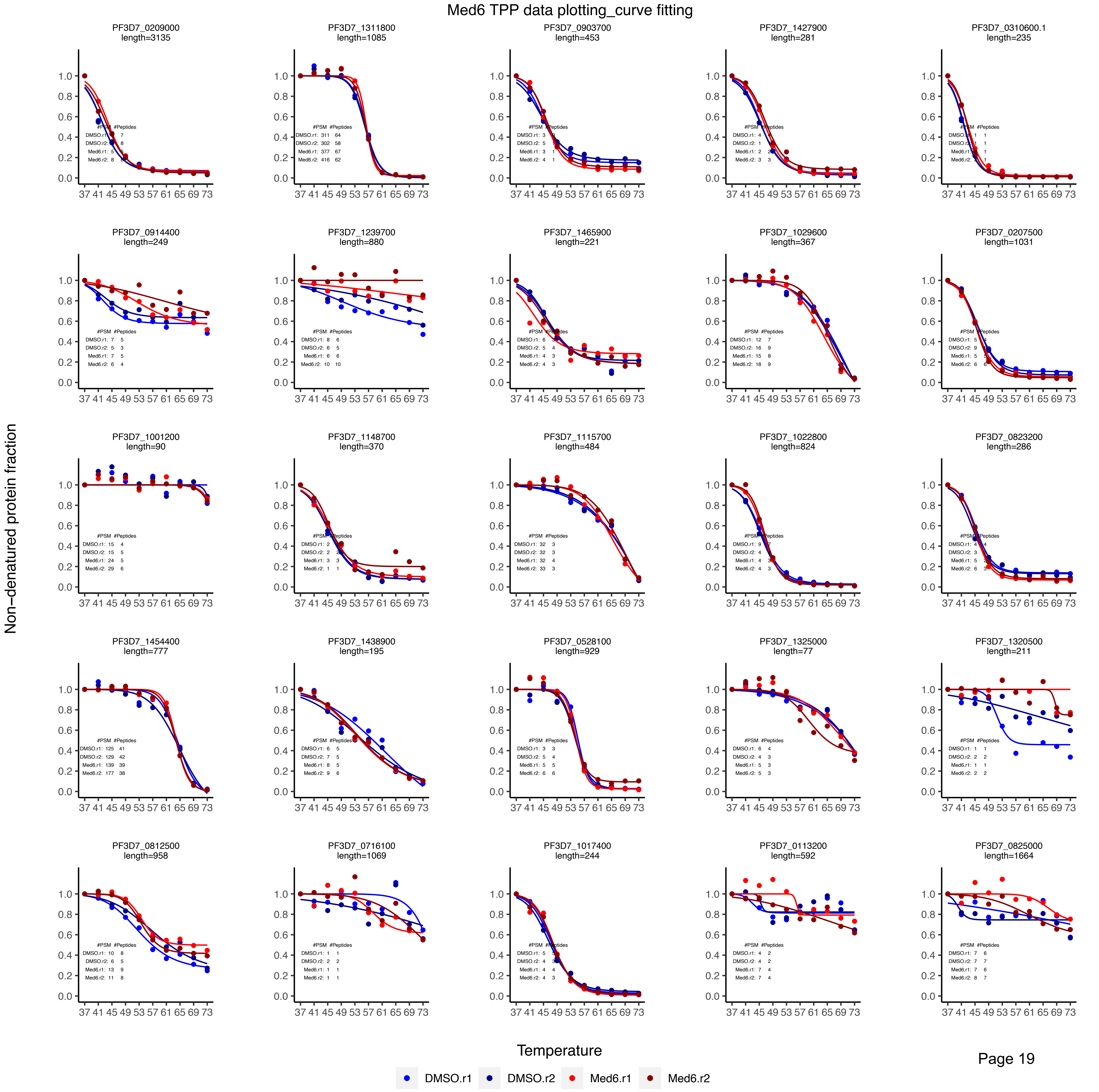
Med6 TPP data plotting_curve fitting
PF3D7_0209000
length=3135
PF3D7_1311800
length=1085
PF3D7_0903700
length=453
PF3D7_1427900
length=281
PF3D7_0310600.1
length=235
PF3D7_0914400
length=249
PF3D7_1239700
length=880
PF3D7_1465900
length=221
PF3D7_1029600
length=367
PF3D7_0207500
length=1031
PF3D7_1001200
length=90
PF3D7_1148700
length=370
PF3D7_1115700
length=484
PF3D7_1022800
length=824
PF3D7_0823200
length=286
PF3D7_1454400
length=777
PF3D7_1438900
length=195
PF3D7_0528100
length=929
PF3D7_1325000
length=77
PF3D7_1320500
length=211
PF3D7_0812500
length=958
PF3D7_0716100
length=1069
PF3D7_1017400
length=244
PF3D7_0113200
length=592
PF3D7_0825000
length=1664
Non−denatured protein fraction
Temperature
DMSO.r1
DMSO.r2
Med6.r1
Med6.r2
Page 19

Med6 TPP data plotting_curve fitting

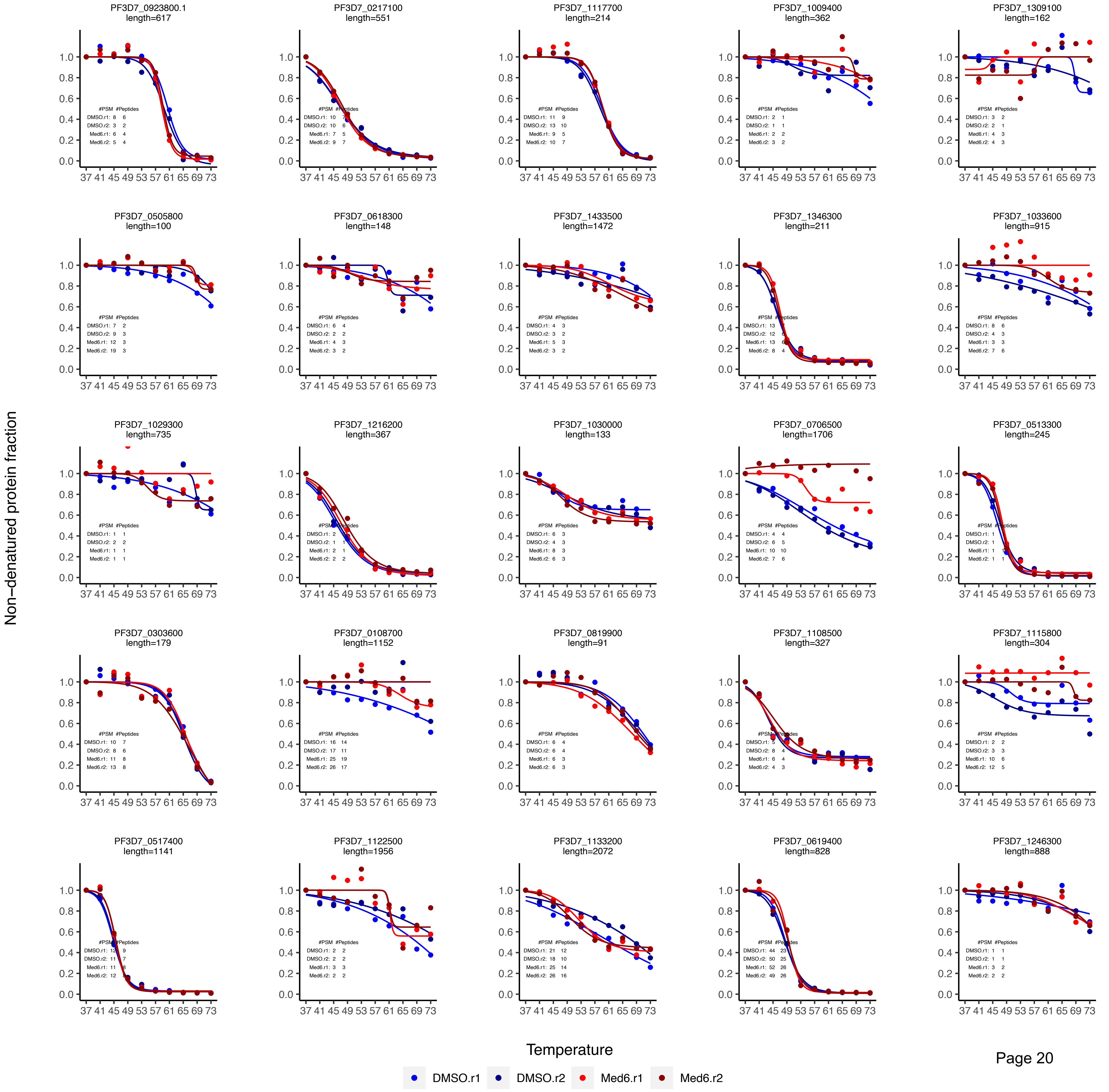

Med6 TPP data plotting_curve fitting

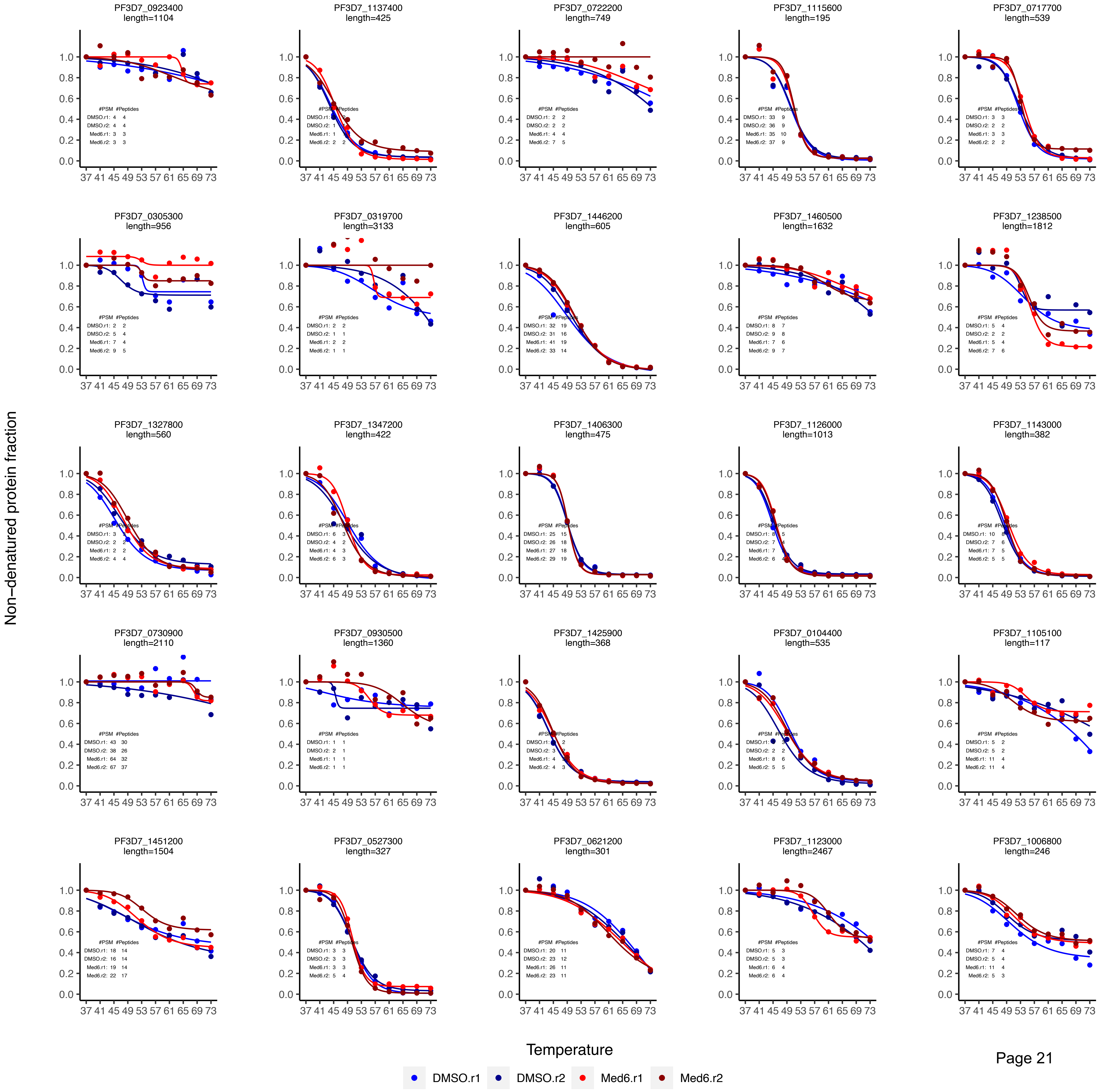

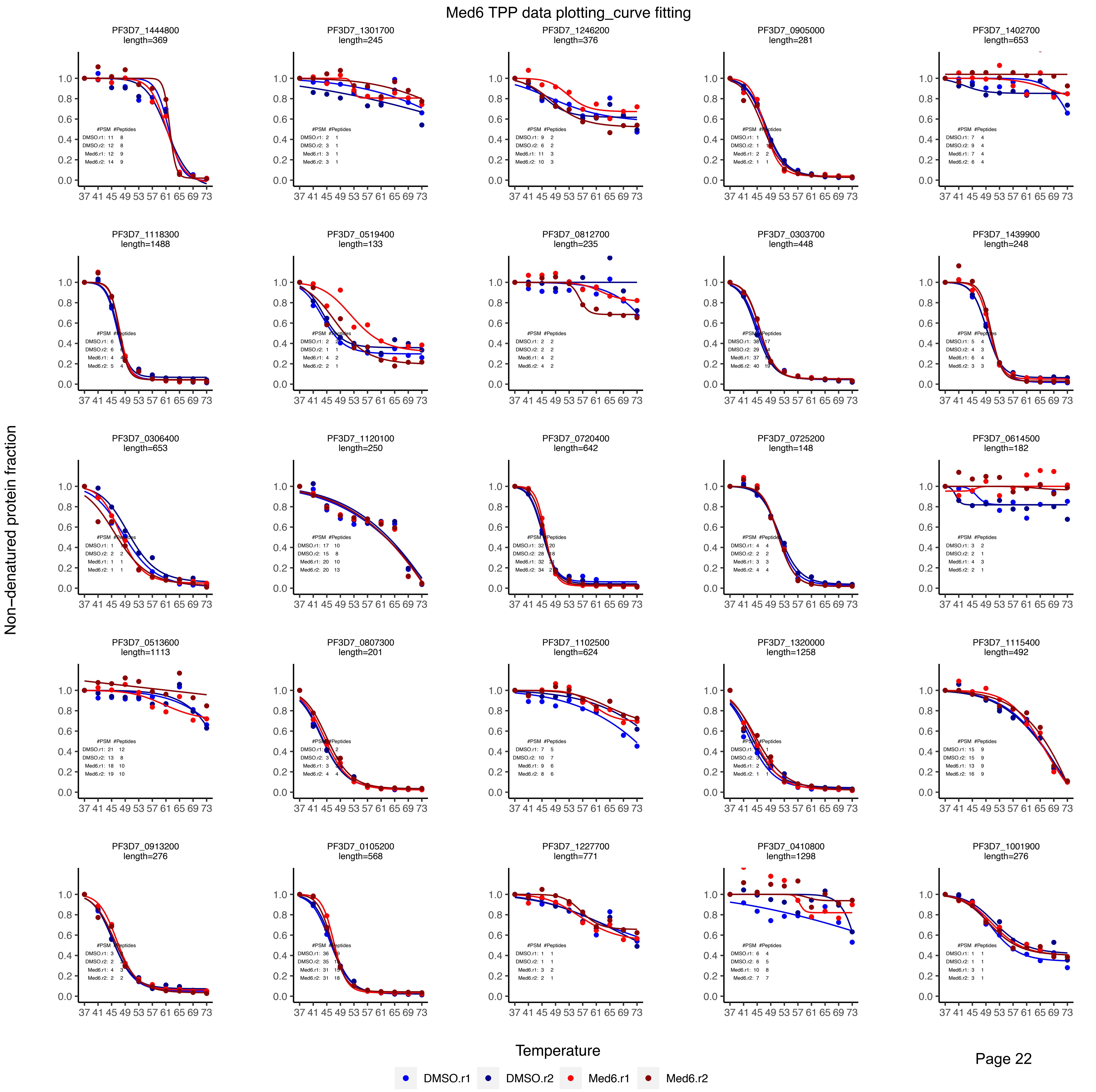
Med6 TPP data plotting_curve fitting
PF3D7_1444800
length=369
PF3D7_1301700
length=245
PF3D7_1246200
length=376
PF3D7_0905000
length=281
PF3D7_1402700
length=653
PF3D7_1118300
length=1488
PF3D7_0519400
length=133
PF3D7_0812700
length=235
PF3D7_0303700
length=448
PF3D7_1439900
length=248
PF3D7_0306400
length=653
PF3D7_1120100
length=250
PF3D7_0720400
length=642
PF3D7_0725200
length=148
PF3D7_0614500
length=182
PF3D7_0513600
length=1113
PF3D7_0807300
length=201
PF3D7_1102500
length=624
PF3D7_1320000
length=1258
PF3D7_1115400
length=492
PF3D7_0913200
length=276
PF3D7_0105200
length=568
PF3D7_1227700
length=771
PF3D7_0410800
length=1298
PF3D7_1001900
length=276
Non-denatured protein fraction
Temperature
DMSO.r1
DMSO.r2
Med6.r1
Med6.r2

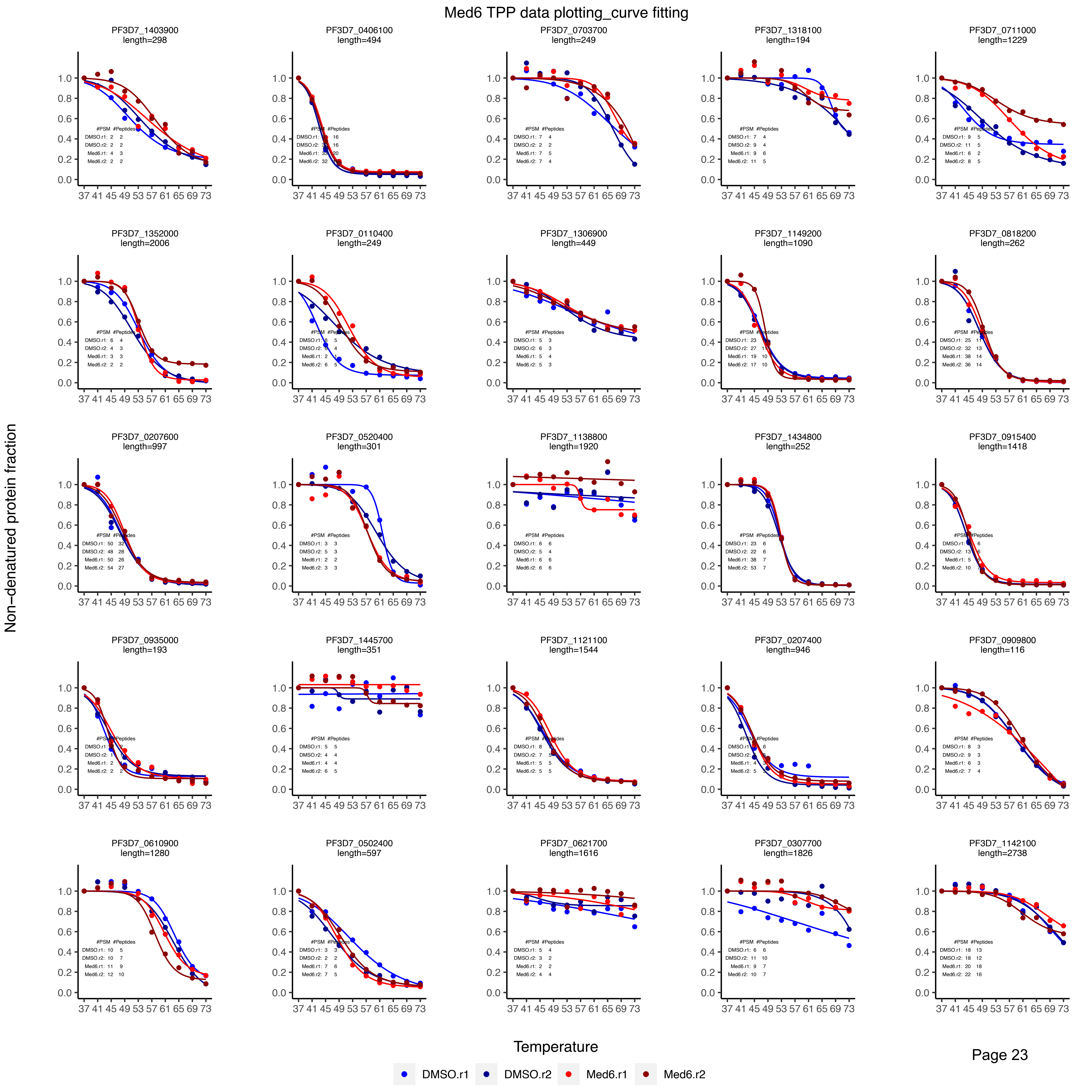
Med6 TPP data plotting_curve fitting
PF3D7_1403900
length=298
PF3D7_0406100
length=494
PF3D7_0703700
length=249
PF3D7_1318100
length=194
PF3D7_0711000
length=1229
PF3D7_1352000
length=2006
PF3D7_0110400
length=249
PF3D7_1306900
length=449
PF3D7_1149200
length=1090
PF3D7_0818200
length=262
PF3D7_0207600
length=997
PF3D7_0520400
length=301
PF3D7_1138800
length=1920
PF3D7_1434800
length=252
PF3D7_0915400
length=1418
PF3D7_0935000
length=193
PF3D7_1445700
length=351
PF3D7_1121100
length=1544
PF3D7_0207400
length=946
PF3D7_0909800
length=116
PF3D7_0610900
length=1280
PF3D7_0502400
length=597
PF3D7_0621700
length=1616
PF3D7_0307700
length=1826
PF3D7_1142100
length=2738
Non-denatured protein fraction
Temperature
DMSO.r1
DMSO.r2
Med6.r1
Med6.r2
Page 23

Med6 TPP data plotting_curve fitting

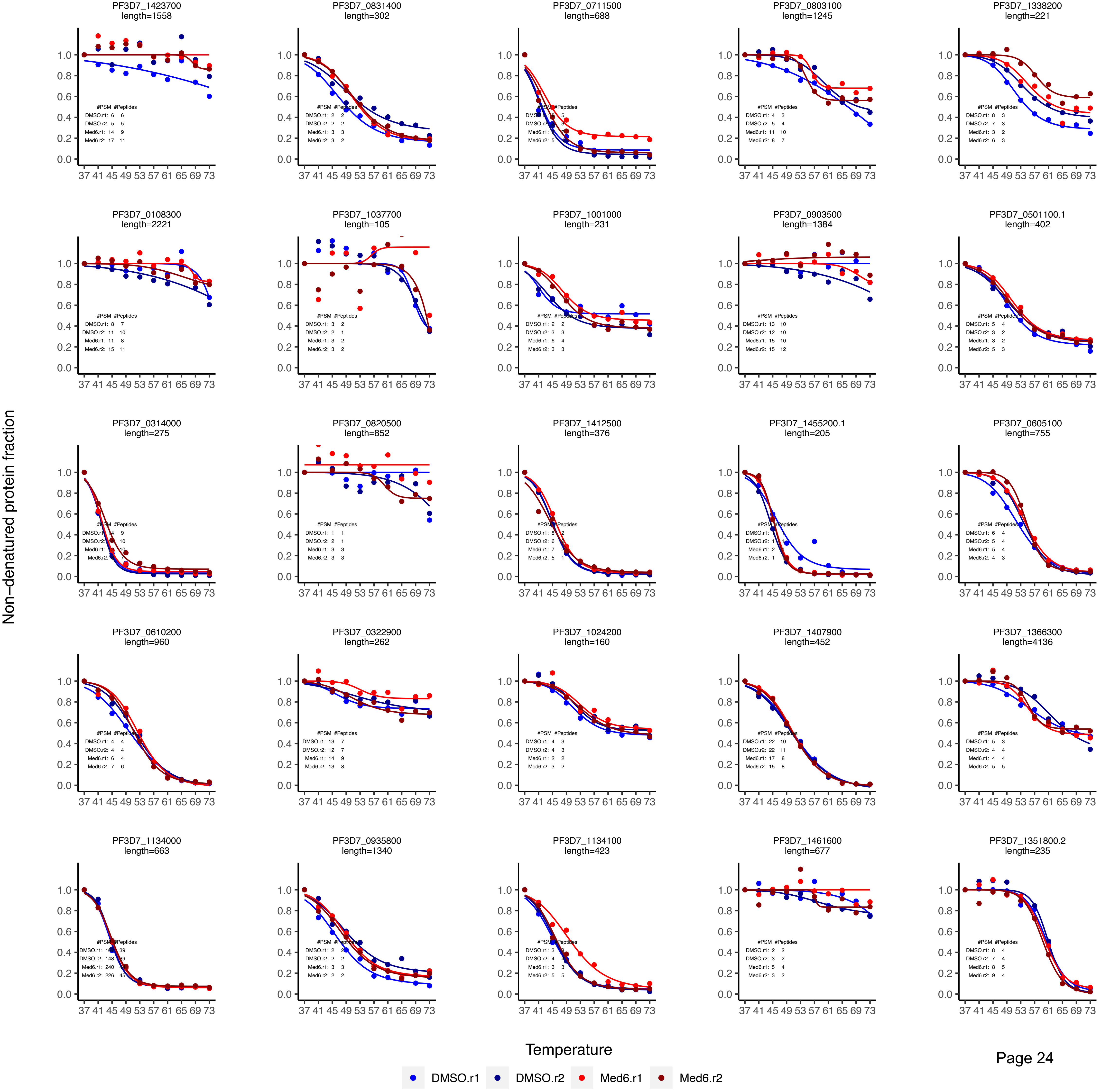

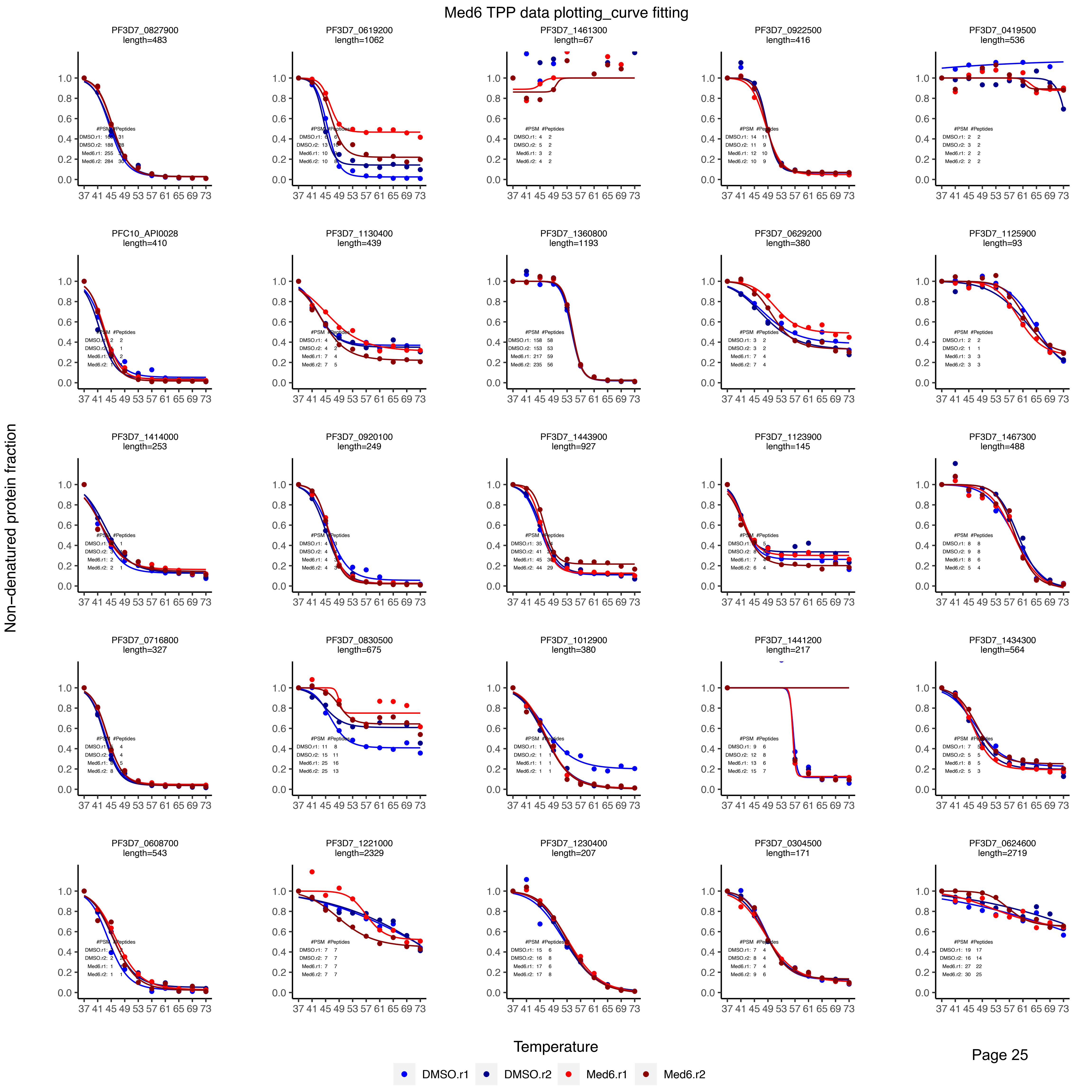
Med6 TPP data plotting_curve fitting
PF3D7_0827900
length=483
PF3D7_0619200
length=1062
PF3D7_1461300
length=67
PF3D7_0922500
length=416
PF3D7_0419500
length=536
PFC10_API0028
length=410
PF3D7_1130400
length=439
PF3D7_1360800
length=1193
PF3D7_0629200
length=380
PF3D7_1125900
length=93
PF3D7_1414000
length=253
PF3D7_0920100
length=249
PF3D7_1443900
length=927
PF3D7_1123900
length=145
PF3D7_1467300
length=488
PF3D7_0716800
length=327
PF3D7_0830500
length=675
PF3D7_1012900
length=380
PF3D7_1441200
length=217
PF3D7_1434300
length=564
PF3D7_0608700
length=543
PF3D7_1221000
length=2329
PF3D7_1230400
length=207
PF3D7_0304500
length=171
PF3D7_0624600
length=2719
Non−denatured protein fraction
Temperature
DMSO.r1
DMSO.r2
Med6.r1
Med6.r2
Page 25

Med6 TPP data plotting_curve fitting

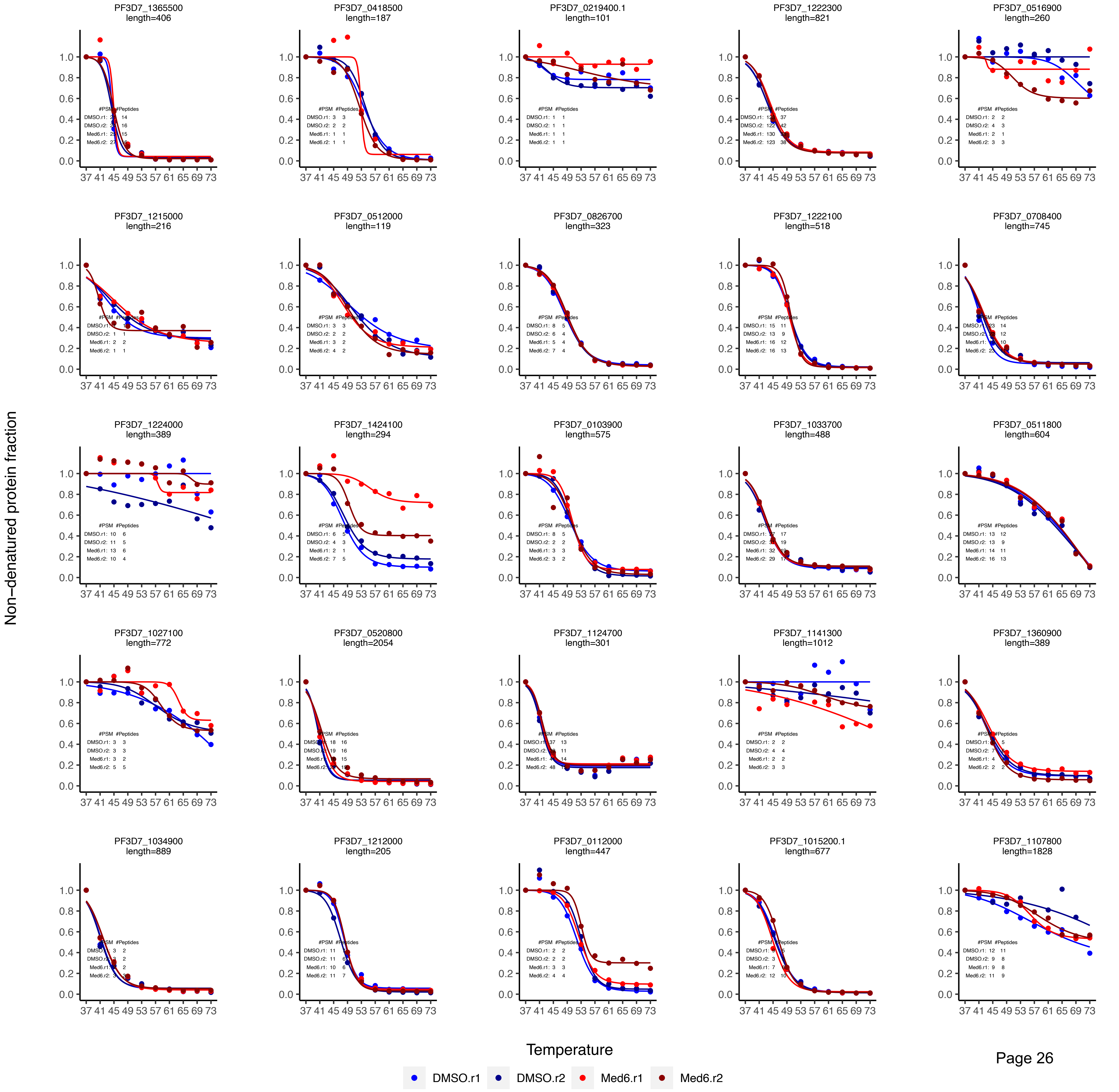

Med6 TPP data plotting_curve fitting

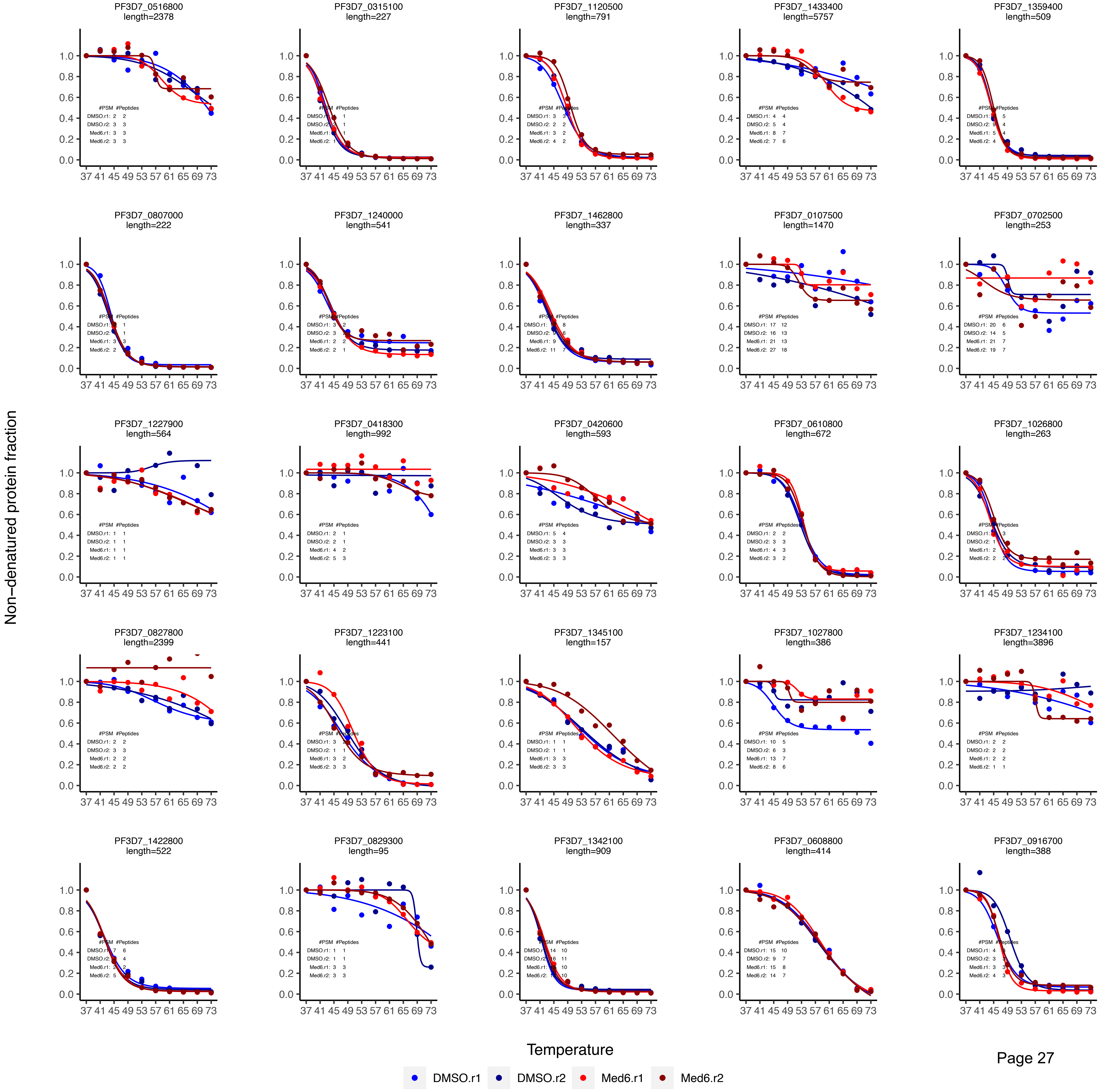

Med6 TPP data plotting_curve fitting

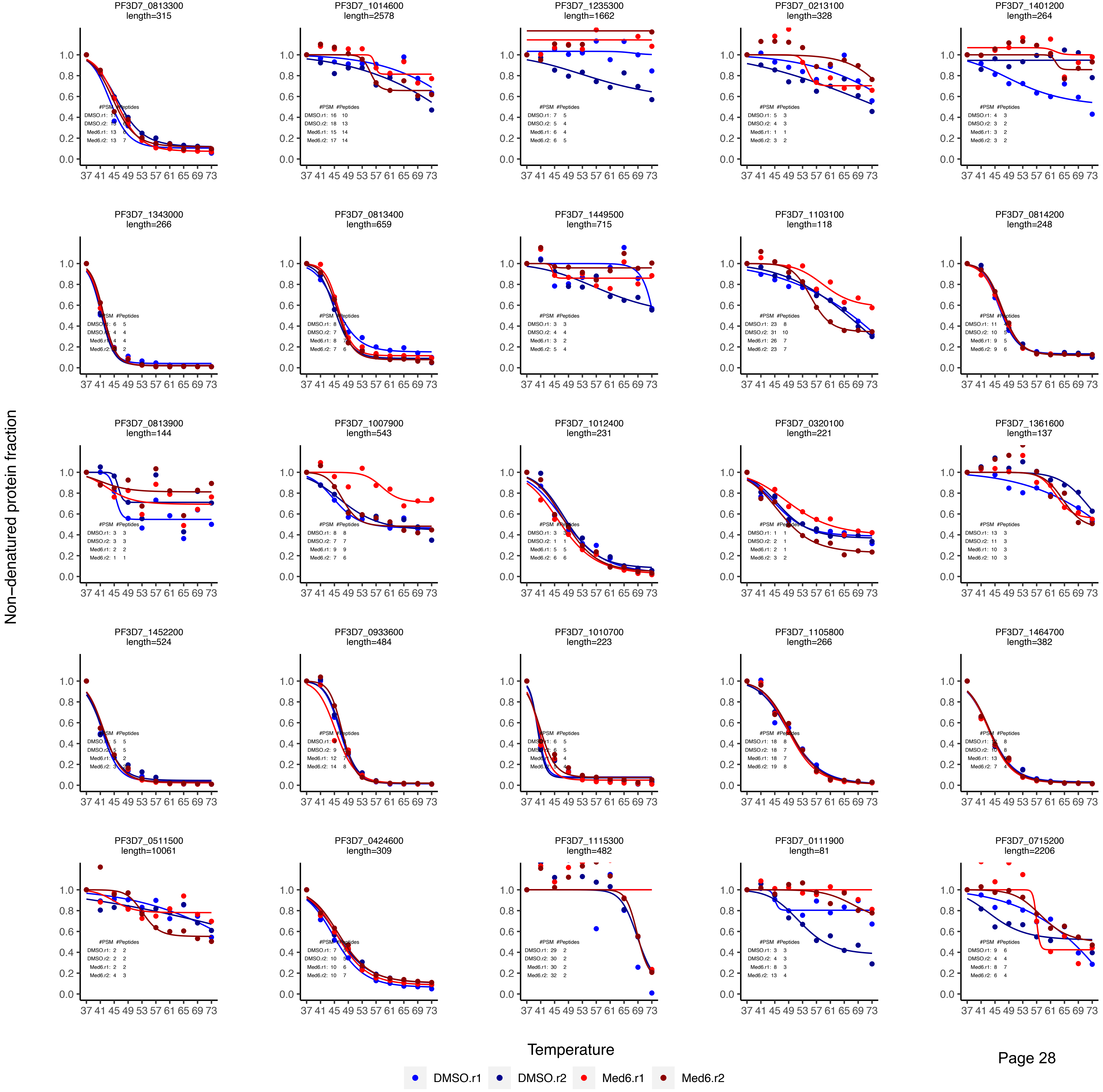

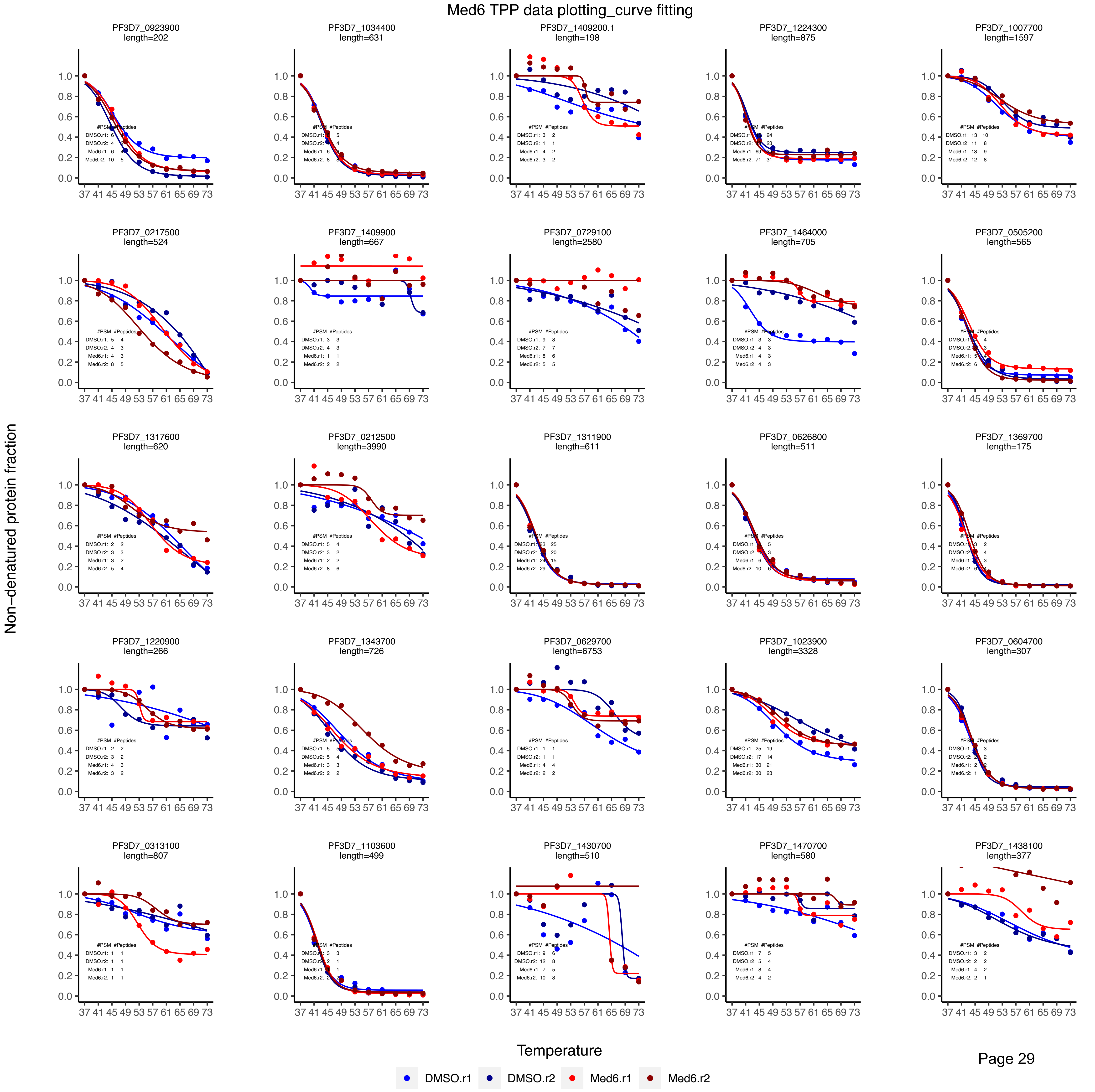
Med6 TPP data plotting_curve fitting
PF3D7_0923900
length=202
PF3D7_1034400
length=631
PF3D7_1409200.1
length=198
PF3D7_1224300
length=875
PF3D7_1007700
length=1597
PF3D7_0217500
length=524
PF3D7_1409900
length=667
PF3D7_0729100
length=2580
PF3D7_1464000
length=705
PF3D7_0505200
length=565
PF3D7_1317600
length=620
PF3D7_0212500
length=3990
PF3D7_1311900
length=611
PF3D7_0626800
length=511
PF3D7_1369700
length=175
PF3D7_1220900
length=266
PF3D7_1343700
length=726
PF3D7_0629700
length=6753
PF3D7_1023900
length=3328
PF3D7_0604700
length=307
PF3D7_0313100
length=807
PF3D7_1103600
length=499
PF3D7_1430700
length=510
PF3D7_1470700
length=580
PF3D7_1438100
length=377
Non−denatured protein fraction
Temperature
DMSO.r1
DMSO.r2
Med6.r1
Med6.r2
Page 29

Med6 TPP data plotting_curve fitting

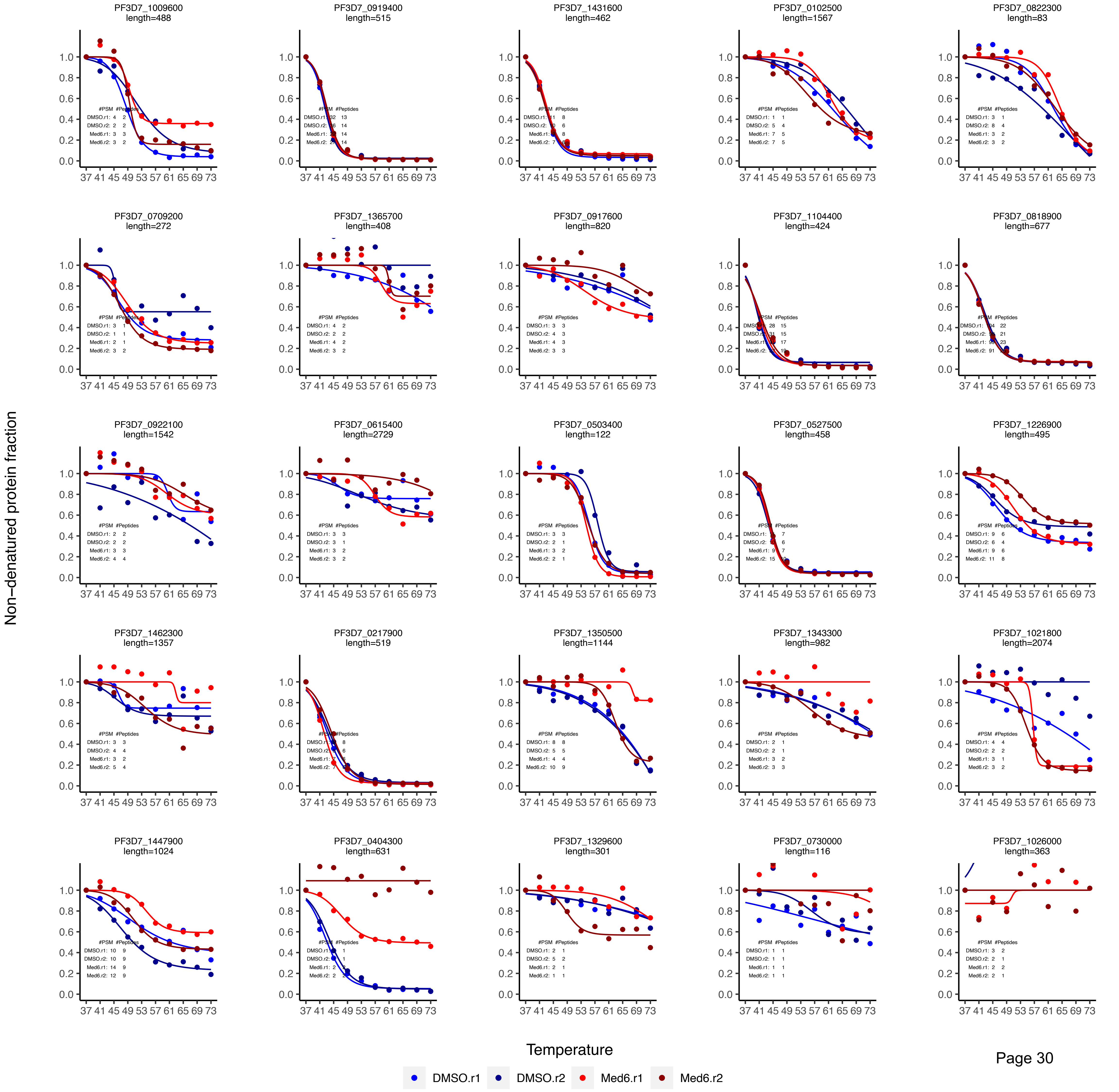

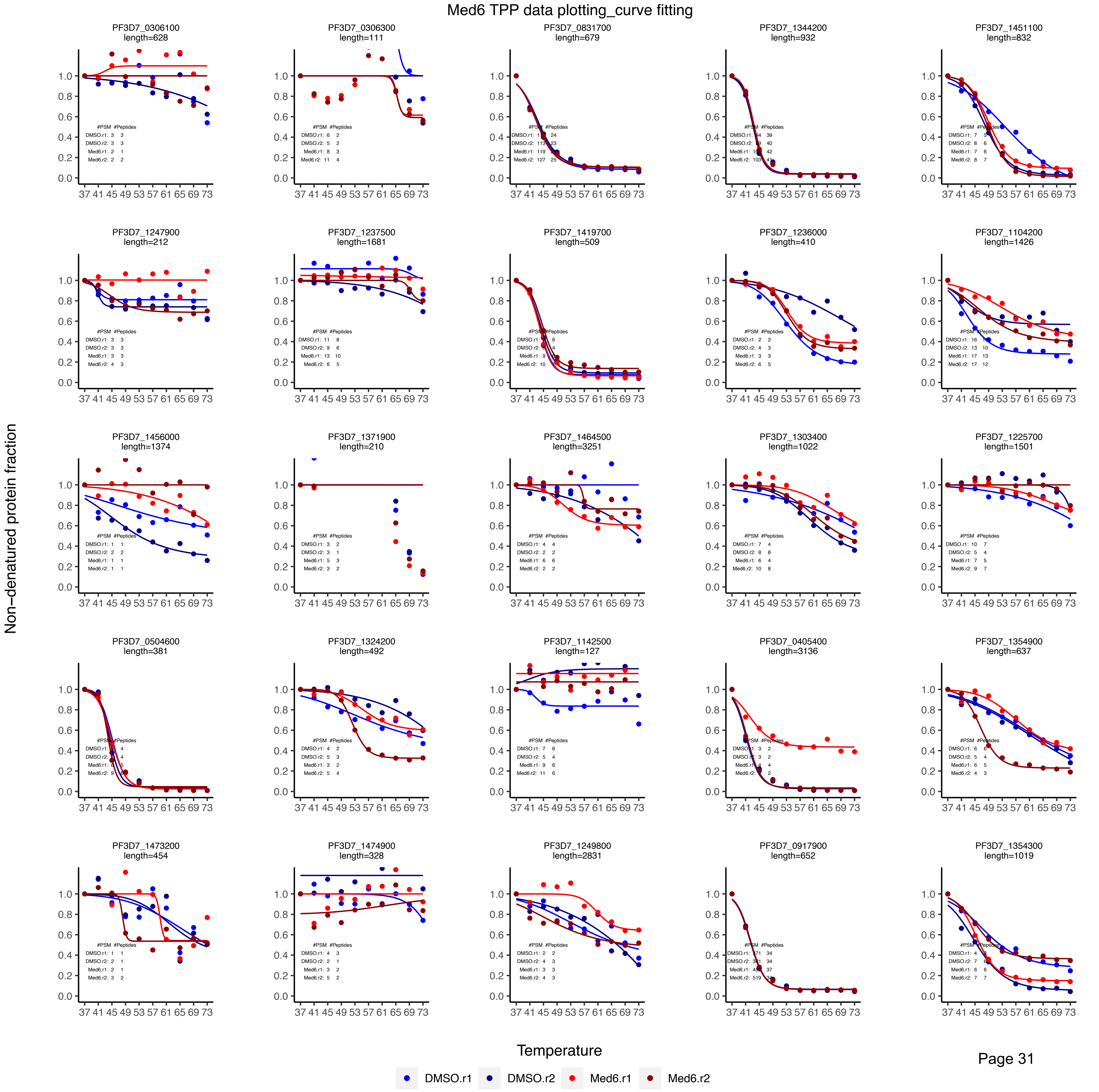
Med6 TPP data plotting_curve fitting
PF3D7_0306100 length=628
PF3D7_0306300 length=111
PF3D7_0831700 length=679
PF3D7_1344200 length=932
PF3D7_1451100 length=832
PF3D7_1247900 length=212
PF3D7_1237500 length=1681
PF3D7_1419700 length=509
PF3D7_1236000 length=410
PF3D7_1104200 length=1426
PF3D7_1456000 length=1374
PF3D7_1371900 length=210
PF3D7_1464500 length=3251
PF3D7_1303400 length=1022
PF3D7_1225700 length=1501
PF3D7_0504600 length=381
PF3D7_1324200 length=492
PF3D7_1142500 length=127
PF3D7_0405400 length=3136
PF3D7_1354900 length=637
PF3D7_1473200 length=454
PF3D7_1474900 length=328
PF3D7_1249800 length=2831
PF3D7_0917900 length=652
PF3D7_1354300 length=1019
Non−denatured protein fraction
Temperature
DMSO.r1
DMSO.r2
Med6.r1
Med6.r2

Med6 TPP data plotting_curve fitting

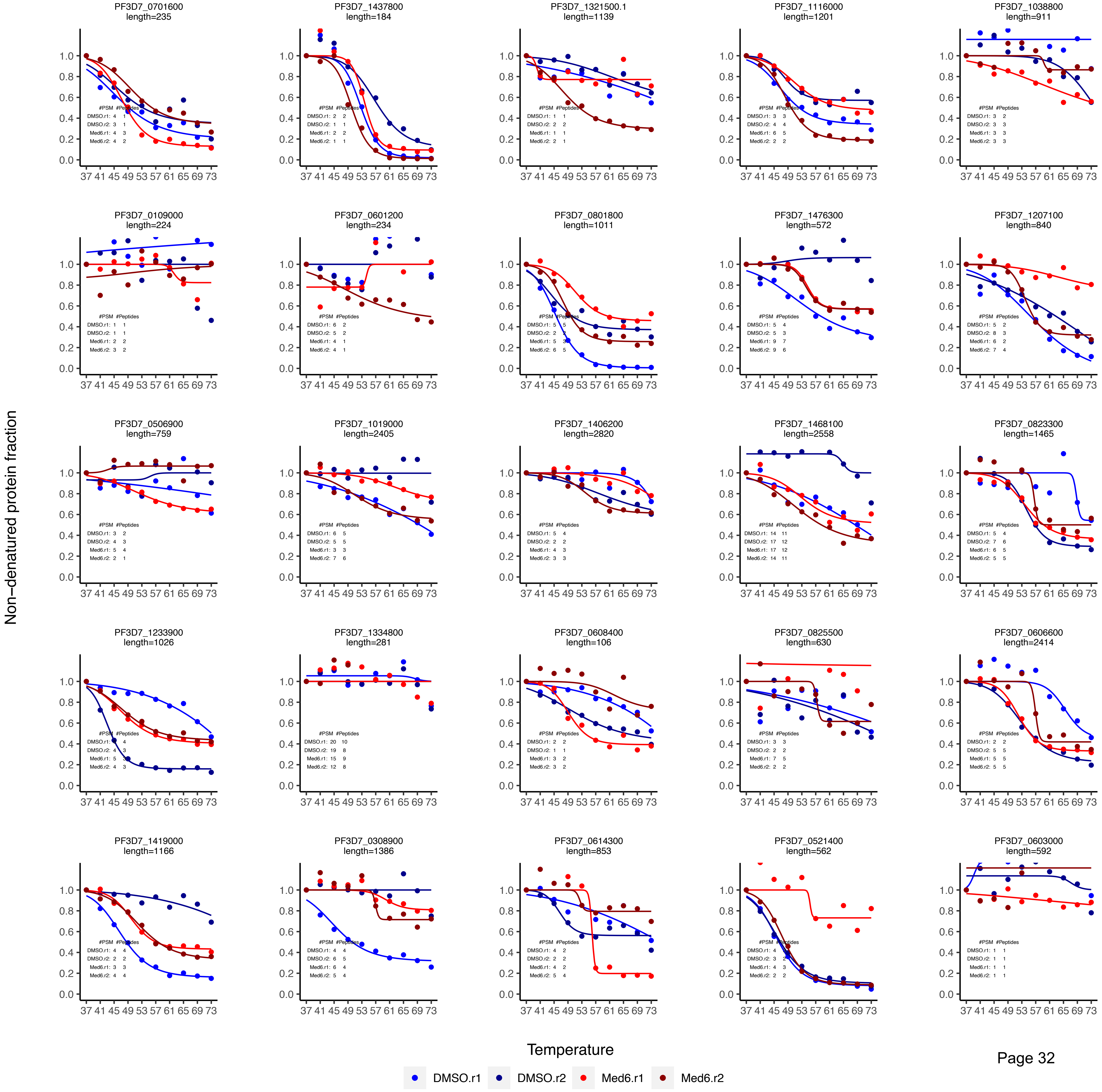

Med6 TPP data plotting_curve fitting

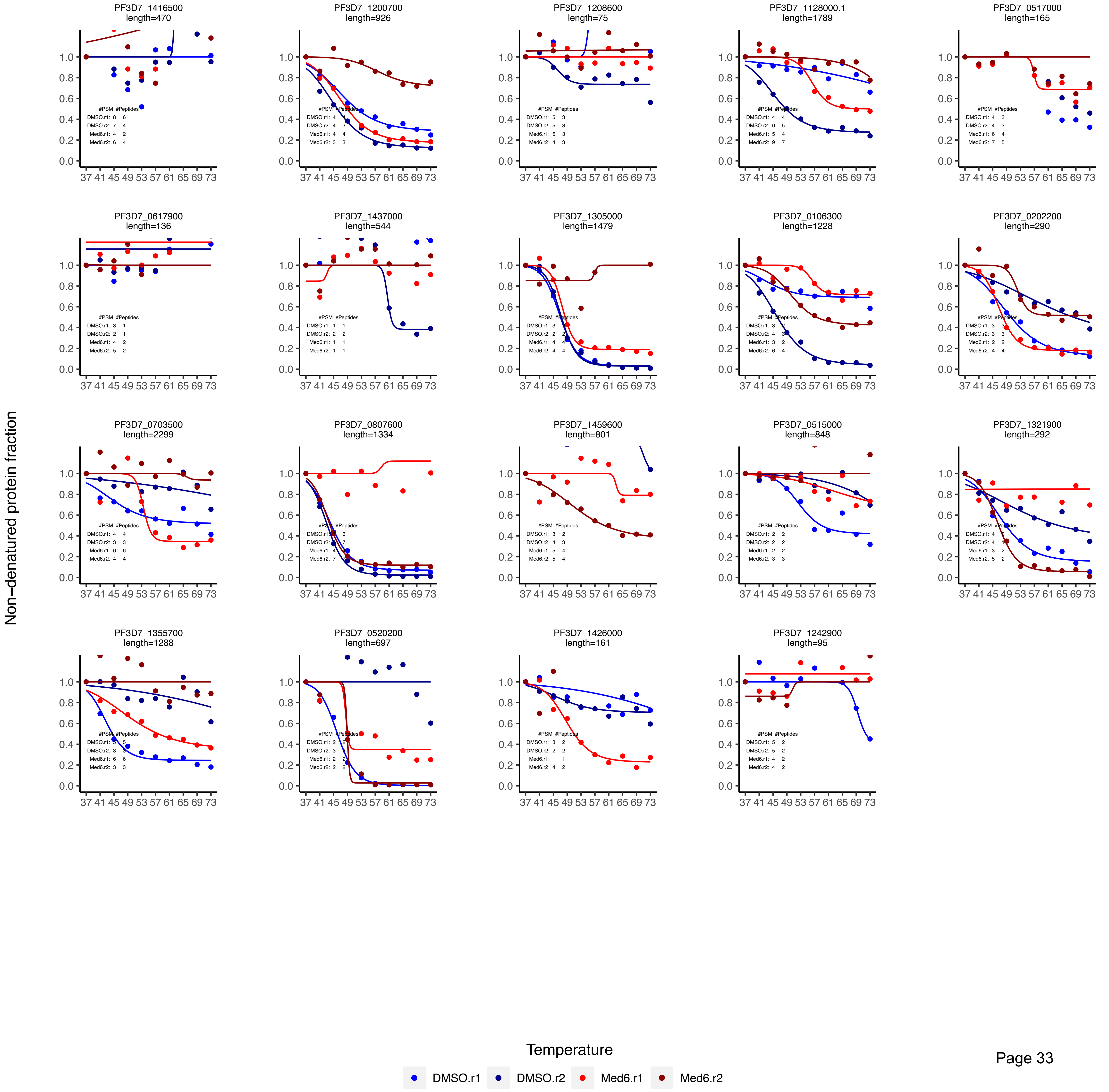

References and Notes

1. World Health Organization, *World Malaria Report 2023* (World Health Organization, 2023).

2. X. Wang, X. Zhang, H. Chen, J. Zhang, Q. Lu, W. Ruan, F. Ling, Molecular epidemiology of drug resistance genes in *Plasmodium falciparum* isolates imported from Nigeria between 2016 and 2020: Continued emergence of fully resistant *Pfdhfr-Pfdhps* alleles. *Microbiol. Spectr.* **10**, e00528-22 (2022). doi:10.1128/spectrum.00528-22 Medline

3. World Health Organization, *Global Report on Antimalarial Drug Efficacy and Drug Resistance: 2000–2010* (World Health Organization, 2010).

4. A. Uwimana, E. Legrand, B. H. Stokes, J. M. Ndikumana, M. Warsame, N. Umulisa, D. Ngamije, T. Munyaneza, J.-B. Mazarati, K. Munguti, P. Campagne, A. Criscuolo, F. Ariey, M. Murindahabi, P. Ringwald, D. A. Fidock, A. Mbituyumuremyi, D. Menard, Emergence and clonal expansion of in vitro artemisinin-resistant *Plasmodium falciparum kelch13* R561H mutant parasites in Rwanda. *Nat. Med.* **26**, 1602–1608 (2020). doi:10.1038/s41591-020-1005-2 Medline

5. A. Uwimana, N. Umulisa, M. Venkatesan, S. S. Svigel, Z. Zhou, T. Munyaneza, R. M. Habimana, A. Rucogoza, L. F. Moriarty, R. Sandford, E. Piercefield, I. Goldman, B. Ezema, E. Talundzic, M. A. Pacheco, A. A. Escalante, D. Ngamije, J. N. Mangala, M. Kabera, K. Munguti, M. Murindahabi, W. Brieger, C. Musanabaganwa, L. Mutesa, V. Udhayakumar, A. Mbituyumuremyi, E. S. Halsey, N. W. Lucchi, Association of *Plasmodium falciparum kelch13* R561H genotypes with delayed parasite clearance in Rwanda: An open-label, single-arm, multicentre, therapeutic efficacy study. *Lancet Infect. Dis.* **21**, 1120–1128 (2021). doi:10.1016/S1473-3099(21)00142-0 Medline

6. N. Noreen, A. Ullah, S. M. Salman, Y. Mabkhot, A. Alsayari, S. L. Badshah, New insights into the spread of resistance to artemisinin and its analogues. *J. Glob. Antimicrob. Resist.* **27**, 142–149 (2021). doi:10.1016/j.jgar.2021.09.001 Medline

7. A. M. Samuels, D. Ansong, S. K. Kariuki, S. Adjei, A. Bollaerts, C. Ockenhouse, N. Westercamp, C. K. Lee, L. Schuerman, D. K. Bii, L. Osei-Tutu, M. Oneko, M. Lievens, M. A. Attobrah Sarfo, C. Atieno, D. Morelle, A. Bakari, T. Sang, E. Jongert, M. F. Kotoh-Mortty, K. Otieno, F. Roman, P. B. Y. Buabeng, Y. Ntiamoah, O. Ofori-Anyinam, T. Agbenyega; RTS,S study group, Efficacy of RTS,S/AS01$_E$ malaria vaccine administered according to different full, fractional, and delayed third or early fourth dose regimens in children aged 5–17 months in Ghana and Kenya: An open-label, phase 2b, randomised controlled trial. *Lancet Infect. Dis.* **22**, 1329–1342 (2022). doi:10.1016/S1473-3099(22)00273-0 Medline

8. K. E. Ward, D. A. Fidock, J. L. Bridgford, *Plasmodium falciparum* resistance to artemisinin-based combination therapies. *Curr. Opin. Microbiol.* **69**, 102193 (2022). doi:10.1016/j.mib.2022.102193 Medline

9. C. W. J. Chang, A. Patra, D. M. Roll, P. J. Scheuer, Kalihinol-A, a highly functionalized diisocyano diterpenoid antibiotic from a sponge. *J. Am. Chem. Soc.* **106**, 4644–4646 (1984). doi:10.1021/ja00328a073

10. L. H. Yang, O. O. Lee, T. Jin, X. C. Li, P. Y. Qian, Antifouling properties of 10β-formamidokalihinol-A and kalihinol A isolated from the marine sponge *Acanthella cavernosa*. *Biofouling* **22**, 23–32 (2006). doi:10.1080/08927010500498623 Medline

11. H. Miyaoka, Y. Abe, E. Kawashima, Synthesis of marine diterpene isocyanide (−)-kalihinol Y and diterpene isothiocyanate (−)-10-epi-kalihinol I. *Chem. Pharm. Bull. (Tokyo)* **60**, 1224–1226 (2012). doi:10.1248/cpb.c12-00485 Medline

12. D. Wolf, F. J. Schmitz, New diterpene isonitriles from the sponge *Phakellia pulcherrima*. *J. Nat. Prod.* **61**, 1524–1527 (1998). doi:10.1021/np980240g Medline

13. M. E. Daub, J. Prudhomme, K. Le Roch, C. D. Vanderwal, Synthesis and potent antimalarial activity of kalihinol B. *J. Am. Chem. Soc.* **137**, 4912–4915 (2015). doi:10.1021/jacs.5b01152 Medline

14. M. E. Daub, J. Prudhomme, C. Ben Mamoun, K. G. Le Roch, C. D. Vanderwal, Antimalarial properties of simplified kalihinol analogues. *ACS Med. Chem. Lett.* **8**, 355–360 (2017). doi:10.1021/acsmedchemlett.7b00013 Medline

15. A. D. Wright, G. M. König, C. K. Angerhofer, P. Greenidge, A. Linden, R. Desqueyroux-Faúndez, Antimalarial activity: The search for marine-derived natural products with selective antimalarial activity. *J. Nat. Prod.* **59**, 710–716 (1996). doi:10.1021/np9602325 Medline

16. M. J. Schnermann, R. A. Shenvi, Syntheses and biological studies of marine terpenoids derived from inorganic cyanide. *Nat. Prod. Rep.* **32**, 543–577 (2015). doi:10.1039/C4NP00109E Medline

17. A. D. Wright, H. Wang, M. Gurrath, G. M. König, G. Kocak, G. Neumann, P. Loria, M. Foley, L. Tilley, Inhibition of heme detoxification processes underlies the antimalarial activity of terpene isonitrile compounds from marine sponges. *J. Med. Chem.* **44**, 873–885 (2001). doi:10.1021/jm0010724 Medline

18. H. H. Lu, S. V. Pronin, Y. Antonova-Koch, S. Meister, E. A. Winzeler, R. A. Shenvi, Synthesis of (+)-7,20-diisocyanoadociane and liver-stage antiplasmodial activity of the isocyanoterpene class. *J. Am. Chem. Soc.* **138**, 7268–7271 (2016). doi:10.1021/jacs.6b03899 Medline

19. D. F. Taber, B. P. Gunn, Control elements in the intramolecular Diels-Alder reaction. Synthesis of (±)-torreyol. *J. Am. Chem. Soc.* **101**, 3992–3993 (1979). doi:10.1021/ja00508a061

20. S. V. Pronin, C. A. Reiher, R. A. Shenvi, Stereoinversion of tertiary alcohols to tertiary-alkyl isonitriles and amines. *Nature* **501**, 195–199 (2013). doi:10.1038/nature12472 Medline

21. E. L. Dahl, P. J. Rosenthal, Multiple antibiotics exert delayed effects against the *Plasmodium falciparum* apicoplast. *Antimicrob. Agents Chemother.* **51**, 3485–3490 (2007). doi:10.1128/AAC.00527-07 Medline

22. K. Kennedy, S. A. Cobbold, E. Hanssen, J. Birnbaum, N. J. Spillman, E. McHugh, H. Brown, L. Tilley, T. Spielmann, M. J. McConville, S. A. Ralph, Delayed death in the malaria parasite *Plasmodium falciparum* is caused by disruption of prenylation-

dependent intracellular trafficking. *PLOS Biol.* **17**, e3000376 (2019). doi:10.1371/journal.pbio.3000376 Medline

23. S. C. Nair, C. F. Brooks, C. D. Goodman, A. Sturm, G. I. McFadden, S. Sundriyal, J. L. Anglin, Y. Song, S. N. J. Moreno, B. Striepen, Apicoplast isoprenoid precursor synthesis and the molecular basis of fosmidomycin resistance in *Toxoplasma gondii*. *J. Exp. Med.* **208**, 1547–1559 (2011). doi:10.1084/jem.20110039 Medline

24. C. J. Tonkin, N. S. Struck, K. A. Mullin, L. M. Stimmler, G. I. McFadden, Evidence for Golgi-independent transport from the early secretory pathway to the plastid in malaria parasites. *Mol. Microbiol.* **61**, 614–630 (2006). doi:10.1111/j.1365-2958.2006.05244.x Medline

25. R. F. Waller, M. B. Reed, A. F. Cowman, G. I. McFadden, Protein trafficking to the plastid of *Plasmodium falciparum* is via the secretory pathway. *EMBO J.* **19**, 1794–1802 (2000). doi:10.1093/emboj/19.8.1794 Medline

26. J. Meletiadis, S. Pournaras, E. Roilides, T. J. Walsh, Defining fractional inhibitory concentration index cutoffs for additive interactions based on self-drug additive combinations, Monte Carlo simulation analysis, and *in vitro-in vivo* correlation data for antifungal drug combinations against *Aspergillus fumigatus*. *Antimicrob. Agents Chemother.* **54**, 602–609 (2010). doi:10.1128/AAC.00999-09 Medline

27. T. C. Chou, Derivation and properties of Michaelis-Menten type and Hill type equations for reference ligands. *J. Theor. Biol.* **59**, 253–276 (1976). doi:10.1016/0022-5193(76)90169-7 Medline

28. S. Steinbacher, J. Kaiser, W. Eisenreich, R. Huber, A. Bacher, F. Rohdich, Structural basis of fosmidomycin action revealed by the complex with 2-*C*-methyl-D-erythritol 4-phosphate synthase (IspC). Implications for the catalytic mechanism and anti-malaria drug development. *J. Biol. Chem.* **278**, 18401–18407 (2003). doi:10.1074/jbc.M300993200 Medline

29. E. Yeh, J. L. DeRisi, Chemical rescue of malaria parasites lacking an apicoplast defines organelle function in blood-stage *Plasmodium falciparum*. *PLOS Biol.* **9**, e1001138 (2011). doi:10.1371/journal.pbio.1001138 Medline

30. T. Uddin, G. I. McFadden, C. D. Goodman, Validation of putative apicoplast-targeting drugs using a chemical supplementation assay in cultured human malaria parasites. *Antimicrob. Agents Chemother.* **62**, e01161-17 (2017). doi:10.1128/AAC.01161-17 Medline

31. S. E. Lindner, M. J. Sartain, K. Hayes, A. Harupa, R. L. Moritz, S. H. I. Kappe, A. M. Vaughan, Enzymes involved in plastid-targeted phosphatidic acid synthesis are essential for *Plasmodium yoelii* liver-stage development. *Mol. Microbiol.* **91**, 679–693 (2014). doi:10.1111/mmi.12485 Medline

32. G. Pessi, G. Kociubinski, C. B. Mamoun, A pathway for phosphatidylcholine biosynthesis in *Plasmodium falciparum* involving phosphoethanolamine methylation. *Proc. Natl. Acad. Sci. U.S.A.* **101**, 6206–6211 (2004). doi:10.1073/pnas.0307742101 Medline

33. N. Kilian, J. Y. Choi, D. R. Voelker, C. Ben Mamoun, Role of phospholipid synthesis in the development and differentiation of malaria parasites in the blood. *J. Biol. Chem.* **293**, 17308–17316 (2018). doi:10.1074/jbc.R118.003213 Medline

34. X. W. Chan, C. Wrenger, K. Stahl, B. Bergmann, M. Winterberg, I. B. Müller, K. J. Saliba, Chemical and genetic validation of thiamine utilization as an antimalarial drug target. *Nat. Commun.* **4**, 2060 (2013). doi:10.1038/ncomms3060 Medline

35. J. Knöckel, B. Bergmann, I. B. Müller, S. Rathaur, R. D. Walter, C. Wrenger, Filling the gap of intracellular dephosphorylation in the *Plasmodium falciparum* vitamin B1 biosynthesis. *Mol. Biochem. Parasitol.* **157**, 241–243 (2008). doi:10.1016/j.molbiopara.2007.10.010 Medline

36. E. E. Reid, P. Thompson, C. R. Lyttle, D. T. Dennis, Pyruvate dehydrogenase complex from higher plant mitochondria and proplastids. *Plant Physiol.* **59**, 842–848 (1977). doi:10.1104/pp.59.5.842 Medline

37. J. P. Musabyimana, U. Distler, J. Sassmannshausen, C. Berks, J. Manti, S. Bennink, L. Blaschke, P.-C. Burda, A. Flammersfeld, S. Tenzer, C. J. Ngwa, G. Pradel, *Plasmodium falciparum* S-adenosylmethionine synthetase is essential for parasite survival through a complex interaction network with cytoplasmic and nuclear proteins. *Microorganisms* **10**, 1419 (2022). doi:10.3390/microorganisms10071419 Medline

38. Y. Ouyang, Q. Wu, J. Li, S. Sun, S. Sun, S-adenosylmethionine: A metabolite critical to the regulation of autophagy. *Cell Prolif.* **53**, e12891 (2020). doi:10.1111/cpr.12891 Medline

39. H. Choi, D. Fermin, A. I. Nesvizhskii, Significance analysis of spectral count data in label-free shotgun proteomics. *Mol. Cell. Proteomics* **7**, 2373–2385 (2008). doi:10.1074/mcp.M800203-MCP200 Medline

40. Y. Zhang, Z. Wen, M. P. Washburn, L. Florens, Refinements to label free proteome quantitation: How to deal with peptides shared by multiple proteins. *Anal. Chem.* **82**, 2272–2281 (2010). doi:10.1021/ac9023999 Medline

41. C. D. Broeckling, F. A. Afsar, S. Neumann, A. Ben-Hur, J. E. Prenni, RAMClust: A novel feature clustering method enables spectral-matching-based annotation for metabolomics data. *Anal. Chem.* **86**, 6812–6817 (2014). doi:10.1021/ac501530d Medline

42. H. Choi, S. Kim, D. Fermin, C. C. Tsou, A. I. Nesvizhskii, QPROT: Statistical method for testing differential expression using protein-level intensity data in label-free quantitative proteomics. *J. Proteomics* **129**, 121–126 (2015). doi:10.1016/j.jprot.2015.07.036 Medline

43. M. Deponte, H. C. Hoppe, M. C. S. Lee, A. G. Maier, D. Richard, M. Rug, T. Spielmann, J. M. Przyborski, Wherever I may roam: Protein and membrane trafficking in *P. falciparum*-infected red blood cells. *Mol. Biochem. Parasitol.* **186**, 95–116 (2012). doi:10.1016/j.molbiopara.2012.09.007 Medline

44. R. Tuteja, Unraveling the components of protein translocation pathway in human malaria parasite *Plasmodium falciparum*. *Arch. Biochem. Biophys.* **467**, 249–260 (2007). doi:10.1016/j.abb.2007.08.031 Medline

45. A. S. Mamidi, A. Ray, N. Surolia, Structural analysis of *Pf*Sec62-autophagy interacting motifs (AIM) and *Pf*Atg8 interactions for its implications in RecovER-phagy in *Plasmodium falciparum*. *Front. Bioeng. Biotechnol.* **7**, 240 (2019). doi:10.3389/fbioe.2019.00240 Medline

46. H. Fang, N. Green, Nonlethal *sec71-1* and *sec72-1* mutations eliminate proteins associated with the Sec63p-BiP complex from *S. cerevisiae*. *Mol. Biol. Cell* **5**, 933–942 (1994). doi:10.1091/mbc.5.9.933 Medline

47. A. Tripathi, E. C. Mandon, R. Gilmore, T. A. Rapoport, Two alternative binding mechanisms connect the protein translocation Sec71-Sec72 complex with heat shock proteins. *J. Biol. Chem.* **292**, 8007–8018 (2017). doi:10.1074/jbc.M116.761122 Medline

48. D. Martinez Molina, R. Jafari, M. Ignatushchenko, T. Seki, E. A. Larsson, C. Dan, L. Sreekumar, Y. Cao, P. Nordlund, Monitoring drug target engagement in cells and tissues using the cellular thermal shift assay. *Science* **341**, 84–87 (2013). doi:10.1126/science.1233606 Medline

49. J. M. Dziekan, G. Wirjanata, L. Dai, K. D. Go, H. Yu, Y. T. Lim, L. Chen, L. C. Wang, B. Puspita, N. Prabhu, R. M. Sobota, P. Nordlund, Z. Bozdech, Cellular thermal shift assay for the identification of drug-target interactions in the *Plasmodium falciparum* proteome. *Nat. Protoc.* **15**, 1881–1921 (2020). doi:10.1038/s41596-020-0310-z Medline

50. A. Florentin, D. W. Cobb, H. M. Kudyba, V. Muralidharan, Directing traffic: Chaperone-mediated protein transport in malaria parasites. *Cell. Microbiol.* **22**, e13215 (2020). doi:10.1111/cmi.13215 Medline

51. M. M. Savitski, F. B. M. Reinhard, H. Franken, T. Werner, M. F. Savitski, D. Eberhard, D. Martinez Molina, R. Jafari, R. B. Dovega, S. Klaeger, B. Kuster, P. Nordlund, M. Bantscheff, G. Drewes, Tracking cancer drugs in living cells by thermal profiling of the proteome. *Science* **346**, 1255784 (2014). doi:10.1126/science.1255784 Medline

52. C. Nagaraj, N. K. Chakrabarati, M. V. Narasimham, A longitudinal study of *P. falciparum* response to chloroquine in relation to the epidemiology. *J. Commun. Dis.* **21**, 229–240 (1989). Medline

53. T. Masserey, T. Lee, M. Golumbeanu, A. J. Shattock, S. L. Kelly, I. M. Hastings, M. A. Penny, The influence of biological, epidemiological, and treatment factors on the establishment and spread of drug-resistant *Plasmodium falciparum*. *eLife* **11**, e77634 (2022). doi:10.7554/eLife.77634 Medline

54. A. L. Malisa, R. J. Pearce, S. Abdulla, H. Mshinda, P. S. Kachur, P. Bloland, C. Roper, Drug coverage in treatment of malaria and the consequences for resistance evolution—Evidence from the use of sulphadoxine/pyrimethamine. *Malar. J.* **9**, 190 (2010). doi:10.1186/1475-2875-9-190 Medline

55. T. J. Green, G. Gadsden, T. Seed, R. Jacobs, M. Morhardt, R. Brackett, Cloning and characterization of *Plasmodium falciparum* FCR-3/FMG strain. *Am. J. Trop. Med. Hyg.* **34**, 24–30 (1985). doi:10.4269/ajtmh.1985.34.24 Medline

56. S. Siniossoglou, C. Wimmer, M. Rieger, V. Doye, H. Tekotte, C. Weise, S. Emig, A. Segref, E. C. Hurt, A novel complex of nucleoporins, which includes Sec13p and a Sec13p homolog, is essential for normal nuclear pores. *Cell* **84**, 265–275 (1996). doi:10.1016/S0092-8674(00)80981-2 Medline

57. N. Dahan-Pasternak, A. Nasereddin, N. Kolevzon, M. Pe’er, W. Wong, V. Shinder, L. Turnbull, C. B. Whitchurch, M. Elbaum, T. W. Gilberger, E. Yavin, J. Baum, R. Dzikowski, PfSec13 is an unusual chromatin-associated nucleoporin of *Plasmodium*

falciparum that is essential for parasite proliferation in human erythrocytes. *J. Cell Sci.* **126**, 3055–3069 (2013). doi:10.1242/jcs.122119 Medline

58. M. V. Katti, R. Sami-Subbu, P. K. Ranjekar, V. S. Gupta, Amino acid repeat patterns in protein sequences: Their diversity and structural-functional implications. *Protein Sci.* **9**, 1203–1209 (2000). doi:10.1110/ps.9.6.1203 Medline
59. M. B. Jiménez-Díaz, T. Mulet, S. Viera, V. Gómez, H. Garuti, J. Ibáñez, A. Alvarez-Doval, L. D. Shultz, A. Martínez, D. Gargallo-Viola, I. Angulo-Barturen, Improved murine model of malaria using *Plasmodium falciparum* competent strains and non-myelodepleted NOD-*scid IL2Rγ*^{null} mice engrafted with human erythrocytes. *Antimicrob. Agents Chemother.* **53**, 4533–4536 (2009). doi:10.1128/AAC.00519-09 Medline
60. S. Tachibana, S. A. Sullivan, S. Kawai, S. Nakamura, H. R. Kim, N. Goto, N. Arisue, N. M. Q. Palacpac, H. Honma, M. Yagi, T. Tougan, Y. Katakai, O. Kaneko, T. Mita, K. Kita, Y. Yasutomi, P. L. Sutton, R. Shakhbatyan, T. Horii, T. Yasunaga, J. W. Barnwell, A. A. Escalante, J. M. Carlton, K. Tanabe, *Plasmodium cynomolgi* genome sequences provide insight into *Plasmodium vivax* and the monkey malaria clade. *Nat. Genet.* **44**, 1051–1055 (2012). doi:10.1038/ng.2375 Medline
61. S. A. Ralph, G. G. van Dooren, R. F. Waller, M. J. Crawford, M. J. Fraunholz, B. J. Foth, C. J. Tonkin, D. S. Roos, G. I. McFadden, Metabolic maps and functions of the *Plasmodium falciparum* apicoplast. *Nat. Rev. Microbiol.* **2**, 203–216 (2004). doi:10.1038/nrmicro843 Medline
62. N. M. B. Brancucci, J. P. Gerdt, C. Wang, M. De Niz, N. Philip, S. R. Adapa, M. Zhang, E. Hitz, I. Niederwieser, S. D. Boltryk, M.-C. Laffitte, M. A. Clark, C. Grüring, D. Ravel, A. Blancke Soares, A. Demas, S. Bopp, B. Rubio-Ruiz, A. Conejo-Garcia, D. F. Wirth, E. Gendaszewska-Darmach, M. T. Duraisingh, J. H. Adams, T. S. Voss, A. P. Waters, R. H. Y. Jiang, J. Clardy, M. Marti, Lysophosphatidylcholine regulates sexual stage differentiation in the human malaria parasite *Plasmodium falciparum*. *Cell* **171**, 1532–1544.e15 (2017). doi:10.1016/j.cell.2017.10.020 Medline
63. P. K. Sheokand, Y. Yamaryo-Botté, M. Narwal, C.-S. Arnold, V. Thakur, M. M. Islam, M. M. Banday, M. Asad, C. Y. Botté, A. Mohmmed, A *Plasmodium falciparum* lysophospholipase regulates host fatty acid flux via parasite lipid storage to enable controlled asexual schizogony. *Cell Rep.* **42**, 112251 (2023). doi:10.1016/j.celrep.2023.112251 Medline
64. G. G. Holz Jr., Lipids and the malarial parasite. *Bull. World Health Organ.* **55**, 237–248 (1977). Medline
65. B. J. Foth, L. M. Stimmler, E. Handman, B. S. Crabb, A. N. Hodder, G. I. McFadden, The malaria parasite *Plasmodium falciparum* has only one pyruvate dehydrogenase complex, which is located in the apicoplast. *Mol. Microbiol.* **55**, 39–53 (2005). doi:10.1111/j.1365-2958.2004.04407.x Medline
66. A. Mateus, T. A. Määttä, M. M. Savitski, Thermal proteome profiling: Unbiased assessment of protein state through heat-induced stability changes. *Proteome Sci.* **15**, 13 (2017). doi:10.1186/s12953-017-0122-4 Medline

67. Y. Harada, H. Li, J. S. Wall, H. Li, W. J. Lennarz, Structural studies and the assembly of the heptameric post-translational translocon complex. *J. Biol. Chem.* **286**, 2956–2965 (2011). doi:10.1074/jbc.M110.159517 Medline

68. D. W. Chung, N. Ponts, J. Prudhomme, E. M. Rodrigues, K. G. Le Roch, Characterization of the ubiquitylating components of the human malaria parasite's protein degradation pathway. *PLOS ONE* **7**, e43477 (2012). doi:10.1371/journal.pone.0043477 Medline

69. D. Prusty, A. Dar, R. Priya, A. Sharma, S. Dana, N. R. Choudhury, N. S. Rao, S. K. Dhar, Single-stranded DNA binding protein from human malarial parasite *Plasmodium falciparum* is encoded in the nucleus and targeted to the apicoplast. *Nucleic Acids Res.* **38**, 7037–7053 (2010). doi:10.1093/nar/gkq565 Medline

70. R. Prekeris, J. Klumperman, Y. A. Chen, R. H. Scheller, Syntaxin 13 mediates cycling of plasma membrane proteins via tubulovesicular recycling endosomes. *J. Cell Biol.* **143**, 957–971 (1998). doi:10.1083/jcb.143.4.957 Medline

71. L. Ayong, G. Pagnotti, A. B. Tobon, D. Chakrabarti, Identification of *Plasmodium falciparum* family of SNAREs. *Mol. Biochem. Parasitol.* **152**, 113–122 (2007). doi:10.1016/j.molbiopara.2006.12.007 Medline

72. L. A. Parish, J. C. Rayner, *Plasmodium falciparum* secretory pathway: Characterization of PfStx1, a plasma membrane Qa-SNARE. *Mol. Biochem. Parasitol.* **164**, 153–156 (2009). doi:10.1016/j.molbiopara.2008.11.011 Medline

73. J. Y. van der Meer, A. K. Hirsch, The isoprenoid-precursor dependence of *Plasmodium* spp. *Nat. Prod. Rep.* **29**, 721–728 (2012). doi:10.1039/c2np20013a Medline

74. C. Ungermann, D. Kümmel, Structure of membrane tethers and their role in fusion. *Traffic* **20**, 479–490 (2019). doi:10.1111/tra.12655 Medline

75. T. Wang, L. Li, W. Hong, SNARE proteins in membrane trafficking. *Traffic* **18**, 767–775 (2017). doi:10.1111/tra.12524 Medline

76. J. Fu, L. Zhao, Y. Pang, H. Chen, H. Yamamoto, Y. Chen, Z. Li, N. Mizushima, H. Jia, Apicoplast biogenesis mediated by ATG8 requires the ATG12-ATG5-ATG16L and SNAP29 complexes in *Toxoplasma gondii*. *Autophagy* **19**, 1258–1276 (2023). doi:10.1080/15548627.2022.2123639 Medline

77. F. Rohdich, W. Eisenreich, J. Wungsintaweekul, S. Hecht, C. A. Schuhr, A. Bacher, Biosynthesis of terpenoids. 2*C*-Methyl-D-erythritol 2,4-cyclodiphosphate synthase (IspF) from *Plasmodium falciparum*. *Eur. J. Biochem.* **268**, 3190–3197 (2001). doi:10.1046/j.1432-1327.2001.02204.x Medline

78. E. L. Flannery, D. A. Fidock, E. A. Winzeler, Using genetic methods to define the targets of compounds with antimalarial activity. *J. Med. Chem.* **56**, 7761–7771 (2013). doi:10.1021/jm400325j Medline

79. G. LaMonte, M. Y.-X. Lim, M. Wree, C. Reimer, M. Nachon, V. Corey, P. Gedeck, D. Plouffe, A. Du, N. Figueroa, B. Yeung, P. Bifani, E. A. Winzeler, Mutations in the *Plasmodium falciparum* cyclic amine resistance locus (PfCARL) confer multidrug resistance. *mBio* **7**, e00696-16 (2016). doi:10.1128/mBio.00696-16 Medline

80. S. Ménard, T. Ben Haddou, A. P. Ramadani, F. Ariey, X. Iriart, J. Beghain, C. Bouchier, B. Witkowski, A. Berry, O. Mercereau-Puijalon, F. Benoit-Vical, Induction of multidrug tolerance in *Plasmodium falciparum* by extended artemisinin pressure. *Emerg. Infect. Dis.* **21**, 1733–1741 (2015). doi:10.3201/eid2110.150682 Medline

81. F. Ariey, B. Witkowski, C. Amaratunga, J. Beghain, A.-C. Langlois, N. Khim, S. Kim, V. Duru, C. Bouchier, L. Ma, P. Lim, R. Leang, S. Duong, S. Sreng, S. Suon, C. M. Chuor, D. M. Bout, S. Ménard, W. O. Rogers, B. Genton, T. Fandeur, O. Miotto, P. Ringwald, J. Le Bras, A. Berry, J.-C. Barale, R. M. Fairhurst, F. Benoit-Vical, O. Mercereau-Puijalon, D. Ménard, A molecular marker of artemisinin-resistant *Plasmodium falciparum* malaria. *Nature* **505**, 50–55 (2014). doi:10.1038/nature12876 Medline

82. C. Lambros, J. P. Vanderberg, Synchronization of *Plasmodium falciparum* erythrocytic stages in culture. *J. Parasitol.* **65**, 418–420 (1979). doi:10.2307/3280287 Medline

83. W. Trager, J. B. Jensen, Human malaria parasites in continuous culture. *J. Parasitol.* **91**, 484–486 (2005). doi:10.1645/0022-3395(2005)091[0484:HMPICC]2.0.CO;2 Medline

84. A. K. Bei, M. T. Duraisingh, Measuring *Plasmodium falciparum* erythrocyte invasion phenotypes using flow cytometry. *Methods Mol. Biol.* **1325**, 167–186 (2015). doi:10.1007/978-1-4939-2815-6_14 Medline

85. C. Amaratunga, A. T. Neal, R. M. Fairhurst, Flow cytometry-based analysis of artemisinin-resistant *Plasmodium falciparum* in the ring-stage survival assay. *Antimicrob. Agents Chemother.* **58**, 4938–4940 (2014). doi:10.1128/AAC.02902-14 Medline

86. C. J. Joyner, C. F. A. Brito, C. L. Saney, R. Joice Cordy, M. L. Smith, S. A. Lapp, M. Cabrera-Mora, S. Kyu, N. Lackman, M. V. Nural, J. D. DeBarry, J. C. Kissinger, M. P. Styczynski, F. E.-H. Lee, T. J. Lamb, M. R. Galinski; MaHPIC Consortium, Humoral immunity prevents clinical malaria during Plasmodium relapses without eliminating gametocytes. *PLOS Pathog.* **15**, e1007974 (2019). doi:10.1371/journal.ppat.1007974 Medline

87. E. L. Schymanski, J. Jeon, R. Gulde, K. Fenner, M. Ruff, H. P. Singer, J. Hollender, Identifying small molecules via high resolution mass spectrometry: Communicating confidence. *Environ. Sci. Technol.* **48**, 2097–2098 (2014). doi:10.1021/es5002105 Medline

88. L. W. Sumner, A. Amberg, D. Barrett, M. H. Beale, R. Beger, C. A. Daykin, T. W.-M. Fan, O. Fiehn, R. Goodacre, J. L. Griffin, T. Hankemeier, N. Hardy, J. Harnly, R. Higashi, J. Kopka, A. N. Lane, J. C. Lindon, P. Marriott, A. W. Nicholls, M. D. Reily, J. J. Thaden, M. R. Viant, Proposed minimum reporting standards for chemical analysis: Chemical Analysis Working Group (CAWG) Metabolomics Standards Initiative (MSI). *Metabolomics* **3**, 211–221 (2007). doi:10.1007/s11306-007-0082-2 Medline

89. B. MacLean, D. M. Tomazela, N. Shulman, M. Chambers, G. L. Finney, B. Frewen, R. Kern, D. L. Tabb, D. C. Liebler, M. J. MacCoss, Skyline: An open source document editor for creating and analyzing targeted proteomics experiments. *Bioinformatics* **26**, 966–968 (2010). doi:10.1093/bioinformatics/btq054 Medline

90. K. Rajaram, H. B. Liu, S. T. Prigge, Redesigned TetR-aptamer system to control gene expression in *Plasmodium falciparum*. *MSphere* **5**, e00457-20 (2020). doi:10.1128/msphere.00457-20 Medline

91. A. M. Dondorp, F. Nosten, P. Yi, D. Das, A. P. Phyo, J. Tarning, K. M. Lwin, F. Ariey, W. Hanpithakpong, S. J. Lee, P. Ringwald, K. Silamut, M. Imwong, K. Chotivanich, P. Lim, T. Herdman, S. S. An, S. Yeung, P. Singhasivanon, N. P. J. Day, N. Lindegardh, D. Socheat, N. J. White, Artemisinin resistance in *Plasmodium falciparum malaria*. *N. Engl. J. Med.* **361**, 455–467 (2009). doi:10.1056/NEJMoa0808859 Medline

92. J. M. Ribeiro, M. Garriga, N. Potchen, A. K. Crater, A. Gupta, D. Ito, S. A. Desai, Guide RNA selection for CRISPR-Cas9 transfections in *Plasmodium falciparum*. *Int. J. Parasitol.* **48**, 825–832 (2018). doi:10.1016/j.ijpara.2018.03.009 Medline

93. S. H. Adjalley, M. C. Lee, D. A. Fidock, A method for rapid genetic integration into *Plasmodium falciparum* utilizing mycobacteriophage Bxb1 integrase. *Methods Mol. Biol.* **634**, 87–100 (2010). doi:10.1007/978-1-60761-652-8_6 Medline

94. T. Xu, S. K. Park, J. D. Venable, J. A. Wohlschlegel, J. K. Diedrich, D. Cociorva, B. Lu, L. Liao, J. Hewel, X. Han, C. C. L. Wong, B. Fonslow, C. Delahunty, Y. Gao, H. Shah, J. R. Yates 3rd, ProLuCID: An improved SEQUEST-like algorithm with enhanced sensitivity and specificity. *J. Proteomics* **129**, 16–24 (2015). doi:10.1016/j.jprot.2015.07.001 Medline

95. D. L. Tabb, W. H. McDonald, J. R. Yates 3rd, DTASelect and Contrast: Tools for assembling and comparing protein identifications from shotgun proteomics. *J. Proteome Res.* **1**, 21–26 (2002). doi:10.1021/pr015504q Medline

96. Z. Wen, kite: a software suite for processing and analysis of tandem mass spectrometry data. Zenodo (2022); https://doi.org/10.5281/zenodo.5914885.

97. D. A. Fidock, T. Nomura, T. E. Wellems, Cycloguanil and its parent compound proguanil demonstrate distinct activities against *Plasmodium falciparum* malaria parasites transformed with human dihydrofolate reductase. *Mol. Pharmacol.* **54**, 1140–1147 (1998). doi:10.1124/mol.54.6.1140 Medline

98. L. Florens, M. P. Washburn, Proteomic analysis by multidimensional protein identification technology. *Methods Mol. Biol.* **328**, 159–175 (2006). doi:10.1385/1-59745-026-X:159 Medline

99. S. Mnaimneh, A. P. Davierwala, J. Haynes, J. Moffat, W.-T. Peng, W. Zhang, X. Yang, J. Pootoolal, G. Chua, A. Lopez, M. Trochesset, D. Morse, N. J. Krogan, S. L. Hiley, Z. Li, Q. Morris, J. Grigull, N. Mitsakakis, C. J. Roberts, J. F. Greenblatt, C. Boone, C. A. Kaiser, B. J. Andrews, T. R. Hughes, Exploration of essential gene functions via titratable promoter alleles. *Cell* **118**, 31–44 (2004). doi:10.1016/j.cell.2004.06.013 Medline

100. MassIVE MSV000090812, MudPIT analyses of the Plasmodium falciparum proteins pulled down by the kalihinol-analogue, MED6-189, MassIVE (2023); https://doi.org/10.25345/C5ZP3W507.

101. MassIVE MSV000090667, A potent kalihinol analog targeting membrane trafficking inhibits falciparum malaria in humanized mice, MassIVE (2023); https://doi.org/doi:10.25345/C5TM72559.

102. Whole-genome sequencing and RNA-seq files for MED6-189 An effective Antimalarial Kalihinol Analog Targets Plasmodium falciparum Apicoplast and Membrane Biogenesis, PRJNA930408, NCBI BioProject (2023); https://www.ncbi.nlm.nih.gov/bioproject/?term=PRJNA930408.

103. MED6-189: An effective Antimalarial Kalihinol Analog Targets Plasmodium falciparum Apicoplast and Membrane Biogenesis, PanoramaWeb (2023); https://doi.org/10.6069/5a05-5x26.
