# Supplementary material for: A kalihinol analog disrupts apicoplast function and vesicular trafficking in P. falciparum malaria
Source: Science. Author manuscript; Available in PMC 2025 Sep 27. (PMC11793105; doi:10.1126/science.adm7966)
Supplement: MDAR Reproducibility Checklist [file NIHMS2043742-supplement-MDAR_Reproducibility_Checklist.pdf]

Materials Design Analysis Reporting (MDAR)
Checklist for Authors

The MDAR framework establishes a minimum set of requirements in transparent reporting applicable to studies in the life sciences (see Statement of Task: doi:10.31222/osf.io/9sm4x.). The MDAR checklist is a tool for authors, editors, and others seeking to adopt the MDAR framework for transparent reporting in manuscripts and other outputs. Please refer to the MDAR Elaboration Document for additional context for the MDAR framework.

For all that apply, please note where in the manuscript the required information is provided.

Materials:

Newly created materials	indicate where provided: page no/section/legend)	n/a
The manuscript includes a dedicated "materials availability statement" providing transparent disclosure about availability of newly created materials including details on how materials can be accessed and describing any restrictions on access.	Manuscript/Data availability and Code availability sections/Page 17.	

Antibodies	indicate where provided: page no/section/legend)	n/a
For commercial reagents, provide supplier name, catalogue number and RRID, if available.		N/a

DNA and RNA sequences	indicate where provided: page no/section/legend)	n/a
**Short novel DNA or RNA including primers, probes:** Sequences should be included or deposited in a public repository.	**Primer Information:**<br>• Supplementary Tables section/ Table 8/Legends:<br>○ Table S8A(Plasmid construct design and selectable markers present for generation of Sec13-mut lines.)<br>○ Table S8B (Primer sets, gRNA and gene sequence information used Sec13-mut plasmid constructs.)<br>**DNA/RNA Sequencing Information:**<br>• Manuscript/Data availability and Code availability Sections /Page 17.<br>• Also found in Supplementary Tables:<br>○ Table S3A (DEseq2 analysis of drugged and control parasites. All genes for each time point (12, 24, 36, 48, 60 and 72 hours) are indicated.)<br>○ Table S3B (Significant genes obtained by DEseq2 analysis at 24, 60 and 72 hours for drugged and control parasites. No differentially expressed genes were identified at 12, 36 and 48 hours.)<br>○ and Table S7 (Whole genome sequencing results for MED6-189 drug pressured cultures.)	

Cell materials	indicate where provided: page no/section/legend	n/a
**Cell lines:** Provide species information, strain. Provide accession number in repository **OR** supplier name, catalog number, clone number, **OR** RRID.	Supplementary Materials /Materials and Methods Section:<br>• Plasmodium falciparum NF54 strain was provided by the Malaria Research and Reference Reagent Resource Center (MRA-1000)<br>• Plasmodium falciparum 3D7 strain was provided by the Malaria Research and Reference Reagent Resource Center (MRA-102)<br>• Plasmodium falciparum W2 strain was provided by the Malaria Research and Reference Reagent Resource Center (MRA-157)<br>• Plasmodium falciparum HB3 strain was provided by the Malaria Research and Reference Reagent Resource Center (MRA-155)	

	• Plasmodium falciparum Dd2 strain was provided by the Malaria Research and Reference Reagent Resource Center (MRA-156)<br>• Plasmodium falciparum D10-Acp_GFP strain was reposited by the Cowman lab and provided by the Malaria Research and Reference Reagent Resource Center (MRA-568)<br>• Plasmodium knowlesi YH1 strain was provided by Manoj T. Duraisingh (https://doi.org/10.1038/ncomms2612)<br>• S. cerevisiae BY4741 strain (genotype: Mata his3Δ1 leu2Δ0 lys2Δ0 ura3Δ0) was provided by Horizon discovery.<br>• Plasmodium cynomolgi M/B strain was obtained from infected captive bred *Macaca fuscata* of the Oregon National Primate Research Center.<br>• *Plasmodium falciparum* Lines: Page 3<br>• *Plasmodium knowlesi* strain YH1 (provided courtesy of Manoj Duraisingh): Page 3<br>• *Plasmodium cynomolgi* strain M/Bi*:* Pages 3,4<br>• *S. cerevisiae* strain BY4741: Page 13<br>• For *in vivo* efficacy studies, Erythrocyte concentrates from malaria-negative donors were provided by *Biobancos de Castilla y Leon and Centro de Transfusiones de Madrid, Spain.* Page 16	
**Primary cultures:** Provide species, strain, sex of origin, genetic modification status.		N/a

Experimental animals	indicate where provided: page no/section/legend)	n/a
**Laboratory animals or Model organisms:** Provide species, strain, sex, age, genetic modification status. Provide accession number in repository **OR** supplier name, catalog number, clone number, **OR** RRID.	Supplementary Materials /Materials and Methods Section<br>• Male CD1 (20-22g Envigo) mice were used in Toxicology profiles. Page: 14,15<br>• Female NOD-scid IL-2Rgamma null (NSG) mice from GSK Page 16.<br>• *Macaca fuscata* primates were obtained from the Oregon National primate Research Center.<br>•	
**Animal observed in or captured from the field:** Provide species, sex, and age where possible.		N/a

Plants and microbes	indicate where provided: page no/section/legend)	n/a
**Plants:** provide species and strain, ecotype and cultivar where relevant, unique accession number if available, and source (including location for collected wild specimens).		N/a
**Microbes:** provide species and strain, unique accession number if available, and source.	*S. cerevisiae* strain BY4741: Page 13	

Human research participants	indicate where provided: page no/section/legend) or state if these demographics were not collected	n/a
If collected and within the bounds of privacy constraints report on age, sex and gender or ethnicity for all study participants.		N/a

Design:

Study protocol	indicate where provided: page no/section/legend)	n/a
If study protocol has been pre-registered, provide DOI. For clinical trials, provide the trial registration number **OR** cite DOI.		N/a

Laboratory protocol	indicate where provided: page no/section/legend)	n/a
Provide DOI **OR** other citation details if detailed step-by-step protocols are available.	TPP sample preparation and processing:<br>• doi:10.1126/science.1233606<br>• doi:10.1038/s41596-020-0310-z<br>• doi:10.1186/s12953-017-0122-4<br>• doi:10.1126/science.1255784<br>• doi:10.1111/cmi.13215<br><br>Metabolomics/Proteomics data processing and analysis:<br>• doi:10.1016/j.jprot.2015.07.001<br>• doi:10.1021/pr015504q<br>• https://doi.org/10.5281/zenodo.5914885<br>• doi:10.1021/ac9023999<br>• doi:10.1645/0022-3395<br>• doi:10.1124/mol.54.6.1140<br>• doi:10.1385/1-59745-026-X:159<br>• doi:10.1021/ac501530d<br>• doi:10.1021/es5002105<br><br>Generation of Transgenic lines:<br>• doi:10.1016/j.cell.2004.06.013<br>• doi:10.1016/j.ijpara.2018.03.009<br>• doi:10.1073/pnas.96.15.8716<br>• doi:10.1128/mSphere.00457-20<br>• doi:10.1007/978-1-60761-652-8_6<br><br>Phenotypic assays/cell culturing:<br>• doi:10.1007/978-1-4939-2815-6_14<br>• doi:10.1128/AAC.02902-14<br>• doi:10.1645/0022-3395(2005)091<br>• doi:10.1016/j.molbiopara.2007.04.008<br><br>*P. cynomolgi* assays:<br>• doi:10.1038/ng.2375<br>• Moll et al. 2008 (MR4/ATCC, 2008)<br><br>*P. knowlesi* assays:<br>• doi:10.1007/978-1-4939-2815-6_14<br>• doi:10.1128/AAC.02902-14<br><br>Mice model assays:<br>• doi:10.1128/AAC.00519-09	

Experimental study design (statistics details)

**For in vivo studies:** State whether and how the following have been done	**indicate where provided: page no/section/legend. If it could have been done, but was not, write not done**	**n/a**
Sample size determination	Supplementary Materials /Materials and Methods Section<br>• Tolerability study, Page 14<br>• Toxicology Profile, Page 15<br>• In vivo Efficacy, Page 16<br>• In flow cytometry assays: 100,000 erythrocytes were sorted for Plasmodium knowlesi assay. SYBR Green I and Mitotracker Deep Red was used to isolate viable parasites from uninfected erythrocytes. The percent of parasitemia reduction was compared with vehicle-treated control group.<br>• Sample sizes were chosen according to standards in the field and based on our previous experience with similar experiments. For parasitemia, at least 10 fields were counted (300-500 red blood cells) and the phenotypic analyzes were assessed with at least 51 parasites (51 to 106 parasites). At least 25 parasites were observed for IFAs. Sample size and statistical analysis are indicated in the Supplementary Materials /Materials and Methods Section and Source Data.	
Randomisation	Supplementary Materials /Materials and Methods Section<br><br>In vivo Efficacy, Page 16<br>Assignment of parasites were randomized in the study.	
Blinding	The parasitaemia and phenotypic analyzes were performed blind and validated by a third party. Proteomics and metabolomics samples were blinded for our collaborators.	
Inclusion/exclusion criteria		

**Sample definition and in-laboratory replication**	**indicate where provided: page no/section/legend**	**n/a**
State number of times the experiment was replicated in laboratory.	Figure legends for all assays performed describe the number of replicates used in each experimental design.<br>Supplementary Materials /Materials and Methods Section<br>Pages: 4,5,6,11<br>All attempts at replication were successful. Spearman correlation coefficient was performed to validate the reproducibility of RNA-seq Parasitemia, phenotypic analysis, were done in triplicate. Drug combination assays were also performed in triplicates in three to four independent experiments. Growth assays were performed in triplicates in two independent experiments.<br>The metabolomics experiments were performed in triplicate and validated using PCA analysis for each replicate and each condition. IP-MS was performed in triplicate. RNA-seq experiments were performed in duplicate.<br>Parasitemia, phenotypic analysis, and IFAs were done in replicate in at least three independent experiments.	
Define whether data describe technical or biological replicates.	Figure legends for all assays performed describe the number of biological and/or technical replicates	

	used in each experimental design.<br>This data is also found in: Supplementary Materials /Materials and Methods Sections	

Ethics	indicate where provided: page no/section/legend	n/a
**Studies involving human participants:** State details of authority granting ethics approval (IRB or equivalent committee(s), provide reference number for approval.		N/a
**Studies involving experimental animals:** State details of authority granting ethics approval (IRB or equivalent committee(s), provide reference number for approval.	For mouse experiments: All animal studies were ethically reviewed and carried out in accordance with European Directive 2010/63/ EEC and the GSK Policy on the Care, Welfare and Treatment of Animals.<br>For studies performed in macaques: All animal studies were ethically reviewed and carried out in accordance with the Association for Assessment and Accreditation of Laboratory Animal Care institution (AAALAC) and housed in compliance with the Guide and Care and Use of Laboratory animals.	
**Studies involving specimen and field samples:** State if relevant permits obtained, provide details of authority approving study; if none were required, explain why.		N/a

Dual Use Research of Concern (DURC)	indicate where provided: page no/section/legend	n/a
If study is subject to dual use research of concern regulations, state the authority granting approval and reference number for the regulatory approval.		N/a

Analysis:

Attrition	indicate where provided: page no/section/legend	n/a
Describe whether exclusion criteria were preestablished. Report if sample or data points were omitted from analysis. If yes report if this was due to attrition or intentional exclusion and provide justification.		n/a

Statistics	indicate where provided: page no/section/legend	n/a
Describe statistical tests used and justify choice of tests.	All data regarding found in: Supplementary Materials /Materials and Methods Sections and Code availability sections of the manuscript.<br>Figure legends describe the statistical tests performed in each experimental design.<br>Data analysis and statistical tests were performed in accordance with published scientific resources and using programs listed below:<br>• ImageJ (version 1.53i)<br>• GraphPad Prism version 9.1.2<br>• R package<br><br>See Supplementary Materials /Materials and Methods Sections	

Data availability	indicate where provided: page no/section/legend	n/a
For newly created and reused datasets, the manuscript includes a data availability statement that provides details for access or notes restrictions on access.	All data regarding found in: Supplementary Materials /Materials and Methods Sections and Code availability sections of the manuscript Page: 24-25	
If newly created datasets are publicly available, provide accession number in repository **OR** DOI **OR** URL and licensing details where available.		
If reused data is publicly available provide accession number in repository **OR** DOI **OR** URL, **OR** citation.		

Code availability	indicate where provided: page no/section/legend	n/a
For all newly generated custom computer code/software/mathematical algorithm or re-used code essential for replicating the main findings of the study, the manuscript includes a data availability statement that provides details for access or notes restrictions.	All newly generated data are available In the Manuscript Pages: 24-25<br>In the Supplemental Tables: Table S3, S4, S6<br>And listed below:<br><br>• WGS and RNA-seq datasets generated in this study have been deposited in the NCBI BioProject database under SubmissionID: SUB12241156 with BioProject ID: PRJNA930408<br>• Proteomics datasets generated in this study have been deposited in the proteomeXchange archive under accession number: (PXD038457 and PXD038053) as well as MassIVE repository under accession number:(MSV000090812 and MSV00090667) with password: kalihinol<br>• Metabolomics datasets generated in this study are available at Panorama and can be accessed through the link (https://panoramaweb.org/qpjX5B.url) using accession codes:	
If newly generated code is publicly available, provide accession number in repository, **OR** DOI **OR** URL and licensing details where available. State any restrictions on code availability or accessibility.		N/a

If reused code is publicly available provide accession number in repository **OR** DOI **OR** URL, **OR** citation.		N/a

Reporting

MDAR framework recommends adoption of discipline-specific guidelines, established and endorsed through community initiatives. Journals have their own policy about requiring specific guidelines and recommendations to complement MDAR.

Adherence to community standards	indicate where provided: page no/section/legend	n/a
State if relevant guidelines (e.g., ICMJE, MIBBI, ARRIVE) have been followed, and whether a checklist (e.g., CONSORT, PRISMA, ARRIVE) is provided with the manuscript.		N/a
